# Genome-Wide Comparative In Silico Analysis of the RNA Helicase Gene Family in *Zea mays* and *Glycine max*: A Comparison with *Arabidopsis* and *Oryza sativa*

**DOI:** 10.1371/journal.pone.0078982

**Published:** 2013-11-12

**Authors:** Ruirui Xu, Shizhong Zhang, Jinguang Huang, Chengchao Zheng

**Affiliations:** 1 Key Laboratory of Biology and Molecular Biology in University of Shandong, Weifang University, Weifang, Shandong, P.R. China; 2 State Key Laboratory of Crop Biology, Shandong Agricultural University, Taian, Shandong, P.R. China; Institute of Botany, Chinese Academy of Sciences, China

## Abstract

RNA helicases are enzymes that are thought to unwind double-stranded RNA molecules in an energy-dependent fashion through the hydrolysis of NTP. RNA helicases are associated with all processes involving RNA molecules, including nuclear transcription, editing, splicing, ribosome biogenesis, RNA export, and organelle gene expression. The involvement of RNA helicase in response to stress and in plant growth and development has been reported previously. While their importance in *Arabidopsis* and *Oryza sativa* has been partially studied, the function of RNA helicase proteins is poorly understood in *Zea mays* and *Glycine max*. In this study, we identified a total of RNA helicase genes in *Arabidopsis* and other crop species genome by genome-wide comparative in silico analysis. We classified the RNA helicase genes into three subfamilies according to the structural features of the motif II region, such as DEAD-box, DEAH-box and DExD/H-box, and different species showed different patterns of alternative splicing. Secondly, chromosome location analysis showed that the RNA helicase protein genes were distributed across all chromosomes with different densities in the four species. Thirdly, phylogenetic tree analyses identified the relevant homologs of DEAD-box, DEAH-box and DExD/H-box RNA helicase proteins in each of the four species. Fourthly, microarray expression data showed that many of these predicted RNA helicase genes were expressed in different developmental stages and different tissues under normal growth conditions. Finally, real-time quantitative PCR analysis showed that the expression levels of 10 genes in *Arabidopsis* and 13 genes in *Zea mays* were in close agreement with the microarray expression data. To our knowledge, this is the first report of a comparative genome-wide analysis of the RNA helicase gene family in *Arabidopsis, Oryza sativa*, *Zea mays* and *Glycine max*. This study provides valuable information for understanding the classification and putative functions of the RNA helicase gene family in crop growth and development.

## Introduction

Helicases have been identified in organisms ranging from *Escherichia coli* to humans, viruses and plants. The helicases from all of these organisms represent a large gene family that may have a predominant role in modulating environmental responses. These proteins can be grouped into families based on sequence homologies [1], [2]. The RNA helicases are enzymes that use energy derived from the hydrolysis of a nucleotide triphosphate to unwind double-stranded RNAs [3]. The majority of RNA helicases belong to superfamily 2 (SF2), which consists of three subfamilies, known as DEAD, DEAH and DExD/H [4], [5], [6]. RNA helicases have been shown to be involved in every step of RNA metabolism, including nuclear transcription, pre-mRNA splicing, ribosome biogenesis, nucleocytoplasmic transport, translation, RNA decay, and organellar gene expression [3], [4], [7]. Based on the multiple functions of these genes in cellular RNA metabolism, the fact that the RNA helicases are also involved in plant growth and development and the response to abiotic stress is not surprising.

The RNA helicase family in plants is larger and more diverse than in other systems [8]. In fact, the expression level of several DEAD-box helicases has been shown to be regulated in response to changes in specific environmental conditions, including salt stress, oxygen levels, light or temperature [2], [9], [10]. Initially, three *Arabidopsis* DEAD-box RNA helicases, *LOS4*, *STRS1* and *STRS2*, were shown to be involved in the stress responses to various abiotic stresses [11], [12], [13]. *HUA ENHANCER2*, encoding a putative DExH-box RNA helicase, was shown to be active in both B and C pathways in the flower and to affect vegetative and inflorescence development in *Arabidopsis* [14]. A plastid DEAD-box RNA helicase *VDL* gene in tobacco was reported to play an important role in chloroplast differentiation and plant morphogenesis [15]. The *Arabidopsis* DExH box helicase CAF/DICER-LIKE 1 has been shown to be critical for the biogenesis of microRNAs and plant development [16], [17]. In *Zea mays*, MA16, fibrillarin, and ZmDRH1 may be part of a ribonucleoprotein complex involved in ribosomal RNA metabolism [18]. *Arabidopsis TEBICHI* has been shown to be required for the regulation of cell division and differentiation in meristems [19], and *ISE2*, which is localised to cytoplasmic granules, has been shown to be involved in plasmodesmata function during embryogenesis in *Arabidopsis* [20]. OsBIRH1, the rice homolog of RH50, exhibited RNA helicase activities in vitro and helped to confer plant resistance against various stresses [21]. Another RNA helicase, PUTATIVE MITOCHONDRIAL RNA HELICASE2, is involved in group II intron splicing in mitochondria [22]. Additionally, *SLOW WALKER3* has been shown to be essential for female gametogenesis as a putative DEAD-box RNA helicase in *Arabidopsis* [23]. Previously, we reported that the DEVH-box RNA helicase AtHELPS participates in the regulation of potassium-deprivation tolerance [24]. Recently, rice API5 has been shown to couple with two DEAD-box RNA helicases (API1 and API2) in regulating PCD during tapetum degeneration in rice [25]. A DExH-box RNA helicase, ABO6, was shown to mediate mitochondrial reactive oxygen species production and also mediate crosstalk between ABA and Auxin signalling [26]. Cumulatively, these investigations indicate that the RNA helicases may play an important role in building resistance to abiotic stresses and in plant growth and development.

Despite the diversity of their biological functions and the wide range of organisms in which these proteins have been identified, high sequence conservation has been maintained in the large group of helicases, suggesting that all the helicase genes evolved from a common ancestor. Hence, signature sequences can be used efficiently for the detection and the prediction of new helicases in the genome databases [27]. After a thorough analysis of all the sequence data, 32 different DEAD-box RNA helicases have been identified and named from AtRH1 to AtRH32 [27]. Subsequently, the analysis of RNA helicase genes in *Escherichia coli* have been identified and studied extensively, including 5 DEAD-box and 13 DExH-box RNA helicase genes [28], [29]. Recently, the complete analysis and classification of the RNA helicase gene family in *Arabidopsis* and *Oryza sativa,* which contain 113 and 115 RNA helicase genes, respectively, has been reported [2].

These studies added more evidence of the role of RNA helicase genes in development and response to abiotic stresses in different species, but related genome-wide resources are limited in two other important crops, maize and soybean. In addition, several whole-genome analysis studies in *Arabidopsis* and rice have been performed in the past ten years, focused on categories such as the RING-finger, receptor-like kinase gene family, MAPK and MAPKK gene family, KT/HAK/KUP potassium transporters gene family, NAC proteins and siRNAs [30], [31], [32], [33], [34], [35], [36], [37]. Recently, genome-wide analysis in maize has identified and analysed most of the cyclins, *MuDR*-related transposable elements, beta-glucosidase gene family, auxin response factor (ARF) gene family and two-component signal system (TCS) genes [38], [39], [40], [41], [42]. So far, no genome-wide information on the RNA helicase gene family is currently available in *Zea mays* or *Glycine max*. Extensive genome-wide comparative in silico analysis of the RNA helicase gene family in the completed genomes of *Zea mays* and *Glycine max* could identify numerous known or novel gene families associated with defence, photomorphogenesis, gene regulation, development, metabolism, transportation and/or stress tolerance.

In this study, we identified a total 161, 149, 136 and 213 RNA helicase genes in the *Arabidopsis*, *Oryza sativa*, *Zea mays* and *Glycine max* genomes, respectively, by genome-wide comparative in silico analysis. Each of the different subfamilies, such as DEAD-box, DEAH-box or DExD/H-box, has different rate of alternative splicing in each species. The chromosome location analysis showed that the RNA helicase protein genes were distributed across all chromosomes with different densities in the four species. The phylogenetic tree analyses identified the relevant homologs of DEAD-box, DEAH-box and DExD/H-box RNA helicase proteins in each of the four species. Additionally, microarray expression data showed that many of these predicted RNA helicase genes were expressed in different developmental stages and different tissues under normal growth conditions. Finally, real-time quantitative PCR analysis showed that the expression levels of 10 RNA helicase genes in *Arabidopsis* and 13 RNA helicase genes in *Zea mays* were in close agreement with the microarray expression data. To our knowledge, this is the first report of a comparative genome-wide analysis of the RNA helicase gene family in *Arabidopsis, Oryza sativa*, *Zea mays* and *Glycine max*. The comparative genome-wide analysis provides valuable information for understanding the classification and putative functions of the RNA helicase gene family and new insight into the organisation, evolution and functions of the RNA helicase gene family in crop growth and development.

## Results

### The Identification of the RNA Helicase genes in *Arabidopsis*, *Oryza sativa*, *Zea Mays* and *Glycine Max*

To identify the members of the RNA helicase gene family in *Arabidopsis*, *Oryza sativa*, *Zea mays* and *Glycine max*, we used bioinformatic methods to gather extensive information regarding this family. A total of 161 genes that encode 217 RNA helicase proteins were identified as potential members of the RNA helicase superfamily within the *Arabidopsis* genome (http://www.tair.org/), whereas 149 genes encoded 199 RNA helicase proteins were identified in the *Oryza sativa* genome (http://www.phytozome.net/) (Table S1). Our predicted number of RNA helicase proteins in the two species was greater than the number found in *Arabidopsis* (113) or rice (115) [2]. So far, predicted members of the RNA helicase gene family in *Glycine max* and *Zea mays* have not reported in detail. In our results, we identified a total of 136 and 213 RNA helicase genes in the *Zea mays* and *Glycine max* genome (http://www.phytozome.net/), respectively (Table S1).

Based on the characteristics of the conserved motifs, the RNA helicase genes were classified into three subfamilies: DEAD-box (50/51/57/87 genes), DEAH-box (40/33/31/48 genes) and DExD/H-box (71/65/50/78 genes) in the four species *Arabidopsis*, *Oryza sativa*, *Zea mays* and *Glycine max* (Table 1), respectively for each subfamily. In addition, the results revealed that *Arabidopsis*, *Oryza sativa*, *Zea mays* and *Glycine max* have 56, 50, 79 and 35 alternative splicing in whole RNA helicase gene family, respectively (Table 1). In *Zea mays*, there are two particular genes whose different alternative splicing products belong to different RNA helicase subfamilies. GRMZM2G010085_T01 and GRMZM2G010085_T02 belong to the DEAH-box and DExD/H-box subfamilies, respectively. Moreover, GRMZM2G420865_T02 and GRMZM2G420865_T03 belong to DEAD-box and DExD/H-box, respectively.

**Table 1:** The number of the DEAD-box, DEAH-box and DExD/H-box RNA helicase genes in *Arabidopsis*, *Oryza sativa*, *Zea mays*, *Glycine max*.

Species	DEAD-box	DEAH-box	DExD/H-box	Total	Alternative Splicing	Alternative Splicing (%)
*Arabidopsis*	50/66	40/52	71/99	161/217	56	25.81
*Oryza sativa*	51/79	33/41	65/79	149/199	50	25.12
*Zea mays*	57/86	31/44	50/85	136*/215	79	36.74
*Glycine max*	87/101	48/55	78/92	213/248	35	14.11

Note: In front of diagonal not include the number of alternative splicing; in the back of diagonal include the number of alternative splicing.

* In *Zea mays*, there are two especial genes with different alternative splicing belongs to different RNA helicase subfamilies.

### The Chromosome Localization of the RNA Helicase Gene Family in *Arabidopsis*, *Oryza sativa*, *Zea Mays* and *Glycine Max*

**Figure 1:**
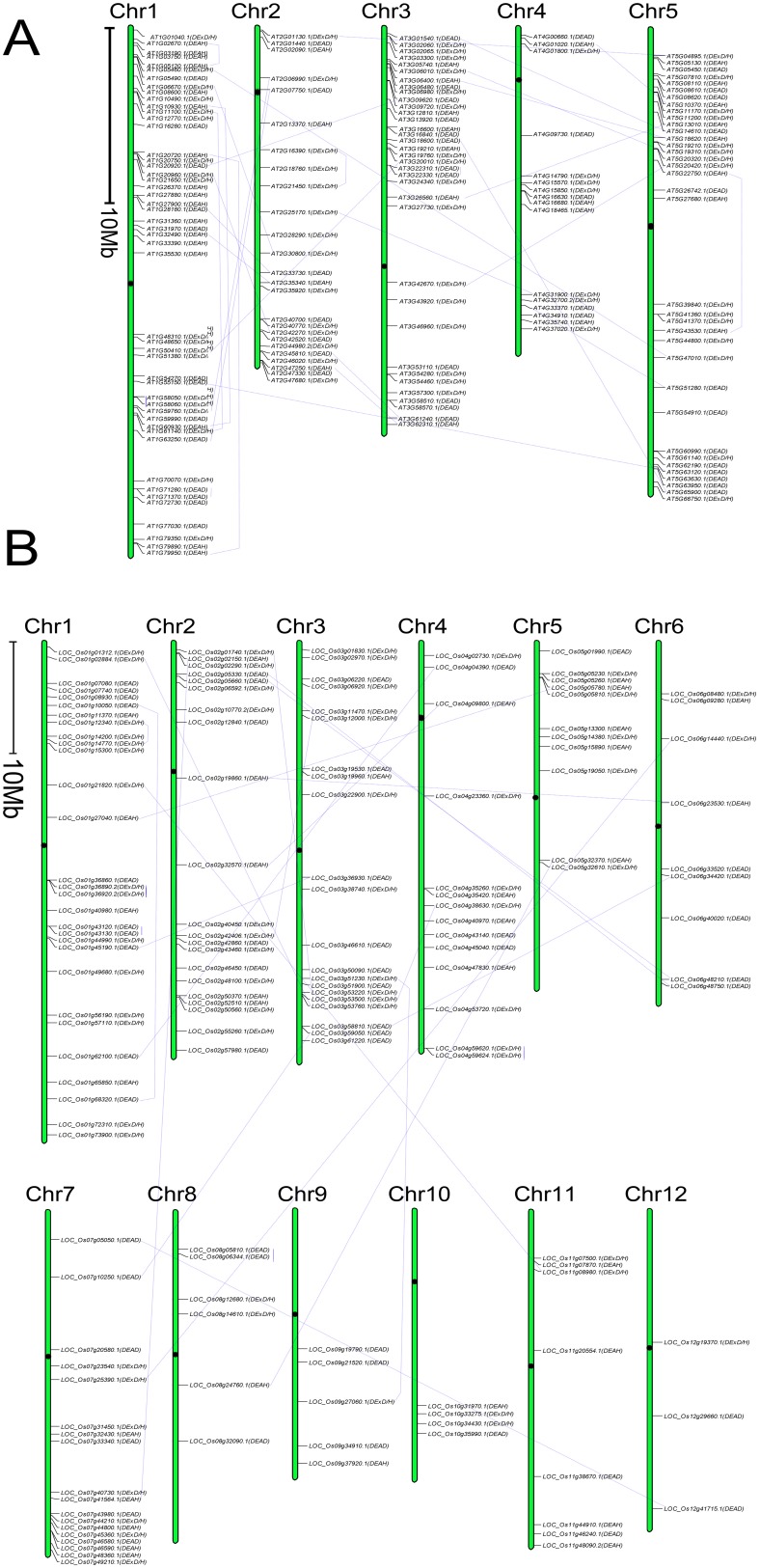
The chromosomal mapping of the RNA helicase gene family. (A) *Arabidopsis*, (B) *Oryza sativa*. The scale bar represents a 10.0 Mb chromosomal distance. The chromosome number is indicated at the top of each chromosome. To simplify the presentation, we named the putative RNA helicase genes with their own Gene ID. The subfamily of each RNA helicase gene was shown in parentheses. The paralogous sister pairs of RNA helicase genes were connected by light blue line, which had very strong bootstrap support (>90%). The positions of centromeres were marked with the black dot.

**Figure 2:**
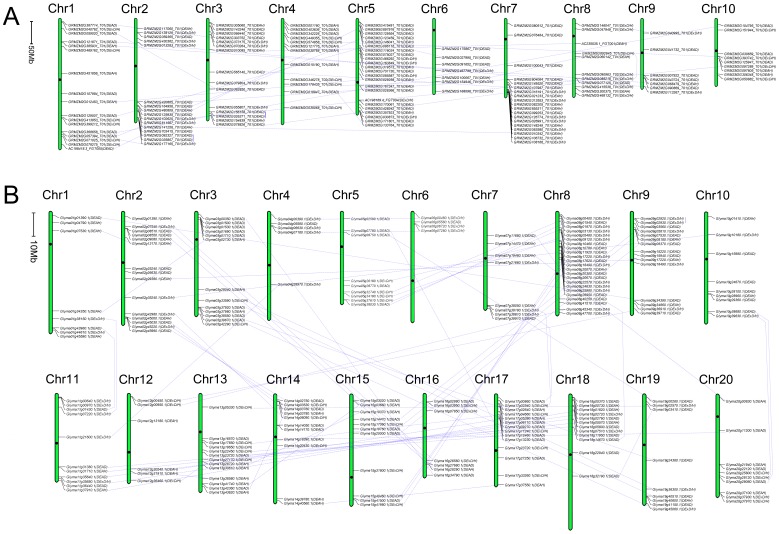
The chromosomal mapping of the RNA helicase gene family. (A) *Zea mays*, (B) *Glycine max*. The scale bar represents a 10.0 Mb chromosomal distance. The chromosome number is indicated at the top of each chromosome. To simplify the presentation, we named the putative RNA helicase genes with their own Gene ID. The subfamily of each RNA helicase gene was shown in parentheses. The paralogous sister pairs of RNA helicase genes were connected by light blue line, which had very strong bootstrap support (>90%). The positions of centromeres were marked with the black dot.

Using the Perl-based program MapDraw and Photoshop tools, the RNA helicase genes were then mapped onto the chromosomes of different species and named with their own Gene ID. In all four species, all predicted RNA helicase genes could be conclusively matched to a chromosome (Figure 1 and Figure 2). The chromosomal locations of 161 *Arabidopsis* RNA helicase protein genes were analysed first in our study. The chromosomes locations analysis showed that the *Arabidopsis* RNA helicase protein genes were distributed across all 5 chromosomes with different densities from 9.9% (chromosome 4) to 30.4% (chromosome 1) (Figure 1A). Second, we found a similar distribution pattern on *Oryza sativa* chromosomes from 2.0% (chromosome 12) to 19.5% (chromosome 1) (Figure 1B). Only 3 genes were mapped on chromosome 12. In addition, the *Zea mays* and *Glycine max* RNA helicase protein genes were mapped on the chromosomes from chromosomes 1 to 10 and from chromosomes 1 to 20, respectively. In *Zea mays*, chromosome 5 encompassed the most RNA helicase protein genes with 23 (16.9%), while chromosomes 6 and 9 contained 7 RNA helicase protein genes (5.1%) (Figure 2A). Compared with the preceding three species, relatively low densities of RNA helicase protein genes were observed on the 20 *Glycine max* chromosomes, with the densities from 1.9% (chromosome 6 ) to 10.8% (chromosome 8) (Figure 2B). To detect possible relationships between RNA helicase genes and potential genome duplication events, we mapped 35, 27, 25 and 62 paralogous gene pairs of RNA helicase genes in *Arabidopsis*, *Oryza sativa*, *Zea mays* and *Glycine max*, respectively (Figure 1 and Figure 2). It is noteworthy that the percentage of the paralogous gene pairs of RNA helicase genes in *Glycine max* was higher than the other three species, indicating that segmental and/or tandem duplications might more frequently occurred in *Glycine max*.

### The Phylogenetic Tree Analysis of the RNA Helicase Gene Family in *Arabidopsis*, *Oryza Sativa*, *Zea Mays* and *Glycine Max*

**Figure 3:**
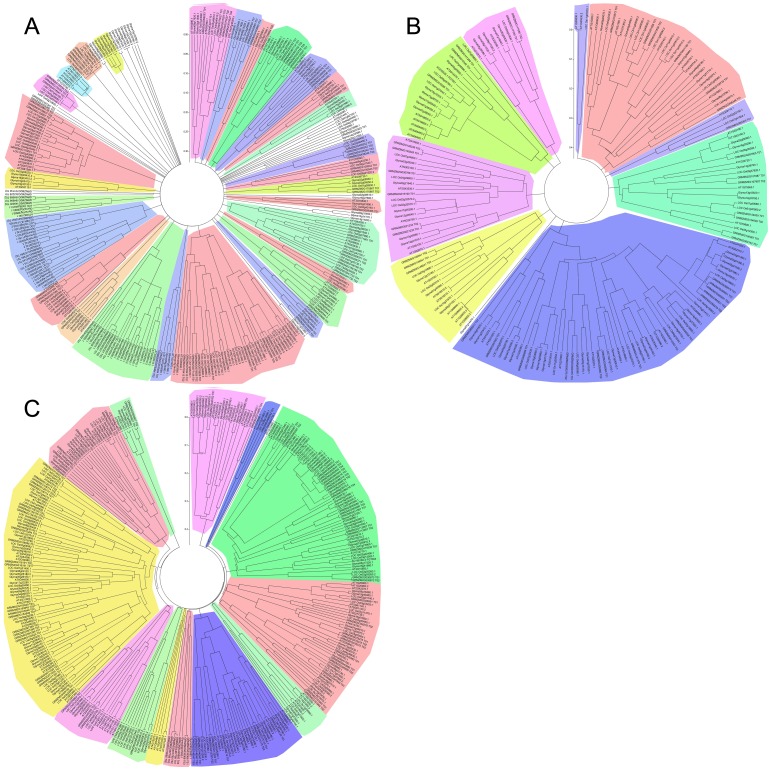
Phylogenetic tree analysis of RNA helicase in *Arabidopsis*, *Oryza sativa*, *Zea mays* and *Glycine max*. (A) The DEAD-box RNA helicase proteins in four species. (B) The DEAH-box RNA helicase proteins in four species. (C) The DExD/H-box RNA helicase proteins in four species. The amino acid sequences of the RNA helicase proteins were aligned with ClustalX, and the phylogenetic tree was constructed using the neighbour-joining method in MEGA 5.0 software. Each node is represented by a number that indicates the bootstrap value for 100 replicates. The scale bar represents 0.05, 0.1 and 0.1 substitutions per sequence position, respectively.

To determine their evolutionary relationship, the phylogenetic relationship of each subfamily of the RNA helicase proteins was examined by aligning their amino acid sequences and implementing the neighbour-joining method in MEGA 5.0. The phylogenetic tree analyses showed that the whole of DEAD-box (332), DEAH-box (192) and DExD/H-box (355) RNA helicase proteins in four species, respectively (Figure 3). We also performed the orthologs/paralogs relationship among the RNA helicase genes family in four species (Figure S1, Figure S2, Figure S3 and Figure S4). Three *Arabidopsis* DEAD-box RNA helicases, *LOS4*, *STRS1* and *STRS2*, were shown to be involved in responses to multiple abiotic stresses [11], [12], [13]. These investigations indicate that DEAD-box RNA helicases may play an important role in building resistance to abiotic stress during plant growth and development. Figure 3A shows that Glyma18g32190, Glyma19g03410 and LOC_Os03g6220 have high homology to *Arabidopsis* LOS4 (At3G53110). In addition, the phylogenetic tree analyses showed that STRS1 (At1G31970) has a high level of identity to Glyma01g01390, Glyma09g34390, LOC_Os07g20580 and GRMAZM2G076484. An analysis of the DEAH-box RNA helicase proteins showed that this subfamily could be further classified into nine subgroups (Figure 3B), whereas the other two subfamilies, DEAD-box and DExD/H-box RNA helicase proteins, could be further classified into more than ten subgroups (Figure 3A and Figure 3C). *ISE2* and *AtHELPS*, encoding DExD/H-box RNA helicases, were shown to be involved in plasmodesmata function during embryogenesis and potassium deprivation responses and tolerance in *Arabidopsis thaliana*, respectively [20], [24]. As shown in Figure 3C, we identified the relevant homologs of *ISE2* (AT1G70070) and *AtHELPS* (AT3G46960) as LOC_Os02g50560 and Glyma18g07510, respectively. At the same time, the phylogenetic tree analyses revealed that OsBIRH1 (LOC_Os03g01830) has a higher homology to *Arabidopsis* AT3G06980, which is in accord with preceding research [21].

### The Expression Profile During Different Development Stage and Different Tissues of the RNA Helicase Genes in Four Species Under Normal Growth Conditions

**Figure 4:**
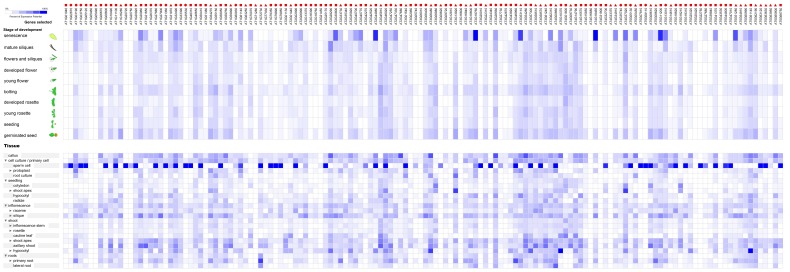
The expression profiles of 145 RNA helicase genes in *Arabidopsis*. The expression profiles of 145 RNA helicase genes in different development stages (above) and tissues (below) in *Arabidopsis*. The deep and light blue shading represents the relative high or low percent potential expression levels, respectively, of the helicase genes in different development stages and different tissues. The DEAD-box, DEAH-box and DExD/H-box RNA helicase genes are indicated by the red triangle, circle and square, respectively.

To investigate the potential functions of the RNA helicase proteins in crop development, we analysed microarray expression data from various datasets in the Gene Chip platform of Genevestigator. We found that not all of the predicted genes were expressed in different plant developmental stages and different tissues under normal growth conditions. As shown in Figure 4, among the 161 predicted genes in *Arabidopsis*, 144 genes (89.4%) were expressed in at least one of the development stages tested. More than half of the predicted RNA helicase genes were expressed in ten different development stages with various expression levels, including senescence, mature siliques, flowers and siliques, developed flower, young flower, bolting, developed rosette, young rosette, seedling and germinated seed. *AT2G44980*, *AT3G19760*, *AT3G53110* (*LOS4*), *AT4G18465*, *AT5G11200* and *AT5G51280* were highly expressed in senescence stage. Our results showed that the most RNA helicase genes were expressed in more than 20 tissues in *Arabidopsis* with different expression levels and many predicted *Arabidopsis* RNA helicase genes were expressed in primary cell, seedling, inflorescence, silique, shoot and roots. Forty-four genes were highly expressed in sperm cells and *AT5G10370* and *AT5G61140* were only highly expressed in primary root tissue (Figure 4).

**Figure 5:**
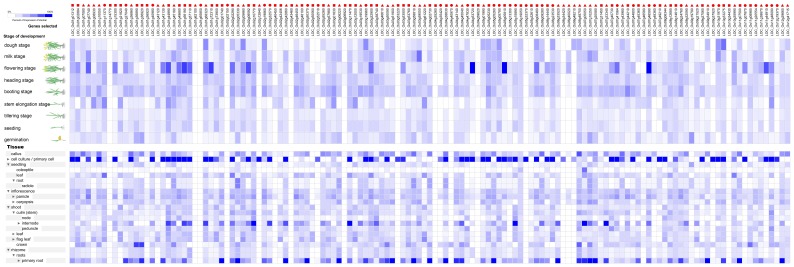
The expression profiles of 138 RNA helicase genes in *Oryza sativa*. The expression profiles of 138 RNA helicase genes in different development stages (above) and different tissues (below) in *Oryza sativa*. The deep and light blue shading represents the relative high or low percent potential expression levels, respectively, of the helicase genes in different development stages and different tissues. The DEAD-box, DEAH-box and DExD/H-box RNA helicase genes are indicated by the red triangle, circle and square, respectively.

During the nine *Oryza sativa* development stages, including the dough stage, milk stage, flowering stage, heading stage, booting stage, stem elongation stage, tillering stage, seedling and germination, 135 genes (90.6%) were expressed in at least one of the development stages tested and several genes had high expression levels in the flowering stage (Figure 5). Notably, the expression levels of the RNA helicase in all tested *Oryza sativa* tissues were higher in primary cell, internode and primary root (Figure 5).

**Figure 6:**
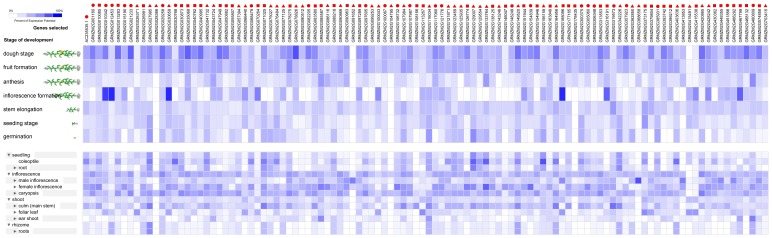
The expression profiles of 54 RNA helicase genes in *Zea mays*. The expression profiles of 54 RNA helicase genes in different development stages (above) and different tissues (below) in *Zea mays*. The deep and light blue shading represents the relative high or low percent potential expression levels, respectively, of the helicase genes in different development stages and different tissues. The DEAD-box, DEAH-box and DExD/H-box RNA helicase genes are indicated by the red triangle, circle and square, respectively.

Approximately 109 *Zea mays* genes were expressed with various expression levels in the tested development stages, including dough stage, fruit formation, anthesis, inflorescence formation, stem elongation, seedling stage and germination (Figure 6). In addition, the expression of these genes exhibited similar profiles and showed higher expression level than in *Arabidopsis* and *Oryza sativa* development stage (Figure 6).

**Figure 7:**
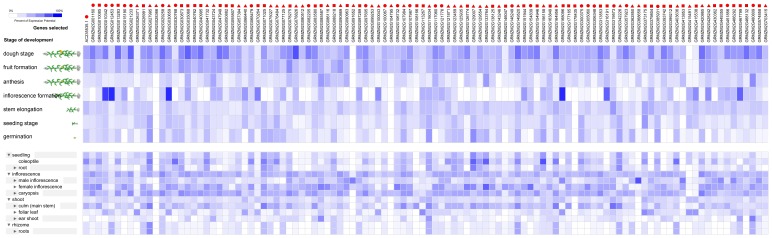
The expression profiles of 99 RNA helicase genes in *Glycine max*. The expression profiles of 99 RNA helicase genes in different development stages (above) and different tissues (below) in *Glycine max*. The deep and light blue shading represents the relative high or low percent potential expression levels, respectively, of the helicase genes in different development sages and different tissues. The DEAD-box, DEAH-box and DExD/H-box RNA helicase genes are indicated by the red triangle, circle and square, respectively.

Approach to half of the *Glycine max* RNA helicase genes were expressed highly in fruit formation and bean development stage. For another, about half of the genes were expressed in main shoot growth and germination at lower levels. Moreover, approximately 15 genes were most highly expressed in the flowering stage (Figure 7). Many genes exhibited higher expression levels in the primary cell, leaf cell, shoot apex, axillary meristem, shoot apex and unspecified root type of soybean (Figure 7). Taken together, these data suggest that the highly expressed RNA helicase genes may play an important role in the regulation of four species’ growth and development, and these analyses further aid the understanding of the basal functions of many RNA helicase proteins in crop growth and development.

### The Expression Profile of the RNA Helicase Genes in Various Tissues as Determined by qRT-PCR Analyses in *Arabidopsis* and *Zea Mays*

**Figure 8:**
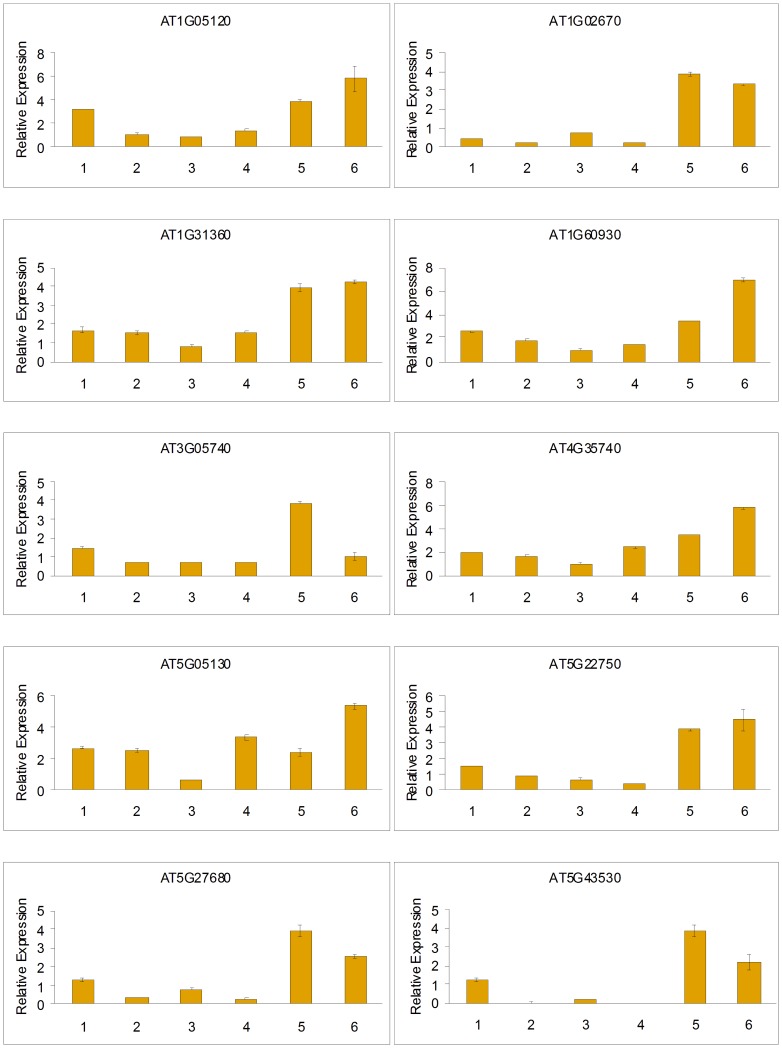
QRT-PCR analysis of 10 helicase genes in different tissues of *Arabidopsis*. Error bars indicate standard deviations (n = 3). 1, root; 2, rosette leaf; 3, stem; 4, cauline leaf; 5, flower; 6, silique.

We performed the expression analysis of 10 RNA helicase genes in *Arabidopsis* under normal growth conditions in six different tissues: root, rosette leaf, stem, cauline leaf, flower and silique. Basically in accord with Genevestigator analysis, qRT-PCR results showed that 9 predicted genes were expressed in all of the tested tissues in *Arabidopsis* (Figure 8). Only one gene (*AT5G43530*) displayed tissue-specific expression patterns, which was not detected in rosette leaf and cauline leaf (Figure 8). Intriguingly, relatively higher expression levels of these helicase genes were observed in flower and silique, indicating that RNA helicase activities might be closely related with reproductive processes in *Arabidopsis* (Figure 8).

**Figure 9:**
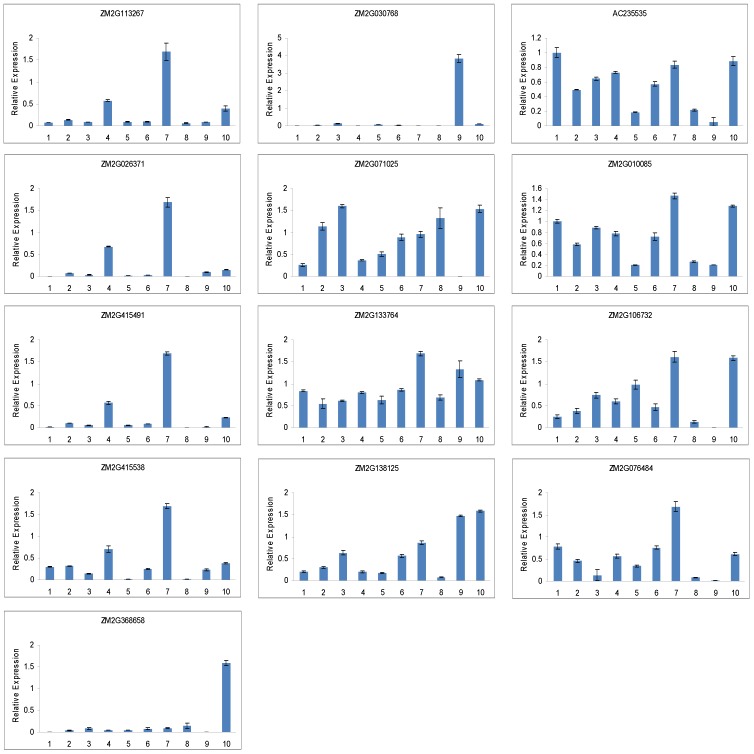
QRT-PCR analysis of 13 helicase genes in different tissues of *Zea mays*. Error bars indicate standard deviations (n = 3). 1, primary root; 2, pericarp; 3, internode; 4, adult leaf; 5, silk; 6, culm; 7, seedling; 8, endosperm; 9, embryo; 10, tassel.

We also analysed the expression of 13 RNA helicase genes in maize under normal growth conditions in ten different tissues: primary root, pericarp, internode, adult leaf, silk, culm, seedling, endosperm, embryo and tassel. All of the 13 predicted genes were expressed in at least one of the ten tissues in maize (Figure 9). The results showed that *ZM2G113267*, *ZM2G026371*, *ZM2G415491* and *ZM2G415538* were primarily expressed in the seedlings and adult leaf, while they were barely expressed in the other eight tissues. *ZM2G368658* and *ZM2G030768* were especially abundant in the embryo and tassel, respectively, and were expressed at relatively low levels in other nine tissues. *ZM2G071025*, *ZM2G133764*, *ZM2G138125*, *AC235535*, *ZM2G010085*, *ZM2G106732* and *ZM2G076484* were detected in all tested tissues, whereas *ZM2G071025* and *AC235535* in the embryo, *ZM2G138125* in the endosperm, *ZM2G010085*, Z*M2G106732* and *ZM2G076484* in the endosperm and embryo were weakly expressed (Figure 9). These results, also in accord with Genevestigator analysis, suggest that the tested 13 RNA helicase genes might be involved in tissue development in maize. Taken together, these results imply that the tested RNA helicase genes might play roles in regulating the development of different tissues.

## Discussion

RNA helicases are found in various organisms, ranging from prokaryotes to mammals, and have become a focus of interest in recent years due to their participation in diverse cellular processes [8], [43], [44]. In the past ten years, although the RNA helicases have been intensively studied in plant growth and development and response to various stresses [8], [10], [11], [12], [13], [16], [17], [19], [20], [24], [45], [46], [47], only a few members have been identified in the regulation of crop plant growth and development. While *Arabidopsis* and *Oryza sativa* RNA helicase families have been partially predicted from The *Arabidopsis* Information Resource (TAIR) database (http://www.arabidopsis.org/) and the Rice Genome Annotation Project (RAP) database (http://rice.plantbiology.msu.edu/) [2], [48], [49], the characteristics of the gene family and the function of RNA helicase proteins is poorly understood in *Zea mays* and *Glycine max*. Therefore, the biological functions of a majority of the crop RNA helicases require further investigation.

In this study, we presented a complete analysis of the RNA helicase gene family in *Arabidopsis* and other crop species genomes by genome-wide comparative in silico analysis, including the gene classification, chromosomal locations, phylogenetic tree and expression profiles in different tissues and development stages under normal growth conditions.

The RNA helicase gene family has 113 members in *Arabidopsis* and 115 members in rice [2]. In this study, we identified a total of 161 and 149 RNA helicase genes in *Arabidopsis* and rice, respectively. We speculate that this phenomenon may be due to continual updates of the TAIR and RAP database. In addition, we also identified a total of 136 RNA helicase genes in maize and 213 members in soybean. Compared with *Arabidopsis* (genome size 125 Mb) [50] and rice (genome size 480 Mb) [51], the size of the RNA helicase gene family is smaller in maize (genome size 2500 Mb) [52], [53] and soybean (genome size 1115 Mb) [54]. But compared with the number of all genes in four species genome (25,500 genes in *Arabidopsis* genome, 37,500 genes in rice genome, 50,000 genes in maize genome and 66,000 genes in soybean genome), the percentage of the RNA helicase gene family is similar in four species (0.631% in *Arabidopsis*, 0.397% in rice, 0.272% in maize and 0.323% in soybean), except higher in *Arabidopsis*. Although the genome and the total genes number in four species is very different, it still not show differ greatly in the size of RNA helicase gene family. The presence of a large helicase gene family in four species suggests the RNA helicases play important roles in diverse processes. We further compared the number of RNA helicase genes in different subfamilies among *Arabidopsis*, *Oryza sativa*, *Zea mays* and *Glycine max* (Table 1). As showed in Table 1, we founded that the number of the DEAD-box and DExD/H-box RNA helicase genes have many more members than the DEAH-box RNA helicase genes in the four species. The key difference is that the number of the alternative splicing products (35) was much smaller in *Glycine max* than in *Arabidopsis* (56), *Oryza sativa* (50), and *Zea mays* (79), rather than the number of different genes in each of the subfamilies.

Among the total RNA helicase genes in the four species, the percentage of alternative splicing is lowest in soybean (14.11%) and highest in maize (35.81%). The data showed that the phenomenon of alternative splicing of RNA helicase genes is more common in dicot than in monocot. The regulation of alternative splicing is a key step in the control of gene expression, as splicing variants have different biological functions and regulatory features. Alternative splicing is one of the most complex cellular processes in eukaryotes, where information must be processed differently at different times (such as different development stages) or a very high level of diversity is required. Only a small number of alternative splicing events have been reported in plants. Recent progress has occurred in characterising the splicing signals in plant pre-mRNAs, in identifying the mutants affected in splicing and in discovering new examples of alternatively spliced mRNAs. Furthermore, although data from both animals and plants suggest tissue-specific and temporal regulation of alternative splicing [55], [56], [57], [58], the mechanisms that regulate alternative splicing in plants remain unknown. Alternative splicing can result in the production of different protein isoforms, thereby affecting transcriptome and proteome diversity, and, ultimately, the regulation of protein function and gene expression [59], [60], [61]. Recent genome-wide experiments have shown that >40% of *Arabidopsis thaliana* and rice genes can produce multiple diverse mRNA molecules by alternative splicing [62], [63], [64], [65]. In our results, the percentage of alternative splicing of RNA helicase genes in *Arabidopsis thaliana* and rice (>25%) was less than the average alternative splicing frequency of whole genome. Our data concerning the alternative splicing frequency of RNA helicase genes in different crop species will not only provide information on mechanisms of gene regulation through alternative splicing in future but also facilitate our understanding of the regulation of RNA helicase genes in crop growth and development. To our knowledge, this is the first report of a genome-wide analysis of the crop RNA helicase gene family. The different gene subfamilies and alternative splicing frequency of the RNA helicases might mirror the diverse functions of these genes in RNA metabolism.

We also utilised a Genevestigator analysis to gain insight into the expression profiles of the RNA helicase genes during different development stages and in different tissues under normal growth conditions. We found that under normal growth conditions, among the all of the predicted genes in *Arabidopsis*, rice and maize, more than 80% RNA helicase genes were expressed in at least one of the development stages and tissues tested (Figure 4, 5, 6). In addition, about half of the predicted RNA helicase genes were expressed in at least one of the development stage and tissues tested in soybean (Figure 7). Therefore, we speculated that the highly expressed RNA helicase genes may play a role in the regulation of crop growth and development. However, more research will be needed to determine the functions of the RNA helicase genes in these four species. In addition, the results also showed that the percentage of different subfamilies in different development stage and tissues changed dissimilarly. The DEAH-box RNA helicase genes higher proportion of the development stages and tissues in *Arabidopsis*, *Oryza sativa* and *Zea mays* (Figure 4, 5, 6). Taken together, we speculate that the RNA helicase proteins, specifically the DEAH-box RNA helicases, might play an important role in different development stages of crops and the growth of different tissues.

To our knowledge, this is the first report of a comparative genome-wide analysis of the RNA helicase gene family in *Arabidopsis* and *Oryza sativa*, *Zea mays* and *Glycine max*. This study provides valuable information for understanding the classification and putative functions of the RNA helicase gene family in crop growth and development.

## Materials and Methods

### The Identification of the Helicase Genes in *Arabidopsis*, *Oryza Sativa*, *Zea Mays* and *Glycine Max*

To identify the members of the helicase gene family in *Arabidopsis*, *Oryza sativa*, *Zea mays* and *Glycine max*, two different approaches were performed [66]. First, the genome of the four species were downloaded from the database with different genome sizes, including *Arabidopsis* (genome size 125 Mb, 25,500 genes), rice (genome size 480 Mb, 37,500 genes), maize (genome size 2500 Mb, 50,000 genes) and soybean (genome size 1115 Mb, 66,000 genes) [51]–[54]. All known *Arabidopsis* helicase gene sequences, which were downloaded from the *Arabidopsis* genome TAIR 9.0 release (http://www.arabidopsis.org/), were used as query sequences to perform multiple database searches against the proteome and genome files downloaded from the Phytozome database (http://www.phytozome.net/). Stand-alone versions of BLASTP and TBLASTN (http://blast.ncbi.nlm.nih.gov), which are available from the NCBI, were used with an e-value cutoff set to 1e-003 [67]. All of the protein sequences derived from the collected candidate helicase genes were examined using the domain analysis programs PFAM (http://pfam.sanger.ac.uk/) and SMART (http://smart.embl-heidelberg. de/) with the default cutoff parameters [68], [69]. Second, we analysed the domains of all of the peptide sequences using a Hidden Markov Model (HMM) analysis with protein family (Pfam) searching (http://pfam.sbc.su.se/). Then, we obtained the sequences with the PF00271 Pfam number, which contained a typical helicase domain, from the genome sequences using a Perl-based script. Finally, all of the protein sequences were compared with known helicase sequences using ClustalX (http://www.clustal.org/) to verify the sequences were candidate helicases [70].

The isoelectric points and molecular weights of the proteins were obtained with the help of the proteomics and sequence analysis tools on the ExPASy Proteomics Server (http://expasy.org/) [71]. The chromosomal locations and the exon/intron information were obtained from the Phytozome database using a Perl-based program.

### The Chromosomal Location of the Helicase Genes

The chromosomal locations were retrieved from the genome data downloaded from the Phytozome database (http://www.phytozome.net/) using a Perl-based program and mapped to the chromosomes using the MapDraw and Photoshop tools.

### Sequence Alignment and Phylogenetic Analysis

The helicase sequences were aligned using the ClustalX program with BLOSUM30 as the protein-weight matrix. The MUSCLE program (version 3.52) was also used to perform multiple sequence alignments to confirm the ClustalX results (http://www.clustal.org/) [72]. Phylogenetic trees of the helicase protein sequences were constructed using the neighbour-joining (NJ) method of the MEGA5 program (http://www.megasoftware.net/) using the p-distance and complete deletion option parameters [73]. The reliability of the obtained trees was tested using a bootstrapping method with 1000 replicates. The images of the phylogenetic trees were drawn using MEGA5.

### Expression Analyses of the Helicase Genes in *Arabidopsis*, *Oryza Sativa*, *Zea Mays* and *Glycine Max*

Microarray expression data from various datasets were obtained using Genevestigator (https://www.genevestigator.com/gv/) with the *Arabidopsis* (ATH1∶22 k array), *Oryza sativa* (OS_51 k: Rice Genome 51 k array), *Zea mays* (ZM_84 k: Nimblegen Maize 385 k) and *Glycine max* (GM_60 k: Soybean Genome Array) Gene Chip platform. Then, the identified helicase-containing gene IDs were used as query sequences to perform searches in the Gene Chip platform of Genevestigator.

### Plant Materials and Growth Conditions

*Arabidopsis thaliana* (Col-0) seeds were surface-sterilized and sown on MS plates. Seeds were stratified at 4°C for 2 days and then transferred to 22°C for 2 weeks. One month-old plants grown under a 16-h-light/8-h-dark photoperiod at 22°C with cool white light (120 mmol photons m^−2^ s^−1^) were used for sampling. For RNA extraction, the different tissues were frozen and stored in liquid nitrogen immediately after harvest.

For maize inbred line Qi 319 (from Shandong Academy of Agricultural Sciences), embryos 25 days after pollination was harvested from greenhouse-grown plants in sand under 16 h of light (25°C) and 8 h of dark (20°C), and eight-week-old seedling tissues and organs were harvested for expression analysis. Samples were collected and were immediately frozen in liquid nitrogen for further use. Two biological replicates were performed for each sample.

### RNA Isolation and Real-time Quantitative RT-PCR Expression Analysis

Total RNAs were extracted using Trizol according to the manufacturer’s instructions (Invitrogen, Carlsbad, CA, USA) from leaves of maize seedlings with different treatments. The first strand cDNAs were synthesised using First Strand cDNA Synthesis kit (Fermentas, USA).

Real-time quantification RT-PCR reactions were performed in Bio-RAD MyiQ™ Real-time PCR Detection System (Bio-Rad, USA) using the TransStart Top Green qPCR SuperMix (TransGen, China) according to the manufacturer’s instructions. Each PCR reaction (20 µL containing 10 µL 2×real-time PCR Mix (containing SYBR Green I), 0.5 µL of each primer, and appropriately diluted cDNA. The thermal cycling conditions were 95°C for 30 s followed by 45 cycles of 95°C for 15 s, 55°C −60°C for 30 s, and 72°C for 15 s. The *Zmactin* gene was used as an internal reference for all the qRT-PCR analysis. Each treatment was repeated three times independently. Relative gene expression was calculated according to the delta-delta Ct method of the system. The primers used are described in Table S2.

## Supporting Information

Figure S1 **Phylogenetic tree analysis of RNA helicase in** ***Arabidopsis*** **.** From left to right are the DEAD-box, DEAH-box and DExD/H-box RNA helicase proteins, respectively. The scale bar represents 0.2, 0.2 and 0.2 substitutions per sequence position, respectively. Sister pairs of paralogous helicase genes were indicated by red shadow, which had very strong bootstrap support (>90%).
(TIF)

Figure S2 **Phylogenetic tree analysis of RNA helicase in** ***Oryza sativa*** **.** From left to right are the DEAD-box, DEAH-box and DExD/H-box RNA helicase proteins, respectively. The scale bar represents 0.2, 0.2 and 0.2 substitutions per sequence position, respectively. Sister pairs of paralogous helicase genes were indicated by red shadow, which had very strong bootstrap support (>90%).
(TIF)

Figure S3 **Phylogenetic tree analysis of RNA helicase in** ***Zea mays*** **.** From left to right are the DEAD-box, DEAH-box and DExD/H-box RNA helicase proteins, respectively. The scale bar represents 0.2, 0.2 and 0.2 substitutions per sequence position, respectively. Sister pairs of paralogous helicase genes were indicated by red shadow, which had very strong bootstrap support (>90%).
(TIF)

Figure S4 **Phylogenetic tree analysis of RNA helicase in** ***Glycine max*** **.** From left to right are the DEAD-box, DEAH-box and DExD/H-box RNA helicase proteins, respectively. The scale bar represents 0.2, 0.2 and 0.2 substitutions per sequence position, respectively. Sister pairs of paralogous helicase genes were indicated by red shadow, which had very strong bootstrap support (>90%).
(TIF)

Table S1 **RNA helicase genes in** ***Arabidopsis, Oryza sativa, Zea mays, Glycine max*** **.**
(DOC)

Table S2 **The primers used in the Real-time quantification RT-PCR reactions.**
(DOC)
