# Correction: Genome-Wide Comparative In Silico Analysis of the RNA Helicase Gene Family in *Zea mays* and *Glycine max*: A Comparison with *Arabidopsis* and *Oryza sativa*

**DOI:** 10.1371/journal.pone.0240759

**Published:** 2020-10-09

**Authors:** Ruirui Xu, Shizhong Zhang, Jinguang Huang, Chengchao Zheng

In the The Expression Profile During Different Development Stage and Different Tissues of the RNA Helicase Genes in Four Species Under Normal Growth Conditions subsection of the Results, there is an error in the first sentence of the third paragraph. The correct sentence is: Approximately 108 *Zea mays* genes were expressed with various expression levels in the tested development stages, including dough stage, fruit formation, anthesis, inflorescence formation, stem elongation, seedling stage and germination (Fig 6).

There are errors in the number of RNA expression profiles in the captions of Fig 4, Fig 5, Fig 6, and Fig 7. Please see the complete, correct captions here.

**Fig 4:**
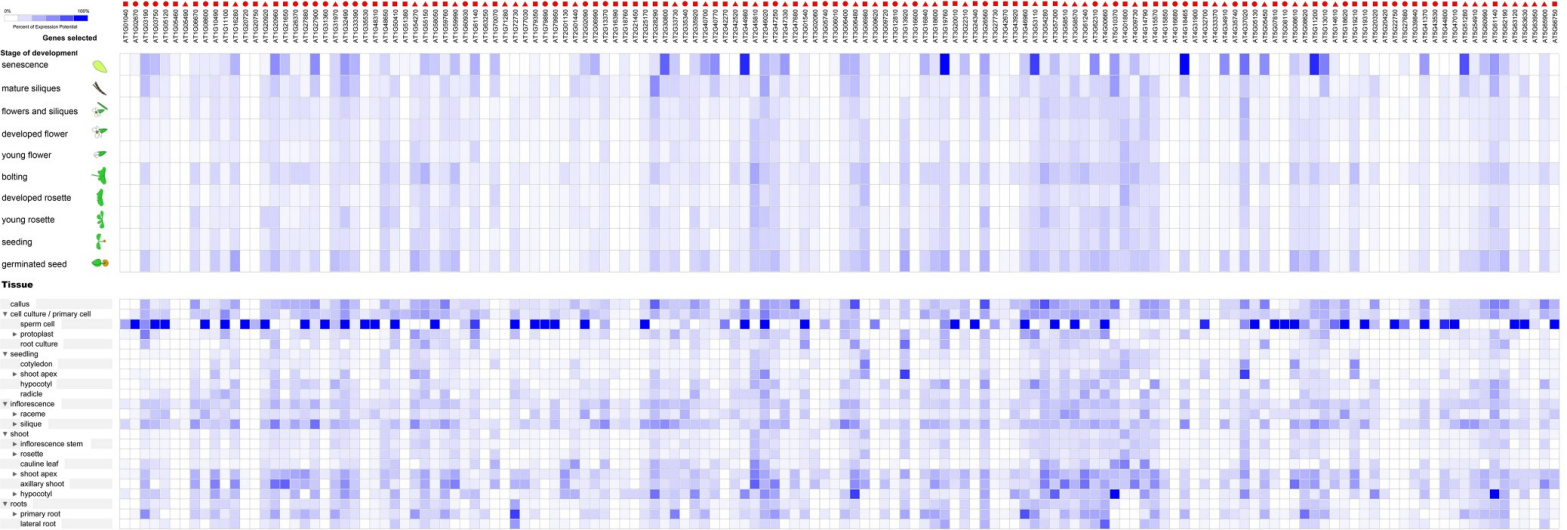
The expression profiles of 144 RNA helicase genes in *Arabidopsis*. The expression profiles of 144 RNA helicase genes in different development stages (above) and tissues (below) in *Arabidopsis*. The deep and light blue shading represents the relative high or low percent potential expression levels, respectively, of the helicase genes in different development stages and different tissues. The DEAD-box, DEAH-box and DExD/H-box RNA helicase genes are indicated by the red triangle, circle and square, respectively.

**Fig 5:**
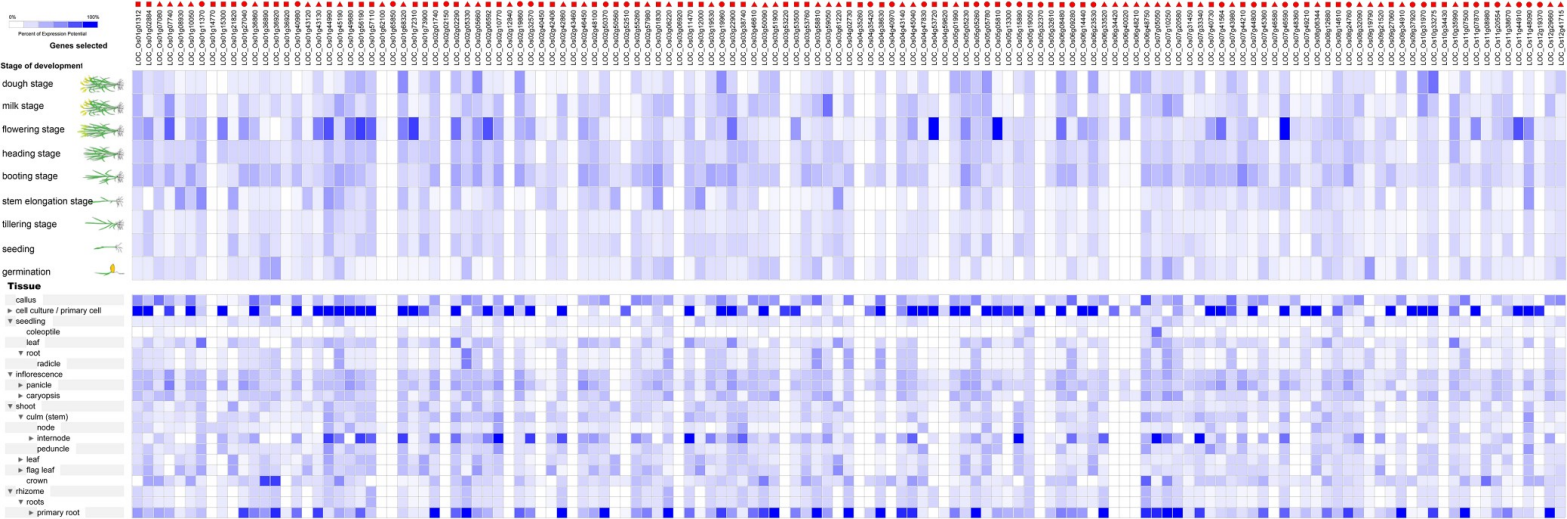
The expression profiles of 135 RNA helicase genes in *Oryza sativa*. The expression profiles of 135 RNA helicase genes in different development stages (above) and different tissues (below) in *Oryza sativa*. The deep and light blue shading represents the relative high or low percent potential expression levels, respectively, of the helicase genes in different development stages and different tissues. The DEAD-box, DEAH-box and DExD/H-box RNA helicase genes are indicated by the red triangle, circle and square, respectively.

**Fig 6:**
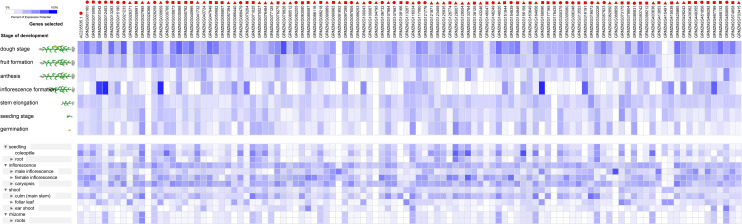
The expression profiles of 108 RNA helicase genes in *Zea mays*. The expression profiles of 108 RNA helicase genes in different development stages (above) and different tissues (below) in *Zea mays*. The deep and light blue shading represents the relative high or low percent potential expression levels, respectively, of the helicase genes in different development stages and different tissues. The DEAD-box, DEAH-box and DExD/H-box RNA helicase genes are indicated by the red triangle, circle and square, respectively.

**Fig 7:**
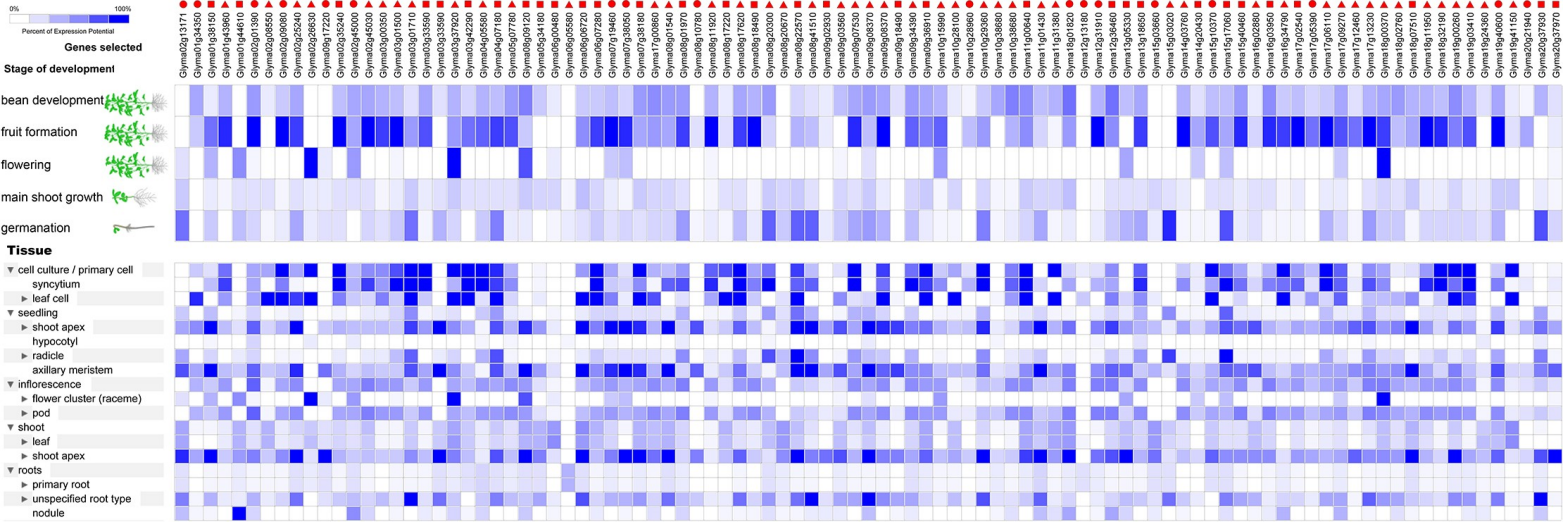
The expression profiles of 97 RNA helicase genes in *Glycine max*. The expression profiles of 97 RNA helicase genes in different development stages (above) and different tissues (below) in *Glycine max*. The deep and light blue shading represents the relative high or low percent potential expression levels, respectively, of the helicase genes in different development sages and different tissues. The DEAD-box, DEAH-box and DExD/H-box RNA helicase genes are indicated by the red triangle, circle and square, respectively.

The image for Fig 4 is incorrect. Please see the correct image here.
