# Supplementary figures and images for: Genome-Wide Comparative In Silico Analysis of the RNA Helicase Gene Family in Zea mays and Glycine max: A Comparison with Arabidopsis and Oryza sativa
Source: PLoS One. 2013 Nov 12;8(11):e78982. doi: 10.1371/journal.pone.0078982 (PMC3827086; doi:10.1371/journal.pone.0078982)

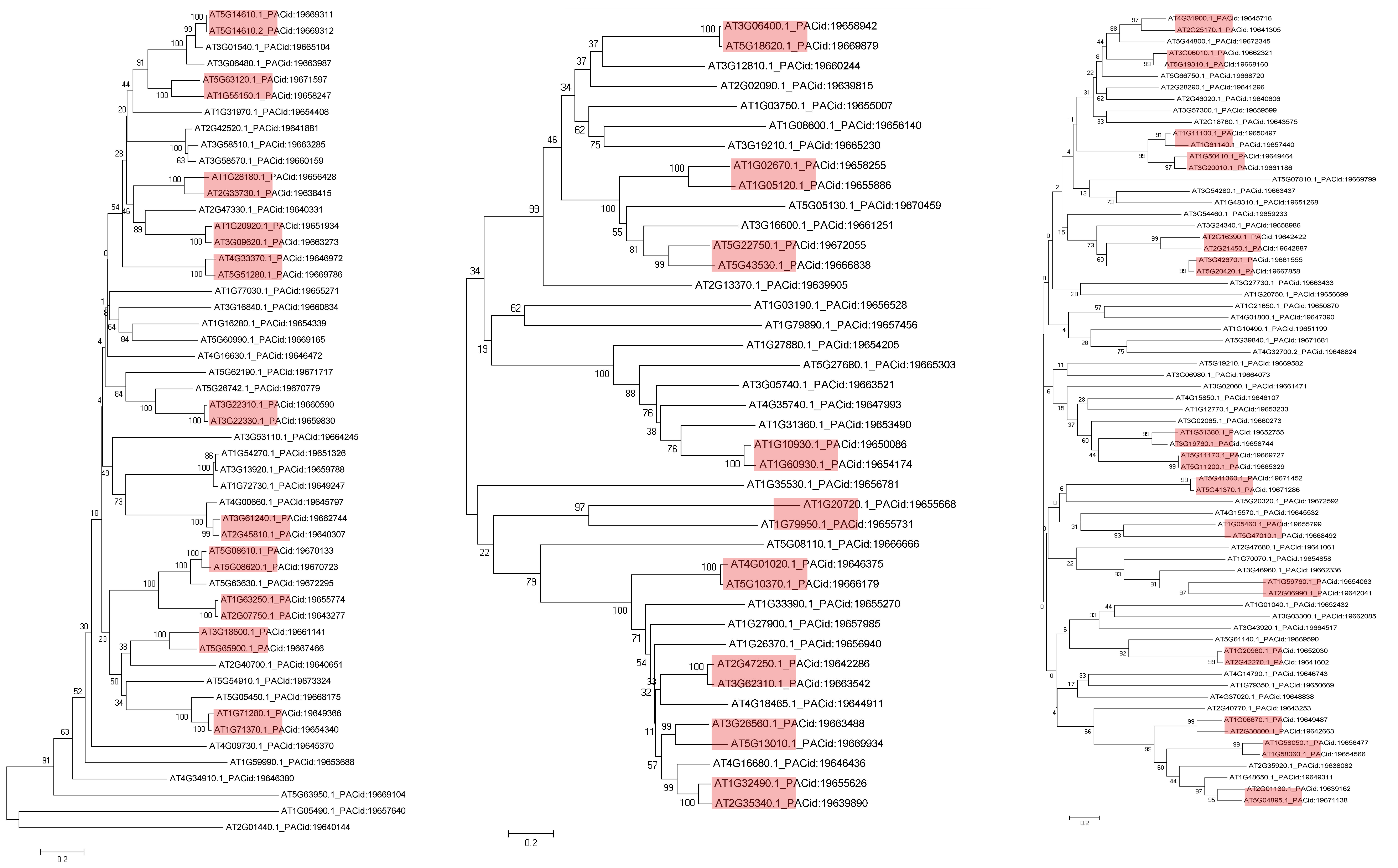

Supplement: Figure S1 — Phylogenetic tree analysis of RNA helicase in Arabidopsis . From left to right are the DEAD-box, DEAH-box and DExD/H-box RNA helicase proteins, respectively. The scale bar represents 0.2, 0.2 and 0.2 substitutions per sequence position, respectively. Sister pairs of paralogous helicase genes were indicated by red shadow, which had very strong bootstrap support (>90%). (TIF) [file pone.0078982.s001.tif]

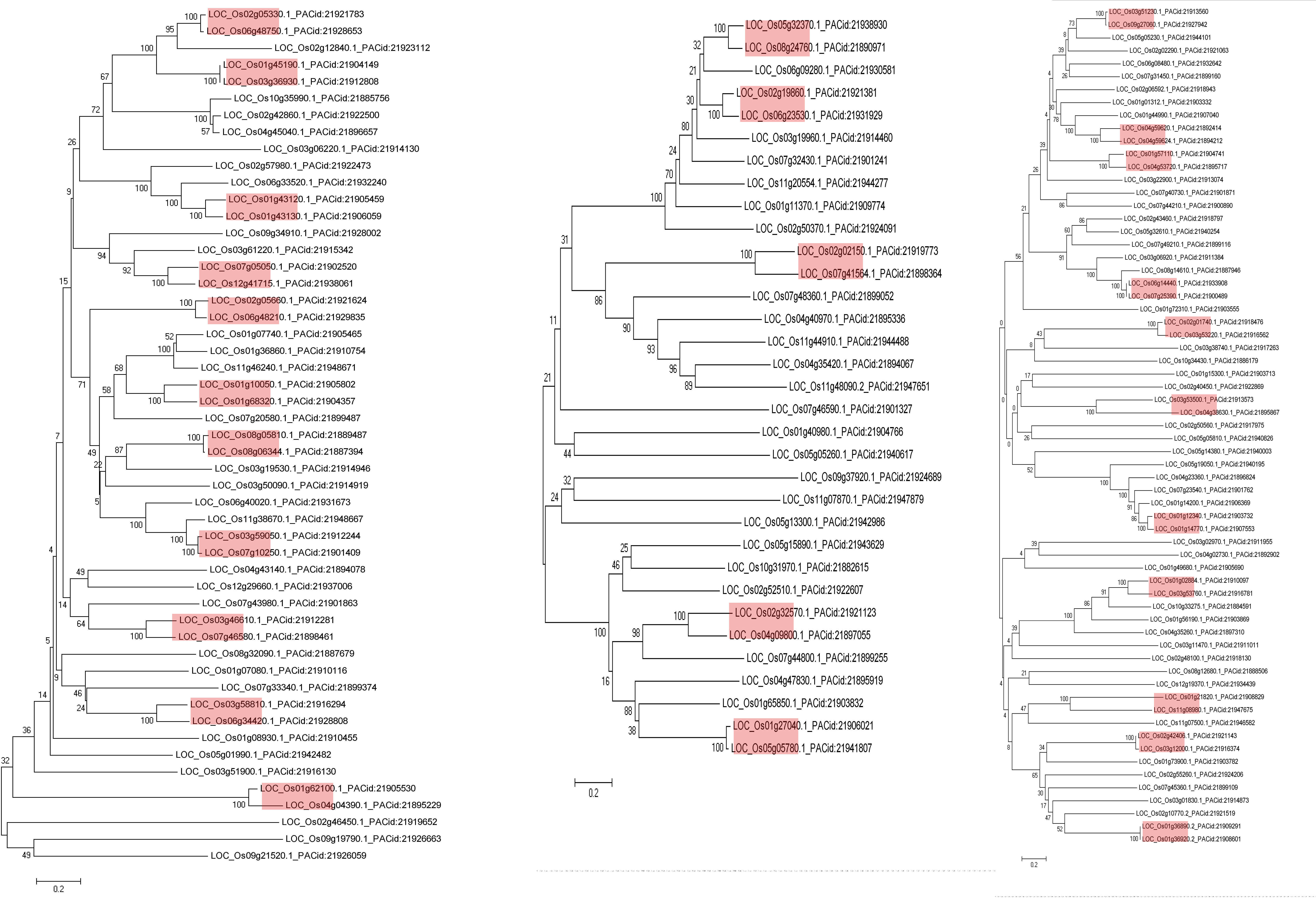

Supplement: Figure S2 — Phylogenetic tree analysis of RNA helicase in Oryza sativa . From left to right are the DEAD-box, DEAH-box and DExD/H-box RNA helicase proteins, respectively. The scale bar represents 0.2, 0.2 and 0.2 substitutions per sequence position, respectively. Sister pairs of paralogous helicase genes were indicated by red shadow, which had very strong bootstrap support (>90%). (TIF) [file pone.0078982.s002.tif]

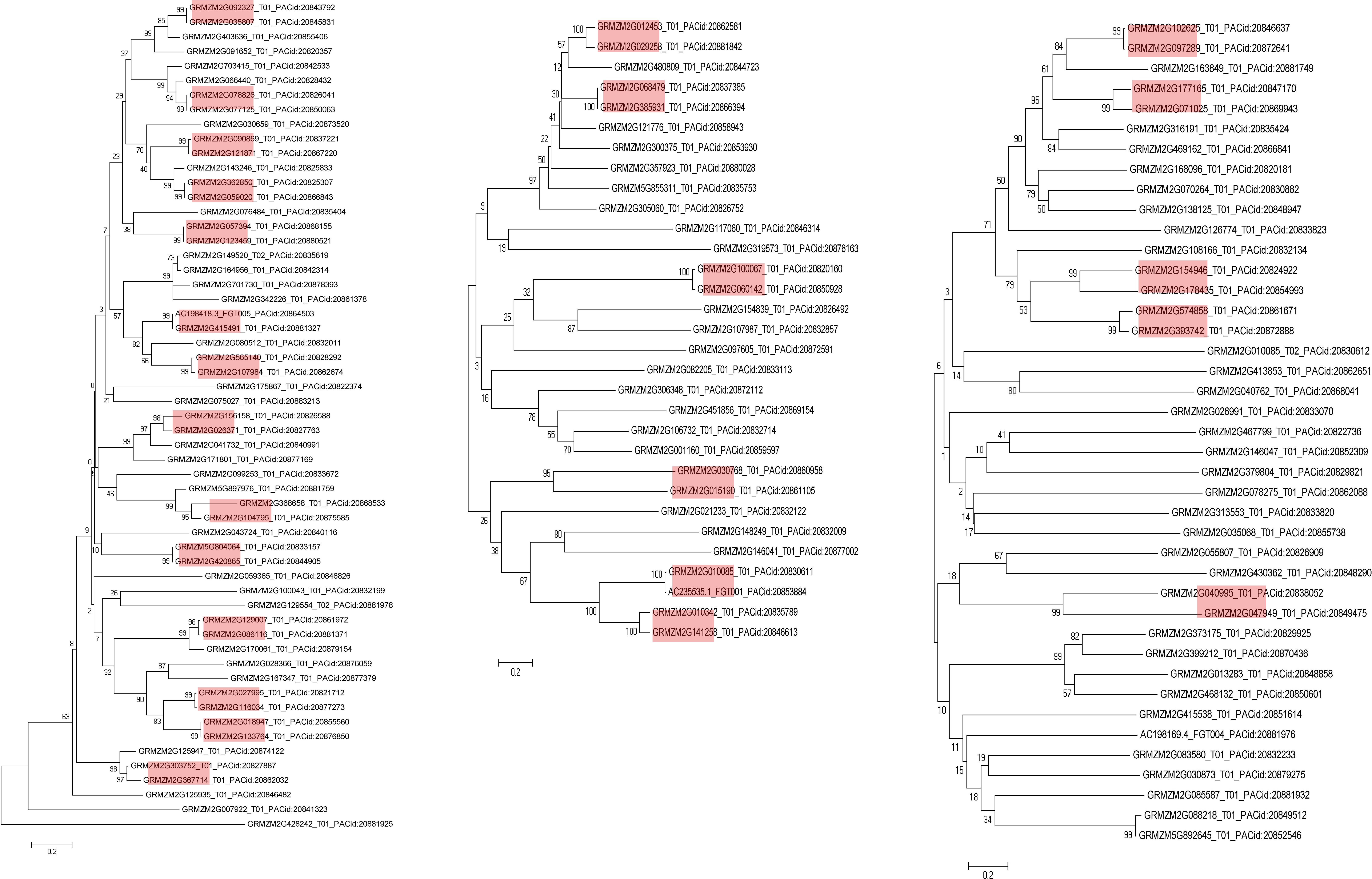

Supplement: Figure S3 — Phylogenetic tree analysis of RNA helicase in Zea mays . From left to right are the DEAD-box, DEAH-box and DExD/H-box RNA helicase proteins, respectively. The scale bar represents 0.2, 0.2 and 0.2 substitutions per sequence position, respectively. Sister pairs of paralogous helicase genes were indicated by red shadow, which had very strong bootstrap support (>90%). (TIF) [file pone.0078982.s003.tif]

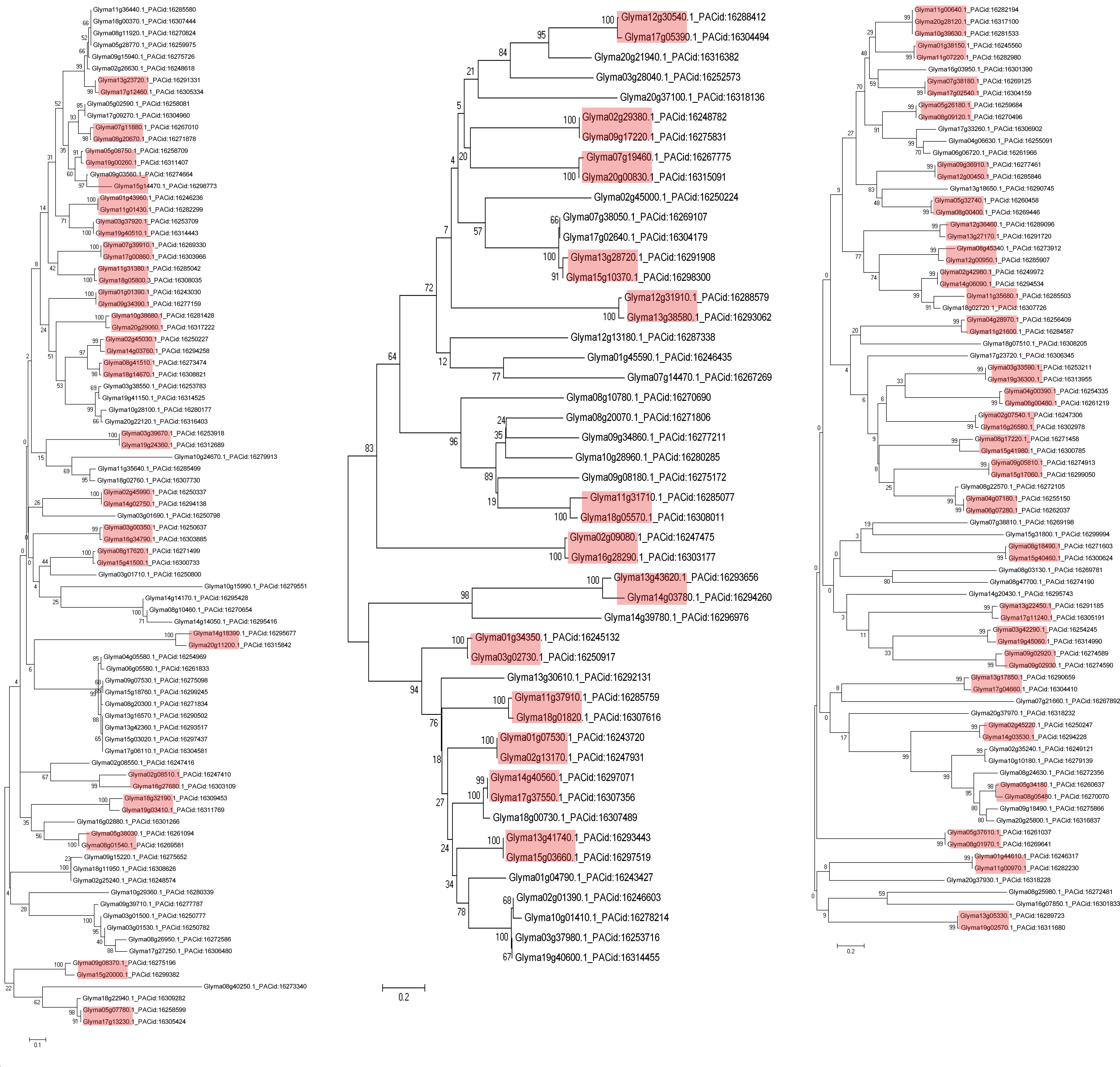

Supplement: Figure S4 — Phylogenetic tree analysis of RNA helicase in Glycine max . From left to right are the DEAD-box, DEAH-box and DExD/H-box RNA helicase proteins, respectively. The scale bar represents 0.2, 0.2 and 0.2 substitutions per sequence position, respectively. Sister pairs of paralogous helicase genes were indicated by red shadow, which had very strong bootstrap support (>90%). (TIF) [file pone.0078982.s004.tif]
