# Supplementary material for: Genome-Wide Comparative In Silico Analysis of the RNA Helicase Gene Family in Zea mays and Glycine max: A Comparison with Arabidopsis and Oryza sativa
Source: PLoS One. 2013 Nov 12;8(11):e78982. doi: 10.1371/journal.pone.0078982 (PMC3827086; doi:10.1371/journal.pone.0078982)
Supplement: Table S1 — RNA helicase genes in Arabidopsis, Oryza sativa, Zea mays, Glycine max . (DOC) [file pone.0078982.s005.doc]

Table S1 RNA helicase genes in *Arabidopsis*, *Oryza sativa*, *Zea mays*, *Glycine max*.

| Species | Helicase type | Gene ID | Protein Length (aa) | CDS | Mass (Da) | IP | Location |
| --- | --- | --- | --- | --- | --- | --- | --- |
| *Arabidopsis* | DEAD-box | AT1G16280.1 | 492 | 1479 | 54835.48 | 9.0 | Chr1:5568482..5570487 |
| *Arabidopsis* | DEAD-box | AT5G60990.1 | 457 | 1374 | 51147.02 | 9.07 | Chr5:24546430..24549339 |
| *Arabidopsis* | DEAD-box | AT4G16630.1 | 790 | 2373 | 89354.8 | 5.51 | Chr4:9361984..9366748 |
| *Arabidopsis* | DEAD-box | AT1G71280.1 | 466 | 1401 | 52435.74 | 9.27 | Chr1:26870141..26872152 |
| *Arabidopsis* | DEAD-box | AT1G71370.1 | 559 | 1680 | 62819.63 | 9.18 | Chr1:26897005..26899434 |
| *Arabidopsis* | DEAD-box | AT5G05450.1 | 594 | 1785 | 66939.19 | 8.88 | Chr5:1612006..1615356 |
| *Arabidopsis* | DEAD-box | AT3G18600.1 | 569 | 1710 | 63816.46 | 9.3 | Chr3:6399571..6403348 |
| *Arabidopsis* | DEAD-box | AT5G65900.1 | 634 | 1905 | 72052.5 | 8.86 | Chr5:26358206..26361443 |
| *Arabidopsis* | DEAD-box | AT2G40700.1 | 610 | 1833 | 68213.79 | 9.41 | Chr2:16976783..16979558 |
| *Arabidopsis* | DEAD-box | AT3G22310.1 | 611 | 1836 | 63609.38 | 9.44 | Chr3:7887286..7890019 |
| *Arabidopsis* | DEAD-box | AT3G22330.1 | 617 | 1854 | 65359.35 | 9.34 | Chr3:7892537..7895366 |
| *Arabidopsis* | DEAD-box | AT5G26742.1 | 748 | 2247 | 81000.22 | 7.24 | Chr5:9285082..9289003 |
| *Arabidopsis* | DEAD-box | AT5G26742.2 | 749 | 2250 | 81156.41 | 7.66 | Chr5:9284986..9289003 |
| *Arabidopsis* | DEAD-box | AT5G26742.3 | 656 | 1971 | 70893.28 | 9.06 | Chr5:9284979..9288727 |
| *Arabidopsis* | DEAD-box | AT5G62190.1 | 672 | 2019 | 72890.64 | 9.29 | Chr5:24980268..24983973 |
| *Arabidopsis* | DEAD-box | AT1G20920.1 | 1167 | 3504 | 133033.5 | 5.69 | Chr1:7285114..7289778 |
| *Arabidopsis* | DEAD-box | AT1G20920.2 | 829 | 2490 | 90938.22 | 5.29 | Chr1:7285059..7289778 |
| *Arabidopsis* | DEAD-box | AT3G09620.1 | 990 | 2973 | 111625.5 | 5.65 | Chr3:2949152..2952205 |
| *Arabidopsis* | DEAD-box | AT2G47330.1 | 761 | 2286 | 83050.62 | 5.61 | Chr2:19428897..19431720 |
| *Arabidopsis* | DEAD-box | AT5G63120.1 | 485 | 1458 | 53094.28 | 9.36 | Chr5:25318752..25322265 |
| *Arabidopsis* | DEAD-box | AT5G63120.2 | 592 | 1779 | 64621.23 | 9.66 | Chr5:25318752..25322265 |
| *Arabidopsis* | DEAD-box | AT1G55150.1 | 502 | 1509 | 55576.13 | 9.1 | Chr1:20574480..20577320 |
| *Arabidopsis* | DEAD-box | AT3G01540.1 | 619 | 1860 | 67640.52 | 9.77 | Chr3:212751..216671 |
| *Arabidopsis* | DEAD-box | AT3G01540.2 | 620 | 1863 | 67727.59 | 9.77 | Chr3:212751..216671 |
| *Arabidopsis* | DEAD-box | AT3G01540.3 | 620 | 1863 | 67727.59 | 9.77 | Chr3:212751..216666 |
| *Arabidopsis* | DEAD-box | AT3G01540.4 | 620 | 1863 | 67727.59 | 9.77 | Chr3:212518..216671 |
| *Arabidopsis* | DEAD-box | AT5G14610.1 | 713 | 2142 | 76257.06 | 9.45 | Chr5:4710563..4715069 |
| *Arabidopsis* | DEAD-box | AT5G14610.2 | 646 | 1941 | 69195.68 | 9.43 | Chr5:4710564..4715069 |
| *Arabidopsis* | DEAD-box | AT3G06480.1 | 1089 | 3270 | 119556.4 | 9.95 | Chr3:1985455..1990153 |
| *Arabidopsis* | DEAD-box | AT3G58510.1 | 613 | 1842 | 66025.66 | 6.23 | Chr3:21639978..21643795 |
| *Arabidopsis* | DEAD-box | AT3G58510.2 | 613 | 1842 | 66025.66 | 6.23 | Chr3:21640045..21643795 |
| *Arabidopsis* | DEAD-box | AT3G58510.3 | 613 | 1842 | 66025.66 | 6.23 | Chr3:21640010..21643795 |
| *Arabidopsis* | DEAD-box | AT2G42520.1 | 634 | 1905 | 67626.29 | 6.63 | Chr2:17704832..17708947 |
| *Arabidopsis* | DEAD-box | AT3G58570.1 | 647 | 1944 | 69242.07 | 7.66 | Chr3:21656504..21660532 |
| *Arabidopsis* | DEAD-box | AT1G31970.1 | 538 | 1617 | 59603.5 | 8.69 | Chr1:11479866..11482890 |
| *Arabidopsis* | DEAD-box | AT4G33370.1 | 543 | 1632 | 60258.24 | 6.59 | Chr4:16069669..16071405 |
| *Arabidopsis* | DEAD-box | AT5G51280.1 | 592 | 1779 | 65806.43 | 6.25 | Chr5:20841245..20843806 |
| *Arabidopsis* | DEAD-box | AT1G28180.1 | 615 | 1848 | 71719.01 | 8.78 | Chr1:9843075..9845002 |
| *Arabidopsis* | DEAD-box | AT2G33730.1 | 734 | 2205 | 85277.24 | 8.61 | Chr2:14265450..14267972 |
| *Arabidopsis* | DEAD-box | AT1G54270.1 | 413 | 1242 | 46762.55 | 5.45 | Chr1:20259692..20262266 |
| *Arabidopsis* | DEAD-box | AT1G54270.2 | 408 | 1227 | 46194.91 | 5.45 | Chr1:20259746..20262058 |
| *Arabidopsis* | DEAD-box | AT3G13920.1 | 413 | 1242 | 46704.49 | 5.47 | Chr3:4592256..4594962 |
| *Arabidopsis* | DEAD-box | AT3G13920.4 | 408 | 1227 | 46136.85 | 5.47 | Chr3:4592615..4594999 |
| *Arabidopsis* | DEAD-box | AT3G13920.3 | 403 | 1212 | 45700.55 | 5.9 | Chr3:4592305..4594919 |
| *Arabidopsis* | DEAD-box | AT3G13920.2 | 416 | 1251 | 46949.92 | 5.98 | Chr3:4592256..4594962 |
| *Arabidopsis* | DEAD-box | AT1G72730.1 | 415 | 1248 | 46770.52 | 5.16 | Chr1:27377798..27380182 |
| *Arabidopsis* | DEAD-box | AT3G61240.1 | 499 | 1500 | 56774.75 | 8.66 | Chr3:22666183..22669757 |
| *Arabidopsis* | DEAD-box | AT3G61240.2 | 499 | 1500 | 56774.75 | 8.66 | Chr3:22666183..22669496 |
| *Arabidopsis* | DEAD-box | AT2G45810.1 | 529 | 1590 | 60420.81 | 7.51 | Chr2:18859599..18862918 |
| *Arabidopsis* | DEAD-box | AT4G00660.1 | 506 | 1521 | 57686.87 | 8.4 | Chr4:274257..278737 |
| *Arabidopsis* | DEAD-box | AT4G00660.2 | 506 | 1521 | 57686.87 | 8.4 | Chr4:274257..278737 |
| *Arabidopsis* | DEAD-box | AT1G63250.1 | 799 | 2400 | 89590.67 | 9.36 | Chr1:23463069..23466451 |
| *Arabidopsis* | DEAD-box | AT2G07750.1 | 846 | 2541 | 94886.11 | 9.48 | Chr2:3576465..3580525 |
| *Arabidopsis* | DEAD-box | AT5G08610.1 | 851 | 2556 | 94186 | 9.5 | Chr5:2790295..2794215 |
| *Arabidopsis* | DEAD-box | AT5G08620.1 | 564 | 1695 | 62494.76 | 9.8 | Chr5:2794457..2797660 |
| *Arabidopsis* | DEAD-box | AT5G63630.1 | 789 | 2370 | 89626.57 | 9.02 | Chr5:25472598..25476511 |
| *Arabidopsis* | DEAD-box | AT1G05490.1 | 1411 | 4236 | 158723.4 | 5.76 | Chr1:1618602..1623452 |
| *Arabidopsis* | DEAD-box | AT3G53110.1 | 497 | 1494 | 55384.05 | 5.16 | Chr3:19687740..19690661 |
| *Arabidopsis* | DEAD-box | AT5G54910.1 | 740 | 2223 | 83581.56 | 9.15 | Chr5:22298557..22301719 |
| *Arabidopsis* | DEAD-box | AT1G77030.1 | 846 | 2541 | 93503.44 | 9.9 | Chr1:28947743..28951599 |
| *Arabidopsis* | DEAD-box | AT4G34910.1 | 627 | 1884 | 69501.75 | 8.86 | Chr4:16631533..16635149 |
| *Arabidopsis* | DEAD-box | AT3G16840.1 | 827 | 2484 | 93986.08 | 8.56 | Chr3:5737886..5743144 |
| *Arabidopsis* | DEAD-box | AT4G09730.1 | 622 | 1869 | 68923.9 | 9.91 | Chr4:6136249..6139711 |
| *Arabidopsis* | DEAD-box | AT1G59990.1 | 582 | 1749 | 64747.37 | 5.8 | Chr1:22090238..22093060 |
| *Arabidopsis* | DEAD-box | AT2G01440.1 | 974 | 2925 | 108376.2 | 7.06 | Chr2:193701..199248 |
| *Arabidopsis* | DEAD-box | AT5G63950.1 | 1091 | 3276 | 122802.6 | 5.99 | Chr5:25591956..25598505 |
| *Arabidopsis* | DEAH-box | AT1G35530.1 | 1325 | 3978 | 148354.9 | 5.95 | Chr1:13089869..13097078 |
| *Arabidopsis* | DEAH-box | AT1G35530.2 | 1391 | 4176 | 155795.5 | 6.29 | Chr1:13089869..13097078 |
| *Arabidopsis* | DEAH-box | AT1G10930.1 | 1189 | 3570 | 133312.5 | 6.82 | Chr1:3647894..3655590 |
| *Arabidopsis* | DEAH-box | AT1G60930.1 | 1151 | 3456 | 128577.5 | 8.54 | Chr1:22430847..22438877 |
| *Arabidopsis* | DEAH-box | AT1G31360.1 | 706 | 2121 | 79368.84 | 7.21 | Chr1:11232333..11237530 |
| *Arabidopsis* | DEAH-box | AT1G31360.2 | 581 | 1746 | 65651.41 | 8.48 | Chr1:11232333..11237530 |
| *Arabidopsis* | DEAH-box | AT3G05740.1 | 607 | 1824 | 68501.06 | 5.35 | Chr3:1698112..1701399 |
| *Arabidopsis* | DEAH-box | AT4G35740.1 | 714 | 2145 | 79944.17 | 8.37 | Chr4:16936120..16940332 |
| *Arabidopsis* | DEAH-box | AT4G35740.2 | 621 | 1866 | 69918.27 | 7.49 | Chr4:16936120..16940332 |
| *Arabidopsis* | DEAH-box | AT1G03190.1 | 759 | 2280 | 86235.52 | 7.23 | Chr1:775527..780027 |
| *Arabidopsis* | DEAH-box | AT1G03190.2 | 759 | 2280 | 86235.52 | 7.23 | Chr1:775622..780062 |
| *Arabidopsis* | DEAH-box | AT1G20720.1 | 1176 | 3531 | 131615.9 | 8.03 | Chr1:7185658..7192794 |
| *Arabidopsis* | DEAH-box | AT2G47250.1 | 730 | 2193 | 82653.66 | 6.94 | Chr2:19399694..19403044 |
| *Arabidopsis* | DEAH-box | AT3G62310.1 | 727 | 2184 | 82693.87 | 6.94 | Chr3:23057245..23060731 |
| *Arabidopsis* | DEAH-box | AT3G26560.1 | 1169 | 3510 | 134157.4 | 6.4 | Chr3:9749904..9753772 |
| *Arabidopsis* | DEAH-box | AT4G18465.1 | 696 | 2091 | 77715.08 | 8.01 | Chr4:10197056..10201611 |
| *Arabidopsis* | DEAH-box | AT1G26370.1 | 718 | 2157 | 80515.39 | 8.79 | Chr1:9121492..9125557 |
| *Arabidopsis* | DEAH-box | AT1G32490.1 | 1045 | 3138 | 118831.8 | 5.78 | Chr1:11742054..11749775 |
| *Arabidopsis* | DEAH-box | AT1G32490.2 | 1035 | 3108 | 117580.5 | 5.86 | Chr1:11742060..11749788 |
| *Arabidopsis* | DEAH-box | AT2G35340.1 | 1045 | 3138 | 119127.3 | 5.84 | Chr2:14872320..14879739 |
| *Arabidopsis* | DEAH-box | AT4G16680.1 | 657 | 1974 | 74643.58 | 8.27 | Chr4:9387393..9390774 |
| *Arabidopsis* | DEAH-box | AT1G27900.1 | 701 | 2106 | 78369.87 | 8.18 | Chr1:9715453..9720568 |
| *Arabidopsis* | DEAH-box | AT5G13010.1 | 1256 | 3771 | 141859.8 | 6.23 | Chr5:4122410..4128861 |
| *Arabidopsis* | DEAH-box | AT4G01020.1 | 1788 | 5367 | 202131 | 6.58 | Chr4:439086..445168 |
| *Arabidopsis* | DEAH-box | AT5G10370.1 | 1776 | 5331 | 201362.2 | 7.53 | Chr5:3261245..3267344 |
| *Arabidopsis* | DEAH-box | AT1G02670.1 | 679 | 2040 | 75759.18 | 5.86 | Chr1:576046..580299 |
| *Arabidopsis* | DEAH-box | AT1G05120.1 | 834 | 2505 | 94588.34 | 8.36 | Chr1:1471406..1476078 |
| *Arabidopsis* | DEAH-box | AT5G22750.1 | 1030 | 3093 | 114286.5 | 6.4 | Chr5:7565037..7571083 |
| *Arabidopsis* | DEAH-box | AT5G43530.1 | 1278 | 3837 | 144332.9 | 5.13 | Chr5:17489327..17494830 |
| *Arabidopsis* | DEAH-box | AT3G16600.1 | 639 | 1920 | 72995.04 | 9.37 | Chr3:5652839..5655670 |
| *Arabidopsis* | DEAH-box | AT1G08600.2 | 1480 | 4443 | 168175.9 | 5.36 | Chr1:2723824..2733613 |
| *Arabidopsis* | DEAH-box | AT1G08600.3 | 1480 | 4443 | 168175.9 | 5.36 | Chr1:2723824..2733613 |
| *Arabidopsis* | DEAH-box | AT1G08600.4 | 1480 | 4443 | 168175.9 | 5.36 | Chr1:2723824..2733613 |
| *Arabidopsis* | DEAH-box | AT1G08600.1 | 1459 | 4380 | 165915.2 | 5.27 | Chr1:2723824..2733613 |
| *Arabidopsis* | DEAH-box | AT3G19210.1 | 911 | 2736 | 101857.9 | 5.74 | Chr3:6652745..6658938 |
| *Arabidopsis* | DEAH-box | AT3G19210.2 | 909 | 2730 | 101580.7 | 5.66 | Chr3:6652778..6658988 |
| *Arabidopsis* | DEAH-box | AT3G06400.1 | 1056 | 3171 | 122428.5 | 5.69 | Chr3:1940992..1946924 |
| *Arabidopsis* | DEAH-box | AT3G06400.3 | 1058 | 3177 | 122613.7 | 5.73 | Chr3:1940973..1946885 |
| *Arabidopsis* | DEAH-box | AT3G06400.2 | 1057 | 3174 | 122557.6 | 5.65 | Chr3:1940973..1946885 |
| *Arabidopsis* | DEAH-box | AT5G18620.1 | 1070 | 3213 | 123888.4 | 5.7 | Chr5:6195926..6202166 |
| *Arabidopsis* | DEAH-box | AT5G18620.2 | 1073 | 3222 | 124161.7 | 5.7 | Chr5:6195917..6202166 |
| *Arabidopsis* | DEAH-box | AT2G13370.1 | 1725 | 5178 | 197270.1 | 5.5 | Chr2:5544181..5556464 |
| *Arabidopsis* | DEAH-box | AT1G33390.1 | 1238 | 3717 | 139145.2 | 5.78 | Chr1:12099482..12104601 |
| *Arabidopsis* | DEAH-box | AT3G12810.1 | 2056 | 6171 | 234048 | 5.21 | Chr3:4065042..4074078 |
| *Arabidopsis* | DEAH-box | AT2G02090.1 | 764 | 2295 | 86277.01 | 5.52 | Chr2:523284..527012 |
| *Arabidopsis* | DEAH-box | AT5G05130.1 | 863 | 2592 | 96345.76 | 8.61 | Chr5:1512120..1515079 |
| *Arabidopsis* | DEAH-box | AT5G27680.1 | 859 | 2580 | 97465.84 | 7.06 | Chr5:9793790..9798961 |
| *Arabidopsis* | DEAH-box | AT1G03750.1 | 863 | 2592 | 98486.4 | 6.53 | Chr1:937920..941162 |
| *Arabidopsis* | DEAH-box | AT1G79890.1 | 883 | 2652 | 99026.59 | 7.79 | Chr1:30048655..30052203 |
| *Arabidopsis* | DEAH-box | AT1G79950.1 | 1041 | 3126 | 116631.6 | 8.99 | Chr1:30073580..30079537 |
| *Arabidopsis* | DEAH-box | AT1G27880.1 | 912 | 2739 | 100754.9 | 8.94 | Chr1:9708924..9714089 |
| *Arabidopsis* | DEAH-box | AT5G08110.1 | 1142 | 3429 | 126459.7 | 8.57 | Chr5:2594722..2600114 |
| *Arabidopsis* | DExD/H-box | AT1G01040.1 | 1910 | 5733 | 213573.6 | 5.88 | Chr1:23146..31227 |
| *Arabidopsis* | DExD/H-box | AT1G01040.2 | 1911 | 5736 | 213672.8 | 5.88 | Chr1:23416..31120 |
| *Arabidopsis* | DExD/H-box | AT3G03300.1 | 1389 | 4170 | 156864.6 | 6.32 | Chr3:767926..776214 |
| *Arabidopsis* | DExD/H-box | AT3G03300.2 | 1375 | 4128 | 155285.7 | 6.46 | Chr3:768020..774525 |
| *Arabidopsis* | DExD/H-box | AT3G03300.3 | 1389 | 4170 | 156864.6 | 6.32 | Chr3:767926..775248 |
| *Arabidopsis* | DExD/H-box | AT3G43920.1 | 1532 | 4599 | 172013.6 | 5.9 | Chr3:15753548..15760830 |
| *Arabidopsis* | DExD/H-box | AT3G43920.2 | 1581 | 4746 | 177424.8 | 5.98 | Chr3:15753548..15760830 |
| *Arabidopsis* | DExD/H-box | AT3G43920.3 | 1571 | 4716 | 176448.8 | 5.98 | Chr3:15753548..15760859 |
| *Arabidopsis* | DExD/H-box | AT5G20320.1 | 1703 | 5112 | 191278.8 | 6.3 | Chr5:6859218..6869266 |
| *Arabidopsis* | DExD/H-box | AT5G20320.2 | 1689 | 5070 | 189677.8 | 6.29 | Chr5:6859229..6869307 |
| *Arabidopsis* | DExD/H-box | AT1G10490.1 | 1029 | 3090 | 115619.2 | 6.97 | Chr1:3453228..3460620 |
| *Arabidopsis* | DExD/H-box | AT1G21650.1 | 1052 | 3159 | 118931.9 | 6.09 | Chr1:7592664..7600590 |
| *Arabidopsis* | DExD/H-box | AT1G21650.2 | 1059 | 3180 | 119653.7 | 6.03 | Chr1:7592854..7600639 |
| *Arabidopsis* | DExD/H-box | AT1G21650.3 | 1806 | 5421 | 203110.1 | 5.45 | Chr1:7592854..7604229 |
| *Arabidopsis* | DExD/H-box | AT4G01800.1 | 1023 | 3072 | 115183.3 | 6.07 | Chr4:770722..776131 |
| *Arabidopsis* | DExD/H-box | AT4G01800.2 | 1043 | 3132 | 117414.2 | 6.29 | Chr4:770838..776260 |
| *Arabidopsis* | DExD/H-box | AT4G37020.1 | 213 | 642 | 23410.87 | 4.76 | Chr4:17445697..17447400 |
| *Arabidopsis* | DExD/H-box | AT4G37020.2 | 259 | 780 | 29019.3 | 5.85 | Chr4:17445710..17447369 |
| *Arabidopsis* | DExD/H-box | AT3G02065.2 | 506 | 1521 | 55217.97 | 8.53 | Chr3:358916..360889 |
| *Arabidopsis* | DExD/H-box | AT3G02065.3 | 506 | 1521 | 55217.97 | 8.53 | Chr3:359030..360889 |
| *Arabidopsis* | DExD/H-box | AT3G02065.1 | 369 | 1110 | 40126.88 | 9.48 | Chr3:358951..360889 |
| *Arabidopsis* | DExD/H-box | AT1G51380.1 | 393 | 1182 | 44357.1 | 8.11 | Chr1:19047882..19050162 |
| *Arabidopsis* | DExD/H-box | AT3G19760.1 | 409 | 1230 | 45844.53 | 5.85 | Chr3:6863713..6866593 |
| *Arabidopsis* | DExD/H-box | AT5G11170.1 | 428 | 1287 | 48337.45 | 5.42 | Chr5:3553117..3556977 |
| *Arabidopsis* | DExD/H-box | AT5G11170.2 | 345 | 1038 | 39093.29 | 6.38 | Chr5:3554183..3556960 |
| *Arabidopsis* | DExD/H-box | AT5G11200.1 | 428 | 1287 | 48337.45 | 5.42 | Chr5:3567174..3571015 |
| *Arabidopsis* | DExD/H-box | AT5G11200.2 | 487 | 1464 | 55464.77 | 5.61 | Chr5:3567175..3571015 |
| *Arabidopsis* | DExD/H-box | AT5G11200.3 | 469 | 1410 | 52859.64 | 5.61 | Chr5:3567175..3571015 |
| *Arabidopsis* | DExD/H-box | AT5G19210.1 | 316 | 951 | 35457.3 | 8.37 | Chr5:6461391..6463866 |
| *Arabidopsis* | DExD/H-box | AT5G19210.2 | 473 | 1422 | 52811.34 | 8.6 | Chr5:6461423..6463866 |
| *Arabidopsis* | DExD/H-box | AT1G20960.1 | 2172 | 6519 | 247104.8 | 5.5 | Chr1:7301748..7310250 |
| *Arabidopsis* | DExD/H-box | AT1G20960.2 | 2172 | 6519 | 247104.8 | 5.5 | Chr1:7301748..7310250 |
| *Arabidopsis* | DExD/H-box | AT2G42270.1 | 2173 | 6522 | 247389.3 | 5.65 | Chr2:17604330..17611333 |
| *Arabidopsis* | DExD/H-box | AT5G61140.1 | 2147 | 6444 | 243177.1 | 6.39 | Chr5:24589999..24603514 |
| *Arabidopsis* | DExD/H-box | AT5G61140.2 | 2158 | 6477 | 244527.7 | 6.42 | Chr5:24589906..24603514 |
| *Arabidopsis* | DExD/H-box | AT4G14790.1 | 572 | 1719 | 63642.14 | 8.18 | Chr4:8496031..8500152 |
| *Arabidopsis* | DExD/H-box | AT5G39840.1 | 777 | 2334 | 88194.42 | 5.62 | Chr5:15946769..15949186 |
| *Arabidopsis* | DExD/H-box | AT1G59760.1 | 989 | 2970 | 111887.1 | 5.83 | Chr1:21984445..21990175 |
| *Arabidopsis* | DExD/H-box | AT2G06990.1 | 996 | 2991 | 111889.2 | 6.02 | Chr2:2894940..2901078 |
| *Arabidopsis* | DExD/H-box | AT3G46960.1 | 1348 | 4047 | 151183.5 | 5.79 | Chr3:17290873..17298067 |
| *Arabidopsis* | DExD/H-box | AT1G06670.1 | 1577 | 4734 | 174509.9 | 6.37 | Chr1:2040433..2047612 |
| *Arabidopsis* | DExD/H-box | AT2G30800.1 | 1300 | 3903 | 144478.9 | 8.25 | Chr2:13120353..13126770 |
| *Arabidopsis* | DExD/H-box | AT1G48650.1 | 1198 | 3597 | 134253.1 | 9.1 | Chr1:17989398..17995326 |
| *Arabidopsis* | DExD/H-box | AT1G48650.2 | 1207 | 3624 | 135431.6 | 9.13 | Chr1:17989398..17995326 |
| *Arabidopsis* | DExD/H-box | AT2G01130.1 | 1114 | 3345 | 125749.1 | 7.7 | Chr2:88559..94660 |
| *Arabidopsis* | DExD/H-box | AT5G04895.1 | 1162 | 3489 | 130831.1 | 8.63 | Chr5:1428724..1434769 |
| *Arabidopsis* | DExD/H-box | AT2G35920.1 | 996 | 2991 | 111038.7 | 8.28 | Chr2:15075597..15080812 |
| *Arabidopsis* | DExD/H-box | AT1G58050.1 | 1418 | 4257 | 159338.7 | 7.37 | Chr1:21478590..21487839 |
| *Arabidopsis* | DExD/H-box | AT1G58060.1 | 1460 | 4383 | 163599.7 | 5.85 | Chr1:21489285..21501811 |
| *Arabidopsis* | DExD/H-box | AT1G11100.1 | 1227 | 3684 | 135675.5 | 5.16 | Chr1:3703691..3709302 |
| *Arabidopsis* | DExD/H-box | AT1G11100.2 | 1270 | 3813 | 140389.8 | 5.25 | Chr1:3703672..3709302 |
| *Arabidopsis* | DExD/H-box | AT1G61140.1 | 1281 | 3846 | 141128.7 | 5.04 | Chr1:22534717..22540850 |
| *Arabidopsis* | DExD/H-box | AT1G61140.3 | 1123 | 3372 | 124164.4 | 5.69 | Chr1:22534717..22540850 |
| *Arabidopsis* | DExD/H-box | AT1G61140.2 | 1023 | 3072 | 112923.7 | 5 | Chr1:22536215..22540610 |
| *Arabidopsis* | DExD/H-box | AT1G50410.1 | 982 | 2949 | 108110.6 | 6.84 | Chr1:18672163..18677556 |
| *Arabidopsis* | DExD/H-box | AT3G20010.1 | 1048 | 3147 | 115474.8 | 8.3 | Chr3:6971352..6976595 |
| *Arabidopsis* | DExD/H-box | AT3G57300.1 | 1508 | 4527 | 172171.7 | 8.97 | Chr3:21199488..21207885 |
| *Arabidopsis* | DExD/H-box | AT3G57300.2 | 1541 | 4626 | 175960.2 | 8.93 | Chr3:21199488..21207885 |
| *Arabidopsis* | DExD/H-box | AT5G66750.1 | 765 | 2298 | 86637.81 | 6.01 | Chr5:26648951..26653073 |
| *Arabidopsis* | DExD/H-box | AT4G31900.1 | 1203 | 3612 | 139416.6 | 5.35 | Chr4:15431528..15438443 |
| *Arabidopsis* | DExD/H-box | AT4G31900.2 | 1162 | 3489 | 134636.3 | 5.17 | Chr4:15431350..15438678 |
| *Arabidopsis* | DExD/H-box | AT2G25170.1 | 1385 | 4158 | 158405.3 | 5.42 | Chr2:10713807..10723986 |
| *Arabidopsis* | DExD/H-box | AT2G28290.2 | 3530 | 10593 | 384798.7 | 4.77 | Chr2:12056213..12073083 |
| *Arabidopsis* | DExD/H-box | AT2G28290.3 | 3544 | 10635 | 386412.4 | 4.76 | Chr2:12056213..12073083 |
| *Arabidopsis* | DExD/H-box | AT2G28290.1 | 3575 | 10728 | 389864.3 | 4.75 | Chr2:12056213..12073083 |
| *Arabidopsis* | DExD/H-box | AT3G06010.1 | 1103 | 3312 | 126734.7 | 6.17 | Chr3:1802228..1807478 |
| *Arabidopsis* | DExD/H-box | AT5G19310.1 | 1065 | 3198 | 122886.5 | 6.48 | Chr5:6498779..6503432 |
| *Arabidopsis* | DExD/H-box | AT2G46020.1 | 2193 | 6582 | 245439 | 8.98 | Chr2:18923304..18931769 |
| *Arabidopsis* | DExD/H-box | AT2G46020.2 | 2194 | 6585 | 245469.1 | 8.95 | Chr2:18923304..18931934 |
| *Arabidopsis* | DExD/H-box | AT3G54280.1 | 2046 | 6141 | 227055.3 | 5.55 | Chr3:20091976..20104155 |
| *Arabidopsis* | DExD/H-box | AT3G54280.2 | 2130 | 6393 | 236590.4 | 5.52 | Chr3:20091976..20104257 |
| *Arabidopsis* | DExD/H-box | AT3G24340.1 | 1133 | 3402 | 128343.3 | 5.04 | Chr3:8832085..8835722 |
| *Arabidopsis* | DExD/H-box | AT2G16390.1 | 889 | 2670 | 100229.4 | 5.24 | Chr2:7097280..7101261 |
| *Arabidopsis* | DExD/H-box | AT2G21450.1 | 817 | 2454 | 93297.72 | 6.94 | Chr2:9179410..9182356 |
| *Arabidopsis* | DExD/H-box | AT3G42670.1 | 1257 | 3774 | 144573.9 | 6.47 | Chr3:14755798..14760085 |
| *Arabidopsis* | DExD/H-box | AT5G20420.1 | 1262 | 3789 | 145313.6 | 7.13 | Chr5:6899015..6904167 |
| *Arabidopsis* | DExD/H-box | AT5G41360.1 | 767 | 2304 | 86735.92 | 8.22 | Chr5:16544340..16549280 |
| *Arabidopsis* | DExD/H-box | AT5G41370.1 | 768 | 2307 | 87062.36 | 8.33 | Chr5:16551119..16556003 |
| *Arabidopsis* | DExD/H-box | AT5G44800.1 | 2224 | 6675 | 247918.9 | 5.93 | Chr5:18083419..18093452 |
| *Arabidopsis* | DExD/H-box | AT3G09720.1 | 542 | 1629 | 60916.14 | 9.45 | Chr3:2980221..2983618 |
| *Arabidopsis* | DExD/H-box | AT4G15850.1 | 523 | 1572 | 57715.76 | 9.2 | Chr4:9001330..9004734 |
| *Arabidopsis* | DExD/H-box | AT3G06980.1 | 782 | 2349 | 87428.61 | 7.56 | Chr3:2201467..2204833 |
| *Arabidopsis* | DExD/H-box | AT2G18760.1 | 1188 | 3567 | 133592.6 | 6.76 | Chr2:8129154..8133502 |
| *Arabidopsis* | DExD/H-box | AT1G70070.1 | 1172 | 3519 | 132436.7 | 5.27 | Chr1:26390016..26394196 |
| *Arabidopsis* | DExD/H-box | AT3G27730.1 | 1134 | 3405 | 128289.6 | 8.14 | Chr3:10273801..10280362 |
| *Arabidopsis* | DExD/H-box | AT2G40770.1 | 1665 | 4998 | 188072.4 | 6.17 | Chr2:17013269..17021315 |
| *Arabidopsis* | DExD/H-box | AT1G12770.1 | 552 | 1659 | 60718.2 | 9.3 | Chr1:4351064..4353685 |
| *Arabidopsis* | DExD/H-box | AT1G48310.1 | 674 | 2025 | 75073.55 | 7.99 | Chr1:17848421..17853807 |
| *Arabidopsis* | DExD/H-box | AT5G07810.1 | 1191 | 3576 | 134318.9 | 6.42 | Chr5:2491283..2498484 |
| *Arabidopsis* | DExD/H-box | AT2G47680.1 | 1016 | 3051 | 115084.6 | 6.19 | Chr2:19545828..19550905 |
| *Arabidopsis* | DExD/H-box | AT3G54460.1 | 1379 | 4140 | 155413.4 | 7.03 | Chr3:20161942..20167293 |
| *Arabidopsis* | DExD/H-box | AT4G32700.2 | 2155 | 6468 | 238523.3 | 8.15 | Chr4:15767317..15779697 |
| *Arabidopsis* | DExD/H-box | AT1G79350.1 | 1296 | 3891 | 143617.2 | 7.1 | Chr1:29844633..29853414 |
| *Arabidopsis* | DExD/H-box | AT1G05460.1 | 1003 | 3012 | 113363 | 4.96 | Chr1:1601148..1605027 |
| *Arabidopsis* | DExD/H-box | AT1G20750.1 | 1145 | 3438 | 127980.9 | 6.78 | Chr1:7203302..7208998 |
| *Arabidopsis* | DExD/H-box | AT2G44980.2 | 878 | 2637 | 98867.47 | 5.6 | Chr2:18552192..18556669 |
| *Arabidopsis* | DExD/H-box | AT3G02060.1 | 824 | 2475 | 93929.14 | 8.9 | Chr3:354360..358807 |
| *Arabidopsis* | DExD/H-box | AT4G15570.1 | 819 | 2460 | 91539.74 | 5.53 | Chr4:8892607..8898999 |
| *Arabidopsis* | DExD/H-box | AT5G47010.1 | 1255 | 3768 | 136868.3 | 6.04 | Chr5:19072009..19079334 |
| *Oryza sativa* | DEAD-box | LOC_Os01g07080.1 | 648 | 1947 | 72217.43 | 9.45 | Chr1:3328533:3331963 |
| *Oryza sativa* | DEAD-box | LOC_Os01g07080.2 | 510 | 1533 | 55996.05 | 8.91 | Chr1:3329377:3331963 |
| *Oryza sativa* | DEAD-box | LOC_Os01g07740.1 | 760 | 2283 | 83300.39 | 9.29 | Chr1:3713687:3718637 |
| *Oryza sativa* | DEAD-box | LOC_Os01g07740.2 | 759 | 2280 | 83213.31 | 9.29 | Chr1:3713702:3718631 |
| *Oryza sativa* | DEAD-box | LOC_Os01g07740.3 | 709 | 2130 | 77297.35 | 9.21 | Chr1:3713687:3718637 |
| *Oryza sativa* | DEAD-box | LOC_Os01g08930.1 | 626 | 1881 | 67860.01 | 10.2 | Chr1:4485747:4490158 |
| *Oryza sativa* | DEAD-box | LOC_Os01g10050.1 | 495 | 1488 | 54674.15 | 8.27 | Chr1:5247149:5251192 |
| *Oryza sativa* | DEAD-box | LOC_Os01g10050.2 | 495 | 1488 | 54674.15 | 8.27 | Chr1:5246503:5251192 |
| *Oryza sativa* | DEAD-box | LOC_Os01g36860.1 | 793 | 2382 | 87979.72 | 10 | Chr1:20522447:20528243 |
| *Oryza sativa* | DEAD-box | LOC_Os01g36860.2 | 652 | 1959 | 72096.48 | 10.13 | Chr1:20523343:20528243 |
| *Oryza sativa* | DEAD-box | LOC_Os01g36860.3 | 584 | 1755 | 64797.94 | 10.24 | Chr1:20523343:20528243 |
| *Oryza sativa* | DEAD-box | LOC_Os01g43120.1 | 595 | 1788 | 65634.79 | 9.62 | Chr1:24598181:24601892 |
| *Oryza sativa* | DEAD-box | LOC_Os01g43130.1 | 537 | 1614 | 58849.05 | 9.45 | Chr1:24602225:24605528 |
| *Oryza sativa* | DEAD-box | LOC_Os01g45190.1 | 405 | 1218 | 45598.62 | 5.98 | Chr1:25636950:25640563 |
| *Oryza sativa* | DEAD-box | LOC_Os01g45190.3 | 287 | 864 | 32972.27 | 6.61 | Chr1:25636958:25640563 |
| *Oryza sativa* | DEAD-box | LOC_Os01g62100.1 | 1803 | 5412 | 202672.7 | 6.31 | Chr1:35940240:35949471 |
| *Oryza sativa* | DEAD-box | LOC_Os01g68320.1 | 667 | 2004 | 72188.86 | 9.74 | Chr1:39688260:39693159 |
| *Oryza sativa* | DEAD-box | LOC_Os01g68320.2 | 667 | 2004 | 72188.86 | 9.74 | Chr1:39688261:39693159 |
| *Oryza sativa* | DEAD-box | LOC_Os01g68320.3 | 573 | 1722 | 62103.66 | 9.52 | Chr1:39688291:39693159 |
| *Oryza sativa* | DEAD-box | LOC_Os01g68320.4 | 573 | 1722 | 62103.66 | 9.52 | Chr1:39688261:39693159 |
| *Oryza sativa* | DEAD-box | LOC_Os01g68320.5 | 573 | 1722 | 62103.66 | 9.52 | Chr1:39688260:39693159 |
| *Oryza sativa* | DEAD-box | LOC_Os02g05330.1 | 415 | 1248 | 47137.9 | 5.43 | Chr2:2557627:2561096 |
| *Oryza sativa* | DEAD-box | LOC_Os02g05660.1 | 628 | 1887 | 69242.13 | 6.89 | Chr2:2766217:2770021 |
| *Oryza sativa* | DEAD-box | LOC_Os02g12840.1 | 882 | 2649 | 98476.34 | 5.56 | Chr2:6771313:6778383 |
| *Oryza sativa* | DEAD-box | LOC_Os02g42860.1 | 509 | 1530 | 58166.51 | 8.83 | Chr2:25767209:25774463 |
| *Oryza sativa* | DEAD-box | LOC_Os02g46450.1 | 1060 | 3183 | 120344.9 | 5.26 | Chr2:28293995:28299880 |
| *Oryza sativa* | DEAD-box | LOC_Os02g46450.2 | 1060 | 3183 | 120344.9 | 5.26 | Chr2:28293995:28299880 |
| *Oryza sativa* | DEAD-box | LOC_Os02g57980.1 | 812 | 2439 | 91802.24 | 9.52 | Chr2:35502083:35507050 |
| *Oryza sativa* | DEAD-box | LOC_Os03g06220.1 | 506 | 1521 | 55854.58 | 5.53 | Chr3:3115966:3120767 |
| *Oryza sativa* | DEAD-box | LOC_Os03g19530.1 | 771 | 2316 | 83185.34 | 5.84 | Chr3:10984210:10988399 |
| *Oryza sativa* | DEAD-box | LOC_Os03g36930.1 | 405 | 1218 | 45628.64 | 5.98 | Chr3:20492378:20496911 |
| *Oryza sativa* | DEAD-box | LOC_Os03g46610.1 | 473 | 1422 | 52479.54 | 8.82 | Chr3:26380706:26385454 |
| *Oryza sativa* | DEAD-box | LOC_Os03g46610.2 | 469 | 1410 | 51610.33 | 5.79 | Chr3:26380709:26385454 |
| *Oryza sativa* | DEAD-box | LOC_Os03g50090.1 | 737 | 2214 | 84521 | 8.92 | Chr3:28568345:28571320 |
| *Oryza sativa* | DEAD-box | LOC_Os03g50090.2 | 737 | 2214 | 84521 | 8.92 | Chr3:28568744:28571320 |
| *Oryza sativa* | DEAD-box | LOC_Os03g50090.3 | 737 | 2214 | 84521 | 8.92 | Chr3:28566998:28571307 |
| *Oryza sativa* | DEAD-box | LOC_Os03g50090.4 | 737 | 2214 | 84521 | 8.92 | Chr3:28566996:28571307 |
| *Oryza sativa* | DEAD-box | LOC_Os03g51900.1 | 671 | 2016 | 74331.86 | 8.61 | Chr3:29766132:29774184 |
| *Oryza sativa* | DEAD-box | LOC_Os03g58810.1 | 591 | 1776 | 66651.05 | 8.83 | Chr3:33488395:33493527 |
| *Oryza sativa* | DEAD-box | LOC_Os03g59050.1 | 638 | 1917 | 67665.53 | 7.72 | Chr3:33612462:33617781 |
| *Oryza sativa* | DEAD-box | LOC_Os03g61220.1 | 759 | 2280 | 81615.35 | 6.12 | Chr3:34765939:34772346 |
| *Oryza sativa* | DEAD-box | LOC_Os03g61220.2 | 758 | 2277 | 81528.27 | 6.12 | Chr3:34765939:34772346 |
| *Oryza sativa* | DEAD-box | LOC_Os04g04390.1 | 1725 | 5178 | 191777.9 | 7.97 | Chr4:2071049:2084671 |
| *Oryza sativa* | DEAD-box | LOC_Os04g43140.1 | 833 | 2502 | 93308.55 | 9.31 | Chr4:25535300:25541426 |
| *Oryza sativa* | DEAD-box | LOC_Os04g45040.1 | 499 | 1500 | 56711 | 8.9 | Chr4:26644022:26650268 |
| *Oryza sativa* | DEAD-box | LOC_Os04g45040.2 | 499 | 1500 | 56711 | 8.9 | Chr4:26644075:26650268 |
| *Oryza sativa* | DEAD-box | LOC_Os04g45040.3 | 499 | 1500 | 56711 | 8.9 | Chr4:26644022:26650268 |
| *Oryza sativa* | DEAD-box | LOC_Os05g01990.1 | 592 | 1779 | 66903.56 | 9.56 | Chr5:562876:566821 |
| *Oryza sativa* | DEAD-box | LOC_Os05g01990.2 | 435 | 1308 | 48682.29 | 8.78 | Chr5:562876:565266 |
| *Oryza sativa* | DEAD-box | LOC_Os06g33520.1 | 924 | 2775 | 101104.9 | 8.7 | Chr6:19516630:19524702 |
| *Oryza sativa* | DEAD-box | LOC_Os06g34420.1 | 524 | 1575 | 58891.68 | 9.57 | Chr6:20025972:20031155 |
| *Oryza sativa* | DEAD-box | LOC_Os06g40020.1 | 479 | 1440 | 50808.15 | 9.53 | Chr6:23806007:23811150 |
| *Oryza sativa* | DEAD-box | LOC_Os06g48210.1 | 620 | 1863 | 68143.74 | 6.61 | Chr6:29163789:29166834 |
| *Oryza sativa* | DEAD-box | LOC_Os06g48750.1 | 415 | 1248 | 47087.88 | 5.37 | Chr6:29503679:29507318 |
| *Oryza sativa* | DEAD-box | LOC_Os06g48750.2 | 415 | 1248 | 47087.88 | 5.37 | Chr6:29503679:29507314 |
| *Oryza sativa* | DEAD-box | LOC_Os06g48750.3 | 370 | 1113 | 41913.39 | 6.97 | Chr6:29503679:29507318 |
| *Oryza sativa* | DEAD-box | LOC_Os07g05050.1 | 603 | 1812 | 65127.33 | 9.44 | Chr7:2225622:2231107 |
| *Oryza sativa* | DEAD-box | LOC_Os07g05050.2 | 586 | 1761 | 63227.19 | 9.45 | Chr7:2225622:2231107 |
| *Oryza sativa* | DEAD-box | LOC_Os07g05050.3 | 331 | 996 | 35586.2 | 8.77 | Chr7:2225622:2231107 |
| *Oryza sativa* | DEAD-box | LOC_Os07g10250.1 | 639 | 1920 | 67171.88 | 8.29 | Chr7:5500195:5506490 |
| *Oryza sativa* | DEAD-box | LOC_Os07g20580.1 | 513 | 1542 | 55622.85 | 9.03 | Chr7:11887123:11894422 |
| *Oryza sativa* | DEAD-box | LOC_Os07g33340.1 | 773 | 2322 | 86670.42 | 9.16 | Chr7:19926094:19931994 |
| *Oryza sativa* | DEAD-box | LOC_Os07g43980.1 | 502 | 1509 | 55502.39 | 7.23 | Chr7:26292134:26296155 |
| *Oryza sativa* | DEAD-box | LOC_Os07g46580.1 | 445 | 1338 | 50547.74 | 9.68 | Chr7:27819915:27823763 |
| *Oryza sativa* | DEAD-box | LOC_Os08g05810.1 | 948 | 2847 | 104832.4 | 5.14 | Chr8:3113972:3117296 |
| *Oryza sativa* | DEAD-box | LOC_Os08g06344.1 | 1050 | 3153 | 117731.2 | 5.74 | Chr8:3513828:3518776 |
| *Oryza sativa* | DEAD-box | LOC_Os08g06344.2 | 1050 | 3153 | 117731.2 | 5.74 | Chr8:3513835:3518732 |
| *Oryza sativa* | DEAD-box | LOC_Os08g06344.3 | 1050 | 3153 | 117731.2 | 5.74 | Chr8:3513920:3518732 |
| *Oryza sativa* | DEAD-box | LOC_Os08g32090.1 | 852 | 2559 | 95532.33 | 9.81 | Chr8:19898569:19907743 |
| *Oryza sativa* | DEAD-box | LOC_Os09g19790.1 | 879 | 2640 | 98682.52 | 5.49 | Chr9:11843701:11851263 |
| *Oryza sativa* | DEAD-box | LOC_Os09g21520.1 | 404 | 1215 | 45341.58 | 9.01 | Chr9:13018787:13023173 |
| *Oryza sativa* | DEAD-box | LOC_Os09g21520.2 | 501 | 1506 | 54701.19 | 6.61 | Chr9:13018711:13023099 |
| *Oryza sativa* | DEAD-box | LOC_Os09g34910.1 | 697 | 2094 | 75026.09 | 8.98 | Chr9:20342786:20347227 |
| *Oryza sativa* | DEAD-box | LOC_Os10g35990.1 | 471 | 1416 | 52063.25 | 8.59 | Chr10:19236363:19241930 |
| *Oryza sativa* | DEAD-box | LOC_Os10g35990.2 | 475 | 1428 | 52560.67 | 8.45 | Chr10:19236363:19241930 |
| *Oryza sativa* | DEAD-box | LOC_Os11g38670.1 | 624 | 1875 | 65987.75 | 8.21 | Chr11:22949431:22954688 |
| *Oryza sativa* | DEAD-box | LOC_Os11g46240.1 | 1399 | 4200 | 148859.7 | 9.82 | Chr11:27998365:28005151 |
| *Oryza sativa* | DEAD-box | LOC_Os12g29660.1 | 803 | 2412 | 89507.63 | 5.34 | Chr12:17684779:17694915 |
| *Oryza sativa* | DEAD-box | LOC_Os12g41715.1 | 629 | 1890 | 65594.54 | 9.54 | Chr12:25828364:25832900 |
| *Oryza sativa* | DEAH-box | LOC_Os01g11370.1 | 701 | 2106 | 78631.53 | 7.24 | Chr1:6112801:6125531 |
| *Oryza sativa* | DEAH-box | LOC_Os01g27040.1 | 1108 | 3327 | 127635.7 | 5.21 | Chr1:15075862:15083305 |
| *Oryza sativa* | DEAH-box | LOC_Os01g40980.1 | 1015 | 3048 | 112463.7 | 8.69 | Chr1:23174817:23186976 |
| *Oryza sativa* | DEAH-box | LOC_Os01g40980.2 | 725 | 2178 | 80455.24 | 8.11 | Chr1:23179790:23186976 |
| *Oryza sativa* | DEAH-box | LOC_Os01g65850.1 | 1151 | 3456 | 129570.3 | 6.25 | Chr1:38228812:38237228 |
| *Oryza sativa* | DEAH-box | LOC_Os02g02150.1 | 928 | 2787 | 102654.3 | 7.9 | Chr2:646112:649306 |
| *Oryza sativa* | DEAH-box | LOC_Os02g19860.1 | 1241 | 3726 | 140052.2 | 6.16 | Chr2:11668278:11673605 |
| *Oryza sativa* | DEAH-box | LOC_Os02g32570.1 | 857 | 2574 | 95921.16 | 6.85 | Chr2:19294471:19303250 |
| *Oryza sativa* | DEAH-box | LOC_Os02g32570.2 | 608 | 1827 | 68315.85 | 8.28 | Chr2:19296164:19303246 |
| *Oryza sativa* | DEAH-box | LOC_Os02g50370.1 | 1273 | 3822 | 143450.7 | 6.81 | Chr2:30756675:30762768 |
| *Oryza sativa* | DEAH-box | LOC_Os02g52510.1 | 981 | 2946 | 108302.1 | 6.17 | Chr2:30756675:32134832 |
| *Oryza sativa* | DEAH-box | LOC_Os03g19960.1 | 723 | 2172 | 81707.63 | 6.76 | Chr3:11237786:11241604 |
| *Oryza sativa* | DEAH-box | LOC_Os03g19960.2 | 565 | 1698 | 62705.26 | 6.74 | Chr3:11237786:11241604 |
| *Oryza sativa* | DEAH-box | LOC_Os04g09800.1 | 1133 | 3402 | 126301.5 | 8.28 | Chr4:5254741:5260534 |
| *Oryza sativa* | DEAH-box | LOC_Os04g35420.1 | 1175 | 3528 | 132151.5 | 7.85 | Chr4:21547034:21558315 |
| *Oryza sativa* | DEAH-box | LOC_Os04g40970.1 | 927 | 2784 | 101902.5 | 8.91 | Chr4:24312618:24319913 |
| *Oryza sativa* | DEAH-box | LOC_Os04g47830.1 | 882 | 2649 | 99357.9 | 4.97 | Chr4:28379668:28386850 |
| *Oryza sativa* | DEAH-box | LOC_Os05g05260.1 | 759 | 2280 | 85524.45 | 6.37 | Chr5:2584798:2590120 |
| *Oryza sativa* | DEAH-box | LOC_Os05g05780.1 | 1159 | 3480 | 133521.9 | 4.86 | Chr5:2874325:2884781 |
| *Oryza sativa* | DEAH-box | LOC_Os05g13300.1 | 597 | 1794 | 66576.16 | 8.48 | Chr5:7367166:7374483 |
| *Oryza sativa* | DEAH-box | LOC_Os05g15890.1 | 857 | 2574 | 97069.31 | 8.54 | Chr5:8966122:8973309 |
| *Oryza sativa* | DEAH-box | LOC_Os05g32370.1 | 1152 | 3459 | 127634.7 | 5.98 | Chr5:18872317:18875899 |
| *Oryza sativa* | DEAH-box | LOC_Os06g09280.1 | 855 | 2568 | 94741.3 | 9.27 | Chr6:4659377:4667061 |
| *Oryza sativa* | DEAH-box | LOC_Os06g23530.1 | 1085 | 3258 | 121405.3 | 6.43 | Chr6:13723594:13727110 |
| *Oryza sativa* | DEAH-box | LOC_Os07g32430.1 | 1318 | 3957 | 148661.5 | 6.64 | Chr7:19291152:19298873 |
| *Oryza sativa* | DEAH-box | LOC_Os07g41564.1 | 983 | 2952 | 108487.1 | 6.64 | Chr7:24908632:24915944 |
| *Oryza sativa* | DEAH-box | LOC_Os07g44800.1 | 822 | 2469 | 91331.23 | 8.98 | Chr7:26734659:26738534 |
| *Oryza sativa* | DEAH-box | LOC_Os07g46590.1 | 1003 | 3012 | 114289.5 | 8.99 | Chr7:27825779:27834215 |
| *Oryza sativa* | DEAH-box | LOC_Os07g48360.1 | 887 | 2664 | 98239.48 | 6.73 | Chr7:28901448:28908698 |
| *Oryza sativa* | DEAH-box | LOC_Os08g24760.1 | 1052 | 3159 | 120026.3 | 5.57 | Chr8:14981401:14992328 |
| *Oryza sativa* | DEAH-box | LOC_Os09g37920.1 | 1367 | 4104 | 151420.5 | 7.81 | Chr9:21860417:21868166 |
| *Oryza sativa* | DEAH-box | LOC_Os10g31970.1 | 1477 | 4434 | 167348.1 | 5.46 | Chr10:16787617:16801116 |
| *Oryza sativa* | DEAH-box | LOC_Os11g07870.1 | 1489 | 4470 | 164562.3 | 6.32 | Chr11:4021698:4034044 |
| *Oryza sativa* | DEAH-box | LOC_Os11g20554.1 | 709 | 2130 | 79296.73 | 7.58 | Chr11:11908769:11924922 |
| *Oryza sativa* | DEAH-box | LOC_Os11g44910.1 | 589 | 1770 | 65718.9 | 8.41 | Chr11:27179320:27184059 |
| *Oryza sativa* | DEAH-box | LOC_Os11g44910.2 | 583 | 1752 | 64407.51 | 8.51 | Chr11:27179321:27184048 |
| *Oryza sativa* | DEAH-box | LOC_Os11g44910.3 | 487 | 1464 | 53751.18 | 6.76 | Chr11:27180024:27184048 |
| *Oryza sativa* | DEAH-box | LOC_Os11g48090.2 | 598 | 1797 | 67778.19 | 6.45 | Chr11:28991489:29000897 |
| *Oryza sativa* | DEAH-box | LOC_Os11g48090.3 | 666 | 2001 | 74815.71 | 8.51 | Chr11:28989431:29000897 |
| *Oryza sativa* | DEAH-box | LOC_Os11g48090.4 | 557 | 1674 | 62762.97 | 6.55 | Chr11:28989431:29000913 |
| *Oryza sativa* | DEAH-box | LOC_Os11g48090.5 | 708 | 2127 | 79383.04 | 7 | Chr11:28989431:29000913 |
| *Oryza sativa* | DExD/H-box | LOC_Os01g01312.1 | 1188 | 3567 | 132131.8 | 7.23 | Chr1:151409:156500 |
| *Oryza sativa* | DExD/H-box | LOC_Os01g02884.1 | 813 | 2442 | 91373.13 | 8.74 | Chr1:1024184:1030112 |
| *Oryza sativa* | DExD/H-box | LOC_Os01g12340.1 | 2081 | 6246 | 234818.9 | 7.85 | Chr1:6735758:6746298 |
| *Oryza sativa* | DExD/H-box | LOC_Os01g14200.1 | 2514 | 7545 | 282362.8 | 8.43 | Chr1:7953423:7964300 |
| *Oryza sativa* | DExD/H-box | LOC_Os01g14770.1 | 1571 | 4716 | 176459.4 | 8.71 | Chr1:8251639:8263539 |
| *Oryza sativa* | DExD/H-box | LOC_Os01g15300.1 | 1008 | 3027 | 113227.4 | 6.66 | Chr1:8560623:8569631 |
| *Oryza sativa* | DExD/H-box | LOC_Os01g15300.2 | 990 | 2973 | 111087.9 | 6.65 | Chr1:8560623:8569631 |
| *Oryza sativa* | DExD/H-box | LOC_Os01g21820.1 | 1016 | 3051 | 113680.3 | 5.83 | Chr1:12235262:12248213 |
| *Oryza sativa* | DExD/H-box | LOC_Os01g36890.2 | 345 | 1038 | 39062.23 | 6.64 | Chr1:20540829:20547112 |
| *Oryza sativa* | DExD/H-box | LOC_Os01g36920.2 | 345 | 1038 | 39062.23 | 6.64 | Chr1:20570953:20576221 |
| *Oryza sativa* | DExD/H-box | LOC_Os01g44990.1 | 810 | 2433 | 91479.04 | 8.88 | Chr1:25524184:25530680 |
| *Oryza sativa* | DExD/H-box | LOC_Os01g49680.1 | 767 | 2304 | 86665.15 | 8.61 | Chr1:28545584:28553827 |
| *Oryza sativa* | DExD/H-box | LOC_Os01g56190.1 | 1224 | 3675 | 134666.2 | 7.51 | Chr1:32350513:32360765 |
| *Oryza sativa* | DExD/H-box | LOC_Os01g57110.1 | 1229 | 3690 | 136598.8 | 5.15 | Chr1:33000047:33010221 |
| *Oryza sativa* | DExD/H-box | LOC_Os01g57110.2 | 1214 | 3645 | 134718.8 | 5.17 | Chr1:33000047:33010221 |
| *Oryza sativa* | DExD/H-box | LOC_Os01g72310.1 | 1299 | 3900 | 144368.5 | 8.74 | Chr1:41919630:41925302 |
| *Oryza sativa* | DExD/H-box | LOC_Os01g73900.1 | 439 | 1320 | 48590.35 | 9.23 | Chr1:42821164:42825125 |
| *Oryza sativa* | DExD/H-box | LOC_Os02g01740.1 | 2178 | 6537 | 246615.4 | 5.66 | Chr2:410661:418523 |
| *Oryza sativa* | DExD/H-box | LOC_Os02g02290.1 | 2201 | 6606 | 246212.4 | 8.85 | Chr2:748704:759273 |
| *Oryza sativa* | DExD/H-box | LOC_Os02g06592.1 | 564 | 1695 | 62989.24 | 6.09 | Chr2:3313827:3318385 |
| *Oryza sativa* | DExD/H-box | LOC_Os02g10770.2 | 537 | 1614 | 59046.4 | 7.13 | Chr2:5694660:5697834 |
| *Oryza sativa* | DExD/H-box | LOC_Os02g40450.1 | 1200 | 3603 | 134488.8 | 8.12 | Chr2:24518227:24527233 |
| *Oryza sativa* | DExD/H-box | LOC_Os02g42406.1 | 574 | 1725 | 63285.16 | 9.61 | Chr2:25510873:25513587 |
| *Oryza sativa* | DExD/H-box | LOC_Os02g43460.1 | 1440 | 4323 | 161746.5 | 6.08 | Chr2:26232908:26238290 |
| *Oryza sativa* | DExD/H-box | LOC_Os02g48100.1 | 411 | 1236 | 44860.31 | 5.19 | Chr2:29447864:29451446 |
| *Oryza sativa* | DExD/H-box | LOC_Os02g50560.1 | 1180 | 3543 | 133303.6 | 5.35 | Chr2:30872771:30882871 |
| *Oryza sativa* | DExD/H-box | LOC_Os02g50560.2 | 1070 | 3213 | 121091.6 | 5.58 | Chr2:30872771:30882871 |
| *Oryza sativa* | DExD/H-box | LOC_Os02g55260.1 | 522 | 1569 | 58381.54 | 9.36 | Chr2:33838658:33843647 |
| *Oryza sativa* | DExD/H-box | LOC_Os02g55260.2 | 522 | 1569 | 58381.54 | 9.36 | Chr2:33838663:33843632 |
| *Oryza sativa* | DExD/H-box | LOC_Os03g01830.1 | 642 | 1929 | 70803.16 | 9.25 | Chr3:505922:509294 |
| *Oryza sativa* | DExD/H-box | LOC_Os03g02970.1 | 1884 | 5655 | 210202.7 | 6.23 | Chr3:1195075:1204839 |
| *Oryza sativa* | DExD/H-box | LOC_Os03g06920.1 | 1198 | 3597 | 133595.7 | 6.09 | Chr3:3505567:3512883 |
| *Oryza sativa* | DExD/H-box | LOC_Os03g11470.1 | 1127 | 3384 | 129139.4 | 6.38 | Chr3:5919915:5931373 |
| *Oryza sativa* | DExD/H-box | LOC_Os03g12000.1 | 574 | 1725 | 62918.68 | 9.67 | Chr3:6286011:6288451 |
| *Oryza sativa* | DExD/H-box | LOC_Os03g22900.1 | 1458 | 4377 | 165828.1 | 6.87 | Chr3:13236683:13247898 |
| *Oryza sativa* | DExD/H-box | LOC_Os03g38740.1 | 1411 | 4236 | 158493.8 | 6.41 | Chr3:21500676:21515569 |
| *Oryza sativa* | DExD/H-box | LOC_Os03g51230.1 | 850 | 2553 | 95494.99 | 5.36 | Chr3:29305911:29311726 |
| *Oryza sativa* | DExD/H-box | LOC_Os03g51230.2 | 850 | 2553 | 95494.99 | 5.36 | Chr3:29305420:29311726 |
| *Oryza sativa* | DExD/H-box | LOC_Os03g53220.1 | 2145 | 6438 | 242553.7 | 5.79 | Chr3:30523073:30531098 |
| *Oryza sativa* | DExD/H-box | LOC_Os03g53500.1 | 580 | 1743 | 65182.96 | 8.48 | Chr3:30679685:30689230 |
| *Oryza sativa* | DExD/H-box | LOC_Os03g53760.1 | 1151 | 3456 | 129513.6 | 8.82 | Chr3:30815600:30822590 |
| *Oryza sativa* | DExD/H-box | LOC_Os04g02730.1 | 697 | 2094 | 77529.94 | 7.93 | Chr4:1046902:1054004 |
| *Oryza sativa* | DExD/H-box | LOC_Os04g23360.1 | 1619 | 4860 | 182411.7 | 8.08 | Chr4:13345548:13352576 |
| *Oryza sativa* | DExD/H-box | LOC_Os04g35260.1 | 1440 | 4323 | 160222.6 | 6.15 | Chr4:21429477:21445422 |
| *Oryza sativa* | DExD/H-box | LOC_Os04g38630.1 | 735 | 2208 | 82855.83 | 8.07 | Chr4:22947805:22951875 |
| *Oryza sativa* | DExD/H-box | LOC_Os04g53720.1 | 1052 | 3159 | 116270.2 | 5.77 | Chr4:32009476:32016407 |
| *Oryza sativa* | DExD/H-box | LOC_Os04g59620.1 | 615 | 1848 | 70647.09 | 5.91 | Chr4:35445920:35451211 |
| *Oryza sativa* | DExD/H-box | LOC_Os04g59624.1 | 595 | 1788 | 67365.3 | 6.22 | Chr4:35452000:35458149 |
| *Oryza sativa* | DExD/H-box | LOC_Os04g59624.2 | 988 | 2967 | 110498.5 | 6.01 | Chr4:35452000:35459864 |
| *Oryza sativa* | DExD/H-box | LOC_Os04g59624.3 | 791 | 2376 | 89565.47 | 6.59 | Chr4:35452000:35459864 |
| *Oryza sativa* | DExD/H-box | LOC_Os05g05230.1 | 1129 | 3390 | 128276.1 | 6.01 | Chr5:2548592:2559171 |
| *Oryza sativa* | DExD/H-box | LOC_Os05g05810.1 | 873 | 2622 | 96679.26 | 8.69 | Chr5:2906228:2913780 |
| *Oryza sativa* | DExD/H-box | LOC_Os05g05810.2 | 845 | 2538 | 93505.54 | 8.69 | Chr5:2906228:2913774 |
| *Oryza sativa* | DExD/H-box | LOC_Os05g14380.1 | 572 | 1719 | 64353.49 | 9.22 | Chr5:8097837:8103697 |
| *Oryza sativa* | DExD/H-box | LOC_Os05g19050.1 | 1384 | 4155 | 156524.4 | 8.93 | Chr5:11055644:11061569 |
| *Oryza sativa* | DExD/H-box | LOC_Os05g32610.1 | 1446 | 4341 | 161837 | 4.84 | Chr5:19093513:19099217 |
| *Oryza sativa* | DExD/H-box | LOC_Os06g08480.1 | 1251 | 3756 | 144074.1 | 5.82 | Chr6:4167229:4183940 |
| *Oryza sativa* | DExD/H-box | LOC_Os06g14440.1 | 952 | 2859 | 107537.1 | 5.86 | Chr6:8097281:8104472 |
| *Oryza sativa* | DExD/H-box | LOC_Os06g14440.3 | 947 | 2844 | 106977.4 | 5.91 | Chr6:8097281:8104472 |
| *Oryza sativa* | DExD/H-box | LOC_Os06g14440.4 | 947 | 2844 | 106977.4 | 5.91 | Chr6:8097281:8104472 |
| *Oryza sativa* | DExD/H-box | LOC_Os07g23540.1 | 2267 | 6804 | 255117.4 | 5.93 | Chr7:13294809:13305690 |
| *Oryza sativa* | DExD/H-box | LOC_Os07g25390.1 | 967 | 2904 | 108768.4 | 5.96 | Chr7:14505906:14512576 |
| *Oryza sativa* | DExD/H-box | LOC_Os07g31450.1 | 2193 | 6582 | 243660.5 | 6.37 | Chr7:18625785:18638679 |
| *Oryza sativa* | DExD/H-box | LOC_Os07g40730.1 | 1167 | 3504 | 131107.4 | 7.63 | Chr7:24403299:24416683 |
| *Oryza sativa* | DExD/H-box | LOC_Os07g40730.2 | 1163 | 3492 | 130955.4 | 8.08 | Chr7:24403299:24415850 |
| *Oryza sativa* | DExD/H-box | LOC_Os07g40730.3 | 1101 | 3306 | 123774.2 | 8.11 | Chr7:24403299:24416683 |
| *Oryza sativa* | DExD/H-box | LOC_Os07g44210.1 | 748 | 2247 | 83151.21 | 8.98 | Chr7:26416958:26429335 |
| *Oryza sativa* | DExD/H-box | LOC_Os07g45360.1 | 541 | 1626 | 60110.26 | 9.41 | Chr7:27055343:27059643 |
| *Oryza sativa* | DExD/H-box | LOC_Os07g49210.1 | 1875 | 5628 | 212171.5 | 8.77 | Chr7:29465173:29475499 |
| *Oryza sativa* | DExD/H-box | LOC_Os08g12680.1 | 1455 | 4368 | 159726.6 | 5.97 | Chr8:7488653:7510517 |
| *Oryza sativa* | DExD/H-box | LOC_Os08g14610.1 | 1149 | 3450 | 128786.2 | 5.98 | Chr8:8784440:8793976 |
| *Oryza sativa* | DExD/H-box | LOC_Os09g27060.1 | 846 | 2541 | 95206.77 | 5.41 | Chr9:16461604:16467627 |
| *Oryza sativa* | DExD/H-box | LOC_Os10g33275.1 | 1126 | 3381 | 124860.4 | 8.59 | Chr10:17475729:17484170 |
| *Oryza sativa* | DExD/H-box | LOC_Os10g33275.2 | 903 | 2712 | 101332.3 | 7.79 | Chr10:17475463:17484198 |
| *Oryza sativa* | DExD/H-box | LOC_Os10g34430.1 | 1572 | 4719 | 177717.6 | 6.26 | Chr10:18360484:18372591 |
| *Oryza sativa* | DExD/H-box | LOC_Os11g07500.1 | 1004 | 3015 | 112447.3 | 6.12 | Chr11:3801464:3808587 |
| *Oryza sativa* | DExD/H-box | LOC_Os11g07500.2 | 971 | 2916 | 109097.6 | 6.29 | Chr11:3801464:3808587 |
| *Oryza sativa* | DExD/H-box | LOC_Os11g08980.1 | 1097 | 3294 | 122772.7 | 6.08 | Chr11:4762022:4771082 |
| *Oryza sativa* | DExD/H-box | LOC_Os12g19370.1 | 2066 | 6201 | 226640.6 | 7.72 | Chr12:11242571:11260721 |
| *Zea mays* | DEAD-box | GRMZM2G091652_T01 | 615 | 1848 | 64613.4 | 9.25 | Chr6:102624766..102631077 |
| *Zea mays* | DEAD-box | GRMZM2G027995_T01 | 415 | 1248 | 46982.78 | 5.29 | Chr6:87119198..87122939 |
| *Zea mays* | DEAD-box | GRMZM2G027995_T02 | 415 | 1248 | 46982.78 | 5.29 | Chr6:87119221..87123018 |
| *Zea mays* | DEAD-box | GRMZM2G175867_T01 | 614 | 1845 | 66555.43 | 10.18 | Chr6:66125904..66129928 |
| *Zea mays* | DEAD-box | GRMZM2G175867_T02 | 361 | 1086 | 38984.16 | 9.65 | Chr6:66125700..66129928 |
| *Zea mays* | DEAD-box | GRMZM2G362850_T01 | 572 | 1719 | 62473.05 | 9.56 | Chr3:163743655..163748723 |
| *Zea mays* | DEAD-box | GRMZM2G362850_T02 | 474 | 1425 | 52009.56 | 9.28 | Chr3:163743678..163748723 |
| *Zea mays* | DEAD-box | GRMZM2G362850_T03 | 398 | 1197 | 43438.47 | 8.95 | Chr3:163743678..163748723 |
| *Zea mays* | DEAD-box | GRMZM2G143246_T01 | 495 | 1488 | 54612.97 | 8.25 | Chr3:5571644..5577275 |
| *Zea mays* | DEAD-box | GRMZM2G078826_T01 | 803 | 2412 | 88952.74 | 9.86 | Chr3:226476662..226482081 |
| *Zea mays* | DEAD-box | GRMZM2G078826_T02 | 712 | 2139 | 79173.34 | 10.46 | Chr3:226476662..226482060 |
| *Zea mays* | DEAD-box | GRMZM2G156158_T01 | 364 | 1095 | 41859.79 | 9.52 | Chr3:218190631..218195664 |
| *Zea mays* | DEAD-box | GRMZM2G156158_T02 | 561 | 1686 | 61473.83 | 9.57 | Chr3:218190688..218195692 |
| *Zea mays* | DEAD-box | GRMZM2G026371_T01 | 587 | 1764 | 64759.8 | 9.45 | Chr3:218257250..218260736 |
| *Zea mays* | DEAD-box | GRMZM2G026371_T02 | 602 | 1809 | 66501.74 | 9.4 | Chr3:218257202..218260733 |
| *Zea mays* | DEAD-box | GRMZM2G303752_T01 | 295 | 888 | 32121.21 | 6.32 | Chr3:14340437..14341568 |
| *Zea mays* | DEAD-box | GRMZM2G303752_T02 | 295 | 888 | 31607.72 | 8.61 | Chr3:14340437..14346376 |
| *Zea mays* | DEAD-box | GRMZM2G303752_T03 | 642 | 1929 | 71337.28 | 9.28 | Chr3:14340437..14346376 |
| *Zea mays* | DEAD-box | GRMZM2G565140_T01 | 473 | 1422 | 49721.81 | 9.24 | Chr3:122380643..122384884 |
| *Zea mays* | DEAD-box | GRMZM2G066440_T01 | 673 | 2022 | 73590.81 | 9.38 | Chr3:12282658..12289019 |
| *Zea mays* | DEAD-box | GRMZM2G100043_T01 | 575 | 1728 | 63403.4 | 8.93 | Chr7:105609687..105616288 |
| *Zea mays* | DEAD-box | GRMZM2G100043_T02 | 665 | 1998 | 73005.37 | 9.06 | Chr7:105609687..105616288 |
| *Zea mays* | DEAD-box | GRMZM5G804064_T01 | 771 | 2316 | 85998.46 | 6.74 | Chr7:138602921..138619715 |
| *Zea mays* | DEAD-box | GRMZM2G099253_T01 | 418 | 1257 | 45928 | 6.47 | Chr7:167987207..167991228 |
| *Zea mays* | DEAD-box | GRMZM2G076484_T01 | 509 | 1530 | 55301.79 | 9 | Chr7:33089105..33098044 |
| *Zea mays* | DEAD-box | GRMZM2G076484_T02 | 362 | 1089 | 39207.45 | 8.93 | Chr7:33089047..33098041 |
| *Zea mays* | DEAD-box | GRMZM2G149520_T02 | 708 | 2127 | 75455.4 | 9.09 | Chr7:140811300..140816392 |
| *Zea mays* | DEAD-box | GRMZM2G090869_T01 | 769 | 2310 | 83479.79 | 6.09 | Chr9:135079996..135084638 |
| *Zea mays* | DEAD-box | GRMZM2G043724_T01 | 619 | 1860 | 68526.28 | 6.86 | Chr9:130513487..130516619 |
| *Zea mays* | DEAD-box | GRMZM2G041732_T01 | 925 | 2778 | 100929.6 | 8.28 | Chr9:66904788..66924738 |
| *Zea mays* | DEAD-box | GRMZM2G041732_T02 | 855 | 2568 | 93293.85 | 6.9 | Chr9:66904788..66924497 |
| *Zea mays* | DEAD-box | GRMZM2G164956_T01 | 705 | 2118 | 75127.22 | 9.19 | Chr2:194157154..194162823 |
| *Zea mays* | DEAD-box | GRMZM2G164956_T02 | 672 | 2019 | 72095.04 | 8.59 | Chr2:194157154..194162761 |
| *Zea mays* | DEAD-box | GRMZM2G703415_T01 | 1354 | 4065 | 144674.5 | 9.65 | Chr2:231331216..231337987 |
| *Zea mays* | DEAD-box | GRMZM2G703415_T02 | 1299 | 3900 | 138832.3 | 6.31 | Chr2:231331216..231337673 |
| *Zea mays* | DEAD-box | GRMZM2G092327_T01 | 648 | 1947 | 68370.3 | 8.5 | Chr2:231999718..232004980 |
| *Zea mays* | DEAD-box | GRMZM2G092327_T02 | 648 | 1947 | 68370.3 | 8.5 | Chr2:231999718..232004980 |
| *Zea mays* | DEAD-box | GRMZM2G092327_T03 | 448 | 1347 | 47858.2 | 7.72 | Chr2:231999890..232003650 |
| *Zea mays* | DEAD-box | GRMZM2G420865_T01 | 786 | 2361 | 87434.54 | 5.72 | Chr2:193040339..193063547 |
| *Zea mays* | DEAD-box | GRMZM2G420865_T02 | 774 | 2325 | 86211.6 | 6.74 | Chr2:193040343..193063905 |
| *Zea mays* | DEAD-box | GRMZM2G035807_T01 | 644 | 1935 | 67989.86 | 8.69 | Chr2:232093952..232099171 |
| *Zea mays* | DEAD-box | GRMZM2G125935_T01 | 747 | 2244 | 84255.97 | 9.09 | Chr2:203299633..203303304 |
| *Zea mays* | DEAD-box | GRMZM2G125935_T02 | 445 | 1338 | 50100.24 | 9.3 | Chr2:203299633..203303304 |
| *Zea mays* | DEAD-box | GRMZM2G059365_T01 | 843 | 2532 | 94461.44 | 8.94 | Chr2:28394859..28406297 |
| *Zea mays* | DEAD-box | GRMZM2G077125_T01 | 779 | 2340 | 86418.68 | 9.78 | Chr8:136968141..136973138 |
| *Zea mays* | DEAD-box | GRMZM2G403636_T01 | 609 | 1830 | 64562.27 | 7.74 | Chr4:7260920..7265403 |
| *Zea mays* | DEAD-box | GRMZM2G018947_T01 | 408 | 1227 | 45945.84 | 5.98 | Chr4:168987228..168990822 |
| *Zea mays* | DEAD-box | GRMZM2G129007_T01 | 504 | 1515 | 56264.11 | 8.39 | Chr1:225861963..225867088 |
| *Zea mays* | DEAD-box | GRMZM2G129007_T02 | 462 | 1389 | 51474.75 | 8.59 | Chr1:225861963..225867070 |
| *Zea mays* | DEAD-box | GRMZM2G107984_T01 | 612 | 1839 | 63910.95 | 9.48 | Chr1:172936435..172940871 |
| *Zea mays* | DEAD-box | GRMZM2G107984_T02 | 612 | 1839 | 63910.95 | 9.48 | Chr1:172937047..172940878 |
| *Zea mays* | DEAD-box | AC198418.3_FGT005 | 744 | 2235 | 80170.96 | 6.51 | Chr1:295106763..295189976 |
| *Zea mays* | DEAD-box | GRMZM2G059020_T01 | 444 | 1335 | 49146.87 | 8.59 | Chr1:17933389..17954778 |
| *Zea mays* | DEAD-box | GRMZM2G121871_T01 | 767 | 2304 | 83606.86 | 5.81 | Chr1:49722482..49726934 |
| *Zea mays* | DEAD-box | GRMZM2G057394_T01 | 759 | 2280 | 87246.03 | 8.87 | Chr1:266913672..266916510 |
| *Zea mays* | DEAD-box | GRMZM2G057394_T02 | 759 | 2280 | 87246.03 | 8.87 | Chr1:266913828..266916738 |
| *Zea mays* | DEAD-box | GRMZM2G368658_T01 | 416 | 1251 | 47082.31 | 9.51 | Chr1:264092340..264096845 |
| *Zea mays* | DEAD-box | GRMZM2G030659_T01 | 1066 | 3201 | 119781.2 | 5.71 | Chr10:77440717..77445353 |
| *Zea mays* | DEAD-box | GRMZM2G104795_T01 | 604 | 1815 | 68554.86 | 9.39 | Chr10:7561012..7565422 |
| *Zea mays* | DEAD-box | GRMZM2G028366_T01 | 893 | 2682 | 98803.43 | 5.95 | Chr5:155030183..155038462 |
| *Zea mays* | DEAD-box | GRMZM2G133764_T01 | 408 | 1227 | 45959.87 | 5.98 | Chr5:216737253..216741933 |
| *Zea mays* | DEAD-box | GRMZM2G133764_T02 | 408 | 1227 | 45959.87 | 5.98 | Chr5:216737782..216741511 |
| *Zea mays* | DEAD-box | GRMZM2G171801_T01 | 819 | 2460 | 92268.79 | 9.56 | Chr5:216436330..216441161 |
| *Zea mays* | DEAD-box | GRMZM2G116034_T01 | 412 | 1239 | 46693.43 | 5.29 | Chr5:56776697..56780711 |
| *Zea mays* | DEAD-box | GRMZM2G116034_T02 | 415 | 1248 | 46952.76 | 5.29 | Chr5:56777167..56780802 |
| *Zea mays* | DEAD-box | GRMZM2G167347_T01 | 505 | 1518 | 56215.97 | 9.21 | Chr5:154992984..154995830 |
| *Zea mays* | DEAD-box | GRMZM2G170061_T01 | 276 | 831 | 31649.83 | 8.35 | Chr5:188931128..188936460 |
| *Zea mays* | DEAD-box | GRMZM2G123459_T01 | 735 | 2208 | 84274.96 | 9.01 | Chr5:11603270..11605894 |
| *Zea mays* | DEAD-box | GRMZM2G415491_T01 | 746 | 2241 | 80384.22 | 6.37 | Chr5:1932937..1947273 |
| *Zea mays* | DEAD-box | GRMZM2G086116_T01 | 500 | 1503 | 55843.73 | 8.74 | Chr5:23891310..23896229 |
| *Zea mays* | DEAD-box | GRMZM5G897976_T01 | 650 | 1953 | 73631.57 | 8.11 | Chr5:3813229..3818075 |
| *Zea mays* | DEAD-box | GRMZM2G428242_T01 | 1359 | 4080 | 156413.1 | 6.22 | Chr5:198044969..198062488 |
| *Zea mays* | DEAD-box | GRMZM2G428242_T02 | 1359 | 4080 | 156413.1 | 6.22 | Chr5:198044978..198072321 |
| *Zea mays* | DEAD-box | GRMZM2G428242_T03 | 1476 | 4431 | 169394 | 5.85 | Chr5:198045561..198062488 |
| *Zea mays* | DEAD-box | GRMZM2G428242_T04 | 1476 | 4431 | 169394 | 5.85 | Chr5:198045916..198062488 |
| *Zea mays* | DEAD-box | GRMZM2G129554_T02 | 635 | 1908 | 71395.38 | 8.99 | Chr5:9173279..9184056 |
| *Zea mays* | DEAD-box | GRMZM2G075027_T01 | 456 | 1371 | 50451.15 | 8.77 | Chr5:61228811..61235319 |
| *Zea mays* | DEAD-box | GRMZM2G075027_T02 | 435 | 1308 | 48198.46 | 8.55 | Chr5:61228815..61235301 |
| *Zea mays* | DEAD-box | GRMZM2G075027_T03 | 431 | 1296 | 47653.82 | 8.42 | Chr5:61228834..61235287 |
| *Zea mays* | DEAD-box | GRMZM2G075027_T04 | 433 | 1302 | 47821.49 | 7.91 | Chr5:61229007..61235287 |
| *Zea mays* | DEAD-box | GRMZM2G080512_T01 | 330 | 993 | 35839.83 | 9.05 | Chr7:4905015..4907135 |
| *Zea mays* | DEAD-box | GRMZM2G342226_T01 | 873 | 2622 | 98434.68 | 8.53 | Chr4:14094213..14102136 |
| *Zea mays* | DEAD-box | GRMZM2G367714_T01 | 407 | 1224 | 44420.69 | 8.91 | Chr1:1501026..1507458 |
| *Zea mays* | DEAD-box | GRMZM2G125947_T01 | 339 | 1020 | 36713.75 | 8.54 | Chr10:84992369..84994626 |
| *Zea mays* | DEAD-box | GRMZM2G701730_T01 | 1105 | 3318 | 123760.2 | 6.98 | Chr5:97509131..97518211 |
| *Zea mays* | DEAD-box | GRMZM2G007922_T01 | 1253 | 3762 | 143203.9 | 8.86 | Chr9:128958339..128966321 |
| *Zea mays* | DEAH-box | GRMZM2G100067_T01 | 590 | 1773 | 65876.44 | 7.79 | Chr6:134569609..134572737 |
| *Zea mays* | DEAH-box | GRMZM2G154839_T01 | 941 | 2826 | 105001.3 | 8.62 | Chr3:222038583..222060742 |
| *Zea mays* | DEAH-box | GRMZM2G154839_T02 | 620 | 1863 | 69227.84 | 8 | Chr3:222047517..222060742 |
| *Zea mays* | DEAH-box | GRMZM2G305060_T01 | 941 | 2826 | 104758.8 | 6.23 | Chr3:3700709..3722327 |
| *Zea mays* | DEAH-box | GRMZM2G305060_T02 | 912 | 2739 | 101401.8 | 5.97 | Chr3:3700732..3722327 |
| *Zea mays* | DEAH-box | GRMZM2G010085_T01 | 914 | 2745 | 106772.7 | 6.92 | Chr3:68362865..68368995 |
| *Zea mays* | DEAH-box | GRMZM2G148249_T01 | 825 | 2478 | 91510.29 | 8.64 | Chr7:169402979..169407892 |
| *Zea mays* | DEAH-box | GRMZM2G148249_T02 | 562 | 1689 | 62437.43 | 6.49 | Chr7:169402979..169404891 |
| *Zea mays* | DEAH-box | GRMZM2G021233_T01 | 638 | 1917 | 72083.78 | 9.19 | Chr7:152577971..152584722 |
| *Zea mays* | DEAH-box | GRMZM2G021233_T02 | 680 | 2043 | 76924.43 | 9.1 | Chr7:152577971..152584722 |
| *Zea mays* | DEAH-box | GRMZM2G106732_T01 | 899 | 2700 | 99618.73 | 6.7 | Chr7:173422549..173433051 |
| *Zea mays* | DEAH-box | GRMZM2G107987_T01 | 1168 | 3507 | 129849.4 | 8.26 | Chr7:146746188..146769472 |
| *Zea mays* | DEAH-box | GRMZM2G107987_T02 | 631 | 1896 | 69441.21 | 6.98 | Chr7:146746188..146766096 |
| *Zea mays* | DEAH-box | GRMZM2G082205_T01 | 874 | 2625 | 97031.45 | 7.29 | Chr7:164655672..164666733 |
| *Zea mays* | DEAH-box | GRMZM5G855311_T01 | 1720 | 5163 | 192204.6 | 6.76 | Chr7:166165302..166175886 |
| *Zea mays* | DEAH-box | GRMZM2G010342_T01 | 1526 | 4581 | 173296.9 | 6.08 | Chr7:171232455..171251441 |
| *Zea mays* | DEAH-box | GRMZM2G068479_T01 | 723 | 2172 | 82068.09 | 7.23 | Chr9:134305467..134309912 |
| *Zea mays* | DEAH-box | GRMZM2G068479_T02 | 358 | 1077 | 40045.53 | 5.21 | Chr9:134307379..134309912 |
| *Zea mays* | DEAH-box | GRMZM2G480809_T01 | 1451 | 4356 | 162087.3 | 6.35 | Chr2:202283682..202298594 |
| *Zea mays* | DEAH-box | GRMZM2G117060_T01 | 923 | 2772 | 103547.5 | 6.39 | Chr2:17250064..17257109 |
| *Zea mays* | DEAH-box | GRMZM2G117060_T02 | 855 | 2568 | 95986.08 | 6.49 | Chr2:17250826..17257109 |
| *Zea mays* | DEAH-box | GRMZM2G141258_T01 | 1759 | 5280 | 199857.6 | 5.78 | Chr2:215118775..215135374 |
| *Zea mays* | DEAH-box | GRMZM2G060142_T01 | 438 | 1317 | 48714.05 | 5.85 | Chr8:85398753..85415861 |
| *Zea mays* | DEAH-box | AC235535.1_FGT001 | 1114 | 3345 | 127710.1 | 5.09 | Chr8:53547922..53561252 |
| *Zea mays* | DEAH-box | GRMZM2G300375_T01 | 693 | 2082 | 77376.17 | 8.86 | Chr8:160365905..160374292 |
| *Zea mays* | DEAH-box | GRMZM2G300375_T02 | 385 | 1158 | 42841.47 | 9.32 | Chr8:160365905..160369716 |
| *Zea mays* | DEAH-box | GRMZM2G121776_T01 | 640 | 1923 | 71849.46 | 6.65 | Chr4:54542643..54553611 |
| *Zea mays* | DEAH-box | GRMZM2G001160_T01 | 619 | 1860 | 69676.19 | 7.71 | Chr4:2516472..2521251 |
| *Zea mays* | DEAH-box | GRMZM2G030768_T01 | 1194 | 3585 | 132328.6 | 8.52 | Chr4:60805524..60811917 |
| *Zea mays* | DEAH-box | GRMZM2G012453_T01 | 1162 | 3489 | 128894.2 | 7.97 | Chr1:193042026..193045807 |
| *Zea mays* | DEAH-box | GRMZM2G385931_T01 | 558 | 1677 | 63505.02 | 6.31 | Chr1:51285616..51289996 |
| *Zea mays* | DEAH-box | GRMZM2G385931_T02 | 722 | 2169 | 81735.64 | 6.96 | Chr1:51285621..51289996 |
| *Zea mays* | DEAH-box | GRMZM2G385931_T03 | 493 | 1482 | 55631.22 | 6.17 | Chr1:51285641..51289433 |
| *Zea mays* | DEAH-box | GRMZM2G306348_T01 | 1257 | 3774 | 137218.2 | 9.13 | Chr10:126530563..126540360 |
| *Zea mays* | DEAH-box | GRMZM2G097605_T01 | 759 | 2280 | 85653.71 | 6.59 | Chr10:89368914..89374213 |
| *Zea mays* | DEAH-box | GRMZM2G319573_T01 | 751 | 2256 | 84786.89 | 8.21 | Chr5:77260588..77273432 |
| *Zea mays* | DEAH-box | GRMZM2G146041_T01 | 688 | 2067 | 77683.37 | 9.08 | Chr5:21027392..21046589 |
| *Zea mays* | DEAH-box | GRMZM2G146041_T02 | 631 | 1896 | 71214.08 | 8.82 | Chr5:21027392..21046589 |
| *Zea mays* | DEAH-box | GRMZM2G146041_T03 | 661 | 1986 | 75429.66 | 8.85 | Chr5:21030962..21046194 |
| *Zea mays* | DEAH-box | GRMZM2G357923_T01 | 1288 | 3867 | 144885.2 | 8.36 | Chr5:205363090..205368516 |
| *Zea mays* | DEAH-box | GRMZM2G029258_T01 | 1237 | 3714 | 139729 | 6.12 | Chr5:126448201..126453731 |
| *Zea mays* | DEAH-box | GRMZM2G451856_T01 | 266 | 801 | 29921.92 | 8.52 | Chr1:124684308..124690484 |
| *Zea mays* | DEAH-box | GRMZM2G451856_T02 | 368 | 1107 | 41665.98 | 5.92 | Chr1:124684395..124691798 |
| *Zea mays* | DEAH-box | GRMZM2G015190_T01 | 619 | 1860 | 67103.71 | 7.2 | Chr4:104041135..104044803 |
| *Zea mays* | DExD/H-box | GRMZM2G168096_T01 | 2032 | 6099 | 225457.7 | 5.87 | Chr6:167258877..167282795 |
| *Zea mays* | DExD/H-box | GRMZM2G467799_T01 | 2562 | 7689 | 272616.6 | 4.62 | Chr6:118296098..118319094 |
| *Zea mays* | DExD/H-box | GRMZM2G467799_T02 | 2016 | 6051 | 215767.3 | 4.83 | Chr6:118296098..118318694 |
| *Zea mays* | DExD/H-box | GRMZM2G467799_T03 | 2595 | 7788 | 276231.5 | 4.6 | Chr6:118296098..118318489 |
| *Zea mays* | DExD/H-box | GRMZM2G154946_T01 | 1436 | 4311 | 161480.3 | 5.34 | Chr6:144279933..144287167 |
| *Zea mays* | DExD/H-box | GRMZM2G055807_T01 | 703 | 2112 | 79426.63 | 6.83 | Chr3:206858228..206866614 |
| *Zea mays* | DExD/H-box | GRMZM2G055807_T02 | 768 | 2307 | 86439.64 | 8.25 | Chr3:206858228..206866614 |
| *Zea mays* | DExD/H-box | GRMZM2G373175_T01 | 1382 | 4149 | 154666.7 | 9.11 | Chr3:26786448..26809989 |
| *Zea mays* | DExD/H-box | GRMZM2G373175_T02 | 1126 | 3381 | 126327.4 | 8.92 | Chr3:26789042..26809989 |
| *Zea mays* | DExD/H-box | GRMZM2G010085_T02 | 642 | 1929 | 75460.55 | 7.49 | Chr3:68364533..68368935 |
| *Zea mays* | DExD/H-box | GRMZM2G070264_T01 | 1199 | 3600 | 133400.4 | 7.64 | Chr3:32245135..32250561 |
| *Zea mays* | DExD/H-box | GRMZM2G108166_T01 | 1180 | 3543 | 134223.6 | 8.05 | Chr7:174714070..174719060 |
| *Zea mays* | DExD/H-box | GRMZM2G083580_T01 | 293 | 882 | 32788.7 | 6.62 | Chr7:170487805..170494583 |
| *Zea mays* | DExD/H-box | GRMZM2G083580_T02 | 542 | 1629 | 60310.67 | 9.56 | Chr7:170487783..170494489 |
| *Zea mays* | DExD/H-box | GRMZM2G026991_T01 | 2204 | 6615 | 248692.6 | 5.63 | Chr7:168913721..168922316 |
| *Zea mays* | DExD/H-box | GRMZM2G313553_T01 | 767 | 2304 | 86287.83 | 7.85 | Chr7:163214920..163233302 |
| *Zea mays* | DExD/H-box | GRMZM2G126774_T01 | 693 | 2082 | 75716.2 | 8.99 | Chr7:168449142..168468670 |
| *Zea mays* | DExD/H-box | GRMZM2G126774_T02 | 658 | 1977 | 71816.64 | 8.71 | Chr7:168449142..168468670 |
| *Zea mays* | DExD/H-box | GRMZM2G126774_T03 | 619 | 1860 | 67752.2 | 9.26 | Chr7:168449142..168468670 |
| *Zea mays* | DExD/H-box | GRMZM2G126774_T04 | 706 | 2121 | 77150.86 | 9.06 | Chr7:168449142..168468449 |
| *Zea mays* | DExD/H-box | GRMZM2G316191_T01 | 2380 | 7143 | 264678.2 | 7.35 | Chr7:150159272..150178742 |
| *Zea mays* | DExD/H-box | GRMZM2G316191_T02 | 2187 | 6564 | 243301.3 | 6.93 | Chr7:150162771..150178711 |
| *Zea mays* | DExD/H-box | GRMZM2G040995_T01 | 1005 | 3018 | 113750.9 | 6.21 | Chr9:27433269..27441935 |
| *Zea mays* | DExD/H-box | GRMZM2G040995_T02 | 1001 | 3006 | 113333.4 | 6.21 | Chr9:27433425..27441935 |
| *Zea mays* | DExD/H-box | GRMZM2G113267_T01 | 650 | 1953 | 72033.31 | 8.87 | Chr9:154783553..154787106 |
| *Zea mays* | DExD/H-box | GRMZM2G113267_T02 | 656 | 1971 | 72461.75 | 8.87 | Chr9:154783553..154786977 |
| *Zea mays* | DExD/H-box | GRMZM2G420865_T03 | 338 | 1017 | 38743.5 | 10.04 | Chr2:193048461..193063983 |
| *Zea mays* | DExD/H-box | GRMZM2G102625_T01 | 918 | 2757 | 106008.4 | 8.74 | Chr2:210078278..210095785 |
| *Zea mays* | DExD/H-box | GRMZM2G177165_T01 | 780 | 2343 | 88397.6 | 6.21 | Chr2:236352254..236358078 |
| *Zea mays* | DExD/H-box | GRMZM2G430362_T01 | 628 | 1887 | 70415.45 | 9.04 | Chr2:42114006..42115889 |
| *Zea mays* | DExD/H-box | GRMZM2G013283_T01 | 1381 | 4146 | 154526.2 | 6.19 | Chr2:50455257..50509467 |
| *Zea mays* | DExD/H-box | GRMZM2G013283_T02 | 1186 | 3561 | 132578 | 5.72 | Chr2:50455257..50509467 |
| *Zea mays* | DExD/H-box | GRMZM2G138125_T01 | 1049 | 3150 | 116952.3 | 5.85 | Chr2:26489685..26498990 |
| *Zea mays* | DExD/H-box | GRMZM2G138125_T02 | 962 | 2889 | 107355.5 | 5.88 | Chr2:26489685..26498308 |
| *Zea mays* | DExD/H-box | GRMZM2G138125_T03 | 895 | 2688 | 99963.22 | 5.84 | Chr2:26489685..26497686 |
| *Zea mays* | DExD/H-box | GRMZM2G047949_T01 | 980 | 2943 | 109702.9 | 6.05 | Chr8:18967013..18976088 |
| *Zea mays* | DExD/H-box | GRMZM2G088218_T01 | 428 | 1287 | 48421.55 | 5.45 | Chr8:136701685..136720336 |
| *Zea mays* | DExD/H-box | GRMZM2G088218_T02 | 428 | 1287 | 48421.55 | 5.45 | Chr8:136701685..136720336 |
| *Zea mays* | DExD/H-box | GRMZM2G088218_T03 | 447 | 1344 | 50526.03 | 5.59 | Chr8:136701719..136720301 |
| *Zea mays* | DExD/H-box | GRMZM2G088218_T04 | 185 | 558 | 21279.07 | 8.83 | Chr8:136715724..136716627 |
| *Zea mays* | DExD/H-box | GRMZM2G468132_T01 | 986 | 2961 | 109840.5 | 7.76 | Chr8:172891232..172903385 |
| *Zea mays* | DExD/H-box | GRMZM2G090963_T02 | 869 | 2610 | 95497.55 | 8.78 | Chr8:127335528..127349674 |
| *Zea mays* | DExD/H-box | GRMZM2G415538_T01 | 375 | 1128 | 41788.38 | 8.91 | Chr8:156821831..156824708 |
| *Zea mays* | DExD/H-box | GRMZM2G146047_T01 | 982 | 2949 | 110254.7 | 6.41 | Chr8:5119862..5136608 |
| *Zea mays* | DExD/H-box | GRMZM2G146047_T02 | 1008 | 3027 | 112957.7 | 6.41 | Chr8:5119862..5136598 |
| *Zea mays* | DExD/H-box | GRMZM2G146047_T03 | 765 | 2298 | 86101.33 | 6.64 | Chr8:5119862..5135182 |
| *Zea mays* | DExD/H-box | GRMZM5G892645_T01 | 430 | 1293 | 48591.82 | 5.58 | Chr8:78643850..78648764 |
| *Zea mays* | DExD/H-box | GRMZM5G892645_T02 | 430 | 1293 | 48591.82 | 5.58 | Chr8:78643850..78648764 |
| *Zea mays* | DExD/H-box | GRMZM5G892645_T04 | 430 | 1293 | 48591.82 | 5.58 | Chr8:78643849..78648764 |
| *Zea mays* | DExD/H-box | GRMZM5G892645_T05 | 345 | 1038 | 39168.37 | 6.64 | Chr8:78643849..78648764 |
| *Zea mays* | DExD/H-box | GRMZM2G178435_T01 | 1322 | 3969 | 146856.1 | 5.98 | Chr4:150812097..150817512 |
| *Zea mays* | DExD/H-box | GRMZM2G035068_T01 | 931 | 2796 | 104904.3 | 6 | Chr4:206664053..206669821 |
| *Zea mays* | DExD/H-box | GRMZM2G035068_T03 | 803 | 2412 | 90537.12 | 5.63 | Chr4:206664053..206669410 |
| *Zea mays* | DExD/H-box | GRMZM2G346278_T02 | 1202 | 3609 | 134490.7 | 8.65 | Chr4:139986005..140009737 |
| *Zea mays* | DExD/H-box | GRMZM2G449355_T01 | 785 | 2358 | 86726.02 | 8.73 | Chr4:29204115..29211374 |
| *Zea mays* | DExD/H-box | GRMZM2G449355_T02 | 1034 | 3105 | 114157.1 | 7.75 | Chr4:29192832..29211289 |
| *Zea mays* | DExD/H-box | GRMZM2G574858_T01 | 1100 | 3303 | 121956.1 | 6.69 | Chr4:35493121..35547504 |
| *Zea mays* | DExD/H-box | GRMZM2G078275_T01 | 486 | 1461 | 54347.62 | 5.93 | Chr1:275677365..275687971 |
| *Zea mays* | DExD/H-box | GRMZM2G078275_T02 | 578 | 1737 | 64738.43 | 8.5 | Chr1:275677365..275687971 |
| *Zea mays* | DExD/H-box | GRMZM2G078275_T03 | 395 | 1188 | 44449.4 | 6.41 | Chr1:275678480..275685892 |
| *Zea mays* | DExD/H-box | GRMZM2G413853_T01 | 1517 | 4554 | 171164.5 | 6.02 | Chr1:229740501..229757807 |
| *Zea mays* | DExD/H-box | GRMZM2G469162_T01 | 1369 | 4110 | 156619.1 | 8.55 | Chr1:60395572..60406353 |
| *Zea mays* | DExD/H-box | GRMZM2G040762_T01 | 1308 | 3927 | 145998.4 | 5.92 | Chr1:4607185..4614592 |
| *Zea mays* | DExD/H-box | GRMZM2G071025_T01 | 838 | 2517 | 94268.28 | 5.63 | Chr1:270251701..270258419 |
| *Zea mays* | DExD/H-box | GRMZM2G071025_T03 | 620 | 1863 | 70071.79 | 5.79 | Chr1:270252263..270256170 |
| *Zea mays* | DExD/H-box | GRMZM2G399212_T01 | 1151 | 3456 | 127976.9 | 8.51 | Chr1:233184997..233210134 |
| *Zea mays* | DExD/H-box | GRMZM2G097289_T01 | 586 | 1761 | 67204.9 | 6.08 | Chr10:89204856..89210046 |
| *Zea mays* | DExD/H-box | GRMZM2G097289_T02 | 804 | 2415 | 92301.43 | 6.16 | Chr10:89204867..89210046 |
| *Zea mays* | DExD/H-box | GRMZM2G097289_T03 | 803 | 2412 | 92493.93 | 7.61 | Chr10:89204870..89210046 |
| *Zea mays* | DExD/H-box | GRMZM2G393742_T01 | 951 | 2856 | 106536.9 | 6.61 | Chr10:83537975..83543629 |
| *Zea mays* | DExD/H-box | GRMZM2G030873_T01 | 524 | 1575 | 59099.5 | 9.29 | Chr5:213602336..213610738 |
| *Zea mays* | DExD/H-box | GRMZM2G030873_T02 | 524 | 1575 | 59099.5 | 9.29 | Chr5:213602744..213610738 |
| *Zea mays* | DExD/H-box | GRMZM2G163849_T01 | 2072 | 6219 | 232521.5 | 8.44 | Chr5:68873692..68884654 |
| *Zea mays* | DExD/H-box | GRMZM2G163849_T02 | 2230 | 6693 | 248704 | 8.9 | Chr5:68873880..68884654 |
| *Zea mays* | DExD/H-box | GRMZM2G163849_T03 | 2209 | 6630 | 246557.6 | 8.85 | Chr5:68874838..68884654 |
| *Zea mays* | DExD/H-box | GRMZM2G163849_T04 | 1675 | 5028 | 189132.6 | 8.55 | Chr5:68874991..68882612 |
| *Zea mays* | DExD/H-box | GRMZM2G085587_T01 | 540 | 1623 | 59491.94 | 7.82 | Chr5:114088452..114091567 |
| *Zea mays* | DExD/H-box | GRMZM2G085587_T02 | 540 | 1623 | 59491.94 | 7.82 | Chr5:114088452..114091567 |
| *Zea mays* | DExD/H-box | AC198169.4_FGT004 | 592 | 1779 | 64760.54 | 9.42 | Chr5:188389143..188390957 |
| *Zea mays* | DExD/H-box | GRMZM5G811567_T01 | 415 | 1248 | 46135.55 | 9.62 | Chr2:214166764..214174053 |
| *Zea mays* | DExD/H-box | GRMZM2G151944_T01 | 734 | 2205 | 79040.55 | 7.52 | Chr10:20975566..20984510 |
| *Zea mays* | DExD/H-box | GRMZM2G151944_T02 | 236 | 711 | 25208.18 | 7.04 | Chr10:20981632..20984510 |
| *Zea mays* | DExD/H-box | GRMZM2G050882_T01 | 237 | 714 | 26770.9 | 7.13 | Chr10:130059191..130073070 |
| *Zea mays* | DExD/H-box | GRMZM2G466292_T01 | 528 | 1587 | 59810.97 | 9.17 | Chr5:65425225..65434544 |
| *Zea mays* | DExD/H-box | GRMZM2G379804_T01 | 1004 | 3015 | 113806.4 | 7.71 | Chr3:148611100..148614559 |
| *Glycine max* | DEAD-box | Glyma01g01390.1 | 538 | 1617 | 58897.24 | 8.67 | Gm01:1008976..1013477 |
| *Glycine max* | DEAD-box | Glyma01g43960.1 | 1105 | 3318 | 125521.6 | 5.93 | Gm01:54800718..54805730 |
| *Glycine max* | DEAD-box | Glyma01g43960.2 | 1105 | 3318 | 125521.6 | 5.93 | Gm01:54800718..54805730 |
| *Glycine max* | DEAD-box | Glyma02g08510.1 | 374 | 1125 | 40614.46 | 5.49 | Gm02:6647982..6651215 |
| *Glycine max* | DEAD-box | Glyma02g08550.1 | 637 | 1914 | 69069.7 | 9.91 | Gm02:6664414..6672437 |
| *Glycine max* | DEAD-box | Glyma02g08550.2 | 492 | 1479 | 53728.22 | 9.28 | Gm02:6664414..6672437 |
| *Glycine max* | DEAD-box | Glyma02g25240.1 | 758 | 2277 | 85183.28 | 8.99 | Gm02:25963943..25972849 |
| *Glycine max* | DEAD-box | Glyma02g26630.1 | 612 | 1839 | 66314.53 | 8.03 | Gm02:27600085..27614273 |
| *Glycine max* | DEAD-box | Glyma02g26630.2 | 456 | 1371 | 49526.03 | 6.57 | Gm02:27600085..27614273 |
| *Glycine max* | DEAD-box | Glyma02g45030.1 | 596 | 1791 | 63817.65 | 9.52 | Gm02:49443006..49447931 |
| *Glycine max* | DEAD-box | Glyma02g45990.1 | 747 | 2244 | 85026.78 | 8.75 | Gm02:50086949..50091461 |
| *Glycine max* | DEAD-box | Glyma03g00350.1 | 778 | 2337 | 87823.07 | 9.79 | Gm03:148509..153845 |
| *Glycine max* | DEAD-box | Glyma03g01500.1 | 500 | 1503 | 57108.56 | 8.81 | Gm03:1302223..1308405 |
| *Glycine max* | DEAD-box | Glyma03g01500.2 | 475 | 1428 | 54207.19 | 8.55 | Gm03:1302223..1308405 |
| *Glycine max* | DEAD-box | Glyma03g01530.1 | 503 | 1512 | 57539.07 | 8.76 | Gm03:1315129..1321925 |
| *Glycine max* | DEAD-box | Glyma03g01530.2 | 478 | 1437 | 54782.92 | 8.41 | Gm03:1315129..1321925 |
| *Glycine max* | DEAD-box | Glyma03g01690.1 | 626 | 1881 | 70135.78 | 9.32 | Gm03:1456445..1463393 |
| *Glycine max* | DEAD-box | Glyma03g01710.1 | 440 | 1323 | 49256.3 | 9.01 | Gm03:1472654..1479619 |
| *Glycine max* | DEAD-box | Glyma03g37920.1 | 783 | 2352 | 85341.98 | 5.55 | Gm03:44366492..44371447 |
| *Glycine max* | DEAD-box | Glyma03g38550.1 | 772 | 2319 | 83976.06 | 6.77 | Gm03:44884051..44889833 |
| *Glycine max* | DEAD-box | Glyma03g39670.1 | 588 | 1767 | 65895.68 | 6.64 | Gm03:45669673..45673023 |
| *Glycine max* | DEAD-box | Glyma04g05580.1 | 414 | 1245 | 46679.38 | 5.29 | Gm04:4233647..4236774 |
| *Glycine max* | DEAD-box | Glyma05g02590.1 | 613 | 1842 | 66521.86 | 9.96 | Gm05:1970224..1975351 |
| *Glycine max* | DEAD-box | Glyma05g07780.1 | 573 | 1722 | 64463.97 | 9.23 | Gm05:7759372..7764814 |
| *Glycine max* | DEAD-box | Glyma05g08750.1 | 834 | 2505 | 90036.8 | 9.47 | Gm05:8632345..8637651 |
| *Glycine max* | DEAD-box | Glyma05g28770.1 | 615 | 1848 | 66268.91 | 8.23 | Gm05:34533961..34541936 |
| *Glycine max* | DEAD-box | Glyma05g38030.1 | 554 | 1665 | 62932.34 | 9.4 | Gm05:41483829..41486682 |
| *Glycine max* | DEAD-box | Glyma06g05580.1 | 414 | 1245 | 46664.43 | 5.21 | Gm06:3993479..3996668 |
| *Glycine max* | DEAD-box | Glyma07g11880.1 | 487 | 1464 | 54358.04 | 7.63 | Gm07:10146717..10150516 |
| *Glycine max* | DEAD-box | Glyma07g39910.1 | 497 | 1494 | 56224.22 | 6.17 | Gm07:44254557..44257031 |
| *Glycine max* | DEAD-box | Glyma08g01540.1 | 719 | 2160 | 81627.98 | 9.41 | Gm08:970122..974200 |
| *Glycine max* | DEAD-box | Glyma08g10460.1 | 230 | 693 | 25535.6 | 5.86 | Gm08:7589047..7589812 |
| *Glycine max* | DEAD-box | Glyma08g11920.1 | 620 | 1863 | 66718.36 | 7.71 | Gm08:8642900..8649965 |
| *Glycine max* | DEAD-box | Glyma08g17620.1 | 587 | 1764 | 66660.15 | 9.38 | Gm08:13132164..13137760 |
| *Glycine max* | DEAD-box | Glyma08g20300.1 | 422 | 1269 | 47549.45 | 5.28 | Gm08:15341033..15344189 |
| *Glycine max* | DEAD-box | Glyma08g20300.2 | 225 | 678 | 25071.66 | 5.66 | Gm08:15340884..15344172 |
| *Glycine max* | DEAD-box | Glyma08g20300.3 | 414 | 1245 | 46646.3 | 5.36 | Gm08:15340884..15344189 |
| *Glycine max* | DEAD-box | Glyma08g20670.1 | 508 | 1527 | 56252.8 | 8.95 | Gm08:15691055..15697432 |
| *Glycine max* | DEAD-box | Glyma08g26950.1 | 293 | 882 | 33222.96 | 8.08 | Gm08:21283013..21285051 |
| *Glycine max* | DEAD-box | Glyma08g40250.1 | 540 | 1623 | 60070.39 | 7.98 | Gm08:39935991..39956224 |
| *Glycine max* | DEAD-box | Glyma08g41510.1 | 636 | 1911 | 68363.99 | 9.62 | Gm08:41472619..41479582 |
| *Glycine max* | DEAD-box | Glyma09g03560.1 | 1079 | 3240 | 118196.3 | 10.02 | Gm09:2542200..2548764 |
| *Glycine max* | DEAD-box | Glyma09g07530.1 | 414 | 1245 | 46856.61 | 5.46 | Gm09:6437880..6439885 |
| *Glycine max* | DEAD-box | Glyma09g07530.2 | 414 | 1245 | 46856.61 | 5.46 | Gm09:6437056..6440962 |
| *Glycine max* | DEAD-box | Glyma09g07530.3 | 414 | 1245 | 46856.61 | 5.46 | Gm09:6437620..6440962 |
| *Glycine max* | DEAD-box | Glyma09g08370.1 | 540 | 1623 | 61001.31 | 8.97 | Gm09:7553209..7559946 |
| *Glycine max* | DEAD-box | Glyma09g15220.1 | 612 | 1839 | 69720.56 | 9.94 | Gm09:17637524..17646345 |
| *Glycine max* | DEAD-box | Glyma09g15940.1 | 541 | 1626 | 59648.89 | 8.32 | Gm09:18927572..18932766 |
| *Glycine max* | DEAD-box | Glyma09g34390.1 | 538 | 1617 | 59087.59 | 8.92 | Gm09:40772517..40776918 |
| *Glycine max* | DEAD-box | Glyma09g39710.1 | 491 | 1476 | 56659.87 | 8.07 | Gm09:44738924..44749271 |
| *Glycine max* | DEAD-box | Glyma10g15990.1 | 1439 | 4320 | 165540.6 | 8.7 | Gm10:18790551..18804617 |
| *Glycine max* | DEAD-box | Glyma10g24670.1 | 460 | 1383 | 53144.4 | 9.15 | Gm10:32327637..32336684 |
| *Glycine max* | DEAD-box | Glyma10g28100.1 | 737 | 2214 | 80217.32 | 8.09 | Gm10:36894433..36900741 |
| *Glycine max* | DEAD-box | Glyma10g29360.1 | 602 | 1809 | 67142.49 | 9.2 | Gm10:38219318..38226608 |
| *Glycine max* | DEAD-box | Glyma10g38680.1 | 698 | 2097 | 75784.43 | 8.9 | Gm10:46467950..46472813 |
| *Glycine max* | DEAD-box | Glyma11g01430.1 | 1048 | 3147 | 118610.3 | 6.25 | Gm11:852095..856568 |
| *Glycine max* | DEAD-box | Glyma11g31380.1 | 566 | 1701 | 61573.79 | 6.56 | Gm11:32542683..32554055 |
| *Glycine max* | DEAD-box | Glyma11g35640.1 | 590 | 1773 | 66139.81 | 9.04 | Gm11:37274042..37278083 |
| *Glycine max* | DEAD-box | Glyma11g36440.1 | 605 | 1818 | 66019.57 | 8.48 | Gm11:37926240..37934689 |
| *Glycine max* | DEAD-box | Glyma11g36440.2 | 463 | 1392 | 51071.43 | 6.73 | Gm11:37926240..37934689 |
| *Glycine max* | DEAD-box | Glyma13g16570.1 | 414 | 1245 | 46770.47 | 5.44 | Gm13:20499505..20502333 |
| *Glycine max* | DEAD-box | Glyma13g23720.1 | 587 | 1764 | 63722.89 | 8.05 | Gm13:27054351..27057260 |
| *Glycine max* | DEAD-box | Glyma13g42360.1 | 414 | 1245 | 46645.26 | 5.34 | Gm13:42366631..42369871 |
| *Glycine max* | DEAD-box | Glyma14g02750.1 | 744 | 2235 | 84622.27 | 8.76 | Gm14:1739206..1744688 |
| *Glycine max* | DEAD-box | Glyma14g03760.1 | 611 | 1836 | 65371.19 | 9.35 | Gm14:2422577..2427563 |
| *Glycine max* | DEAD-box | Glyma14g14050.1 | 301 | 906 | 34243.18 | 9.3 | Gm14:13809316..13810542 |
| *Glycine max* | DEAD-box | Glyma14g14170.1 | 592 | 1779 | 66002.5 | 9 | Gm14:13881684..13885952 |
| *Glycine max* | DEAD-box | Glyma14g18390.1 | 199 | 600 | 22288.63 | 4.35 | Gm14:20496015..20496859 |
| *Glycine max* | DEAD-box | Glyma15g03020.1 | 414 | 1245 | 46645.26 | 5.34 | Gm15:2108270..2111809 |
| *Glycine max* | DEAD-box | Glyma15g14470.1 | 1112 | 3339 | 121659.1 | 9.97 | Gm15:10936538..10943520 |
| *Glycine max* | DEAD-box | Glyma15g18760.1 | 414 | 1245 | 46898.65 | 5.46 | Gm15:15642553..15646284 |
| *Glycine max* | DEAD-box | Glyma15g18760.2 | 414 | 1245 | 46898.65 | 5.46 | Gm15:15642977..15646284 |
| *Glycine max* | DEAD-box | Glyma15g18760.3 | 414 | 1245 | 46898.65 | 5.46 | Gm15:15642465..15646284 |
| *Glycine max* | DEAD-box | Glyma15g20000.1 | 563 | 1692 | 63828.89 | 9.28 | Gm15:17582136..17587657 |
| *Glycine max* | DEAD-box | Glyma15g41500.1 | 473 | 1422 | 53628.47 | 9.22 | Gm15:48599254..48604271 |
| *Glycine max* | DEAD-box | Glyma16g02880.1 | 720 | 2163 | 79462.53 | 9.46 | Gm16:2466886..2472764 |
| *Glycine max* | DEAD-box | Glyma16g27680.1 | 374 | 1125 | 40541.51 | 6.19 | Gm16:31645124..31647258 |
| *Glycine max* | DEAD-box | Glyma16g34790.1 | 741 | 2226 | 83469.22 | 9.71 | Gm16:37345902..37352096 |
| *Glycine max* | DEAD-box | Glyma17g00860.1 | 673 | 2022 | 77555.82 | 9.07 | Gm17:472167..474648 |
| *Glycine max* | DEAD-box | Glyma17g06110.1 | 414 | 1245 | 46786.43 | 5.45 | Gm17:4317998..4320875 |
| *Glycine max* | DEAD-box | Glyma17g09270.1 | 603 | 1812 | 65467.75 | 9.81 | Gm17:6841390..6846524 |
| *Glycine max* | DEAD-box | Glyma17g12460.1 | 611 | 1836 | 66634.76 | 6.61 | Gm17:9418082..9421026 |
| *Glycine max* | DEAD-box | Glyma17g13230.1 | 576 | 1731 | 64858.65 | 9.43 | Gm17:10109658..10115649 |
| *Glycine max* | DEAD-box | Glyma17g27250.1 | 321 | 966 | 36607.98 | 8.9 | Gm17:28691803..28693840 |
| *Glycine max* | DEAD-box | Glyma18g00370.1 | 592 | 1779 | 64384.1 | 8.25 | Gm18:106614..111054 |
| *Glycine max* | DEAD-box | Glyma18g02760.1 | 590 | 1773 | 65964.55 | 8.94 | Gm18:1784034..1788659 |
| *Glycine max* | DEAD-box | Glyma18g05800.3 | 375 | 1128 | 41123.83 | 6.9 | Gm18:4444261..4450222 |
| *Glycine max* | DEAD-box | Glyma18g11950.1 | 759 | 2280 | 85165.84 | 8.74 | Gm18:10751625..10760473 |
| *Glycine max* | DEAD-box | Glyma18g14670.1 | 627 | 1884 | 66577.44 | 9.65 | Gm18:14518232..14525811 |
| *Glycine max* | DEAD-box | Glyma18g22940.1 | 543 | 1632 | 61048.2 | 9.32 | Gm18:26290983..26296446 |
| *Glycine max* | DEAD-box | Glyma18g32190.1 | 489 | 1470 | 54280.06 | 5.48 | Gm18:37303911..37308258 |
| *Glycine max* | DEAD-box | Glyma19g00260.1 | 777 | 2334 | 83793.72 | 9.75 | Gm19:70903..75981 |
| *Glycine max* | DEAD-box | Glyma19g03410.1 | 496 | 1491 | 54941.55 | 5.44 | Gm19:3423655..3427462 |
| *Glycine max* | DEAD-box | Glyma19g03410.2 | 413 | 1242 | 45414.75 | 5.24 | Gm19:3423655..3427460 |
| *Glycine max* | DEAD-box | Glyma19g03410.3 | 458 | 1377 | 50474.88 | 5.41 | Gm19:3423655..3427460 |
| *Glycine max* | DEAD-box | Glyma19g24360.1 | 552 | 1659 | 62248.39 | 8.37 | Gm19:29883315..29886884 |
| *Glycine max* | DEAD-box | Glyma19g40510.1 | 769 | 2310 | 83915.51 | 5.72 | Gm19:46889178..46893238 |
| *Glycine max* | DEAD-box | Glyma19g41150.1 | 772 | 2319 | 84185.23 | 7.23 | Gm19:47465990..47471582 |
| *Glycine max* | DEAD-box | Glyma20g11200.1 | 270 | 813 | 30487.66 | 4.82 | Gm20:15744403..15745743 |
| *Glycine max* | DEAD-box | Glyma20g22120.1 | 737 | 2214 | 80155.26 | 7.21 | Gm20:32004711..32010473 |
| *Glycine max* | DEAD-box | Glyma20g29060.1 | 742 | 2229 | 80616.96 | 9.28 | Gm20:38007479..38011928 |
| *Glycine max* | DEAH-box | Glyma01g04790.1 | 766 | 2301 | 87493.57 | 5.93 | Gm01:4376287..4379431 |
| *Glycine max* | DEAH-box | Glyma01g04790.2 | 766 | 2301 | 87493.57 | 5.93 | Gm01:4376888..4379431 |
| *Glycine max* | DEAH-box | Glyma01g07530.1 | 689 | 2070 | 77310.34 | 8.26 | Gm01:8321564..8330922 |
| *Glycine max* | DEAH-box | Glyma01g34350.1 | 1396 | 4191 | 157984.7 | 6.25 | Gm01:46733222..46740763 |
| *Glycine max* | DEAH-box | Glyma01g34350.2 | 807 | 2424 | 90376.85 | 5.86 | Gm01:46735223..46738958 |
| *Glycine max* | DEAH-box | Glyma01g45590.1 | 579 | 1740 | 64519.03 | 7.88 | Gm01:55784579..55788687 |
| *Glycine max* | DEAH-box | Glyma02g01390.1 | 723 | 2172 | 82106.27 | 7.58 | Gm02:996782..1000696 |
| *Glycine max* | DEAH-box | Glyma02g01390.2 | 667 | 2004 | 75031.25 | 6.78 | Gm02:996782..1000696 |
| *Glycine max* | DEAH-box | Glyma02g01390.3 | 682 | 2049 | 77100.54 | 6.78 | Gm02:996782..1000696 |
| *Glycine max* | DEAH-box | Glyma02g09080.1 | 759 | 2280 | 85986.49 | 7.55 | Gm02:7067387..7073901 |
| *Glycine max* | DEAH-box | Glyma02g13170.1 | 651 | 1956 | 73796.47 | 7.62 | Gm02:11390732..11399678 |
| *Glycine max* | DEAH-box | Glyma02g29380.1 | 1968 | 5907 | 224568.7 | 5.19 | Gm02:30961239..30975458 |
| *Glycine max* | DEAH-box | Glyma02g45000.1 | 1767 | 5304 | 201214.4 | 5.59 | Gm02:49400292..49419237 |
| *Glycine max* | DEAH-box | Glyma03g02730.1 | 1054 | 3165 | 118699.6 | 6.7 | Gm03:2535199..2540407 |
| *Glycine max* | DEAH-box | Glyma03g28040.1 | 805 | 2418 | 90637.38 | 8.89 | Gm03:35867328..35871436 |
| *Glycine max* | DEAH-box | Glyma03g37980.1 | 703 | 2112 | 79989.95 | 7.25 | Gm03:44436893..44440366 |
| *Glycine max* | DEAH-box | Glyma07g14470.1 | 293 | 882 | 33304.18 | 8.78 | Gm07:13930197..13932099 |
| *Glycine max* | DEAH-box | Glyma07g19460.1 | 745 | 2238 | 84535.08 | 5.13 | Gm07:19425818..19436651 |
| *Glycine max* | DEAH-box | Glyma07g38050.1 | 1059 | 3180 | 122997.5 | 5.95 | Gm07:42924158..42935132 |
| *Glycine max* | DEAH-box | Glyma07g38050.2 | 968 | 2907 | 112017.8 | 5.42 | Gm07:42924158..42935132 |
| *Glycine max* | DEAH-box | Glyma08g10780.1 | 866 | 2601 | 95972.23 | 8.89 | Gm08:7845997..7853996 |
| *Glycine max* | DEAH-box | Glyma08g20070.1 | 1118 | 3357 | 124911.1 | 8.62 | Gm08:15155657..15163626 |
| *Glycine max* | DEAH-box | Glyma09g08180.1 | 757 | 2274 | 84716.76 | 8.77 | Gm09:7236537..7250193 |
| *Glycine max* | DEAH-box | Glyma09g17220.1 | 2010 | 6033 | 229682.4 | 5.22 | Gm09:21034049..21049833 |
| *Glycine max* | DEAH-box | Glyma09g17220.2 | 2010 | 6033 | 229682.4 | 5.22 | Gm09:21033711..21049833 |
| *Glycine max* | DEAH-box | Glyma09g34860.1 | 691 | 2076 | 78366.86 | 6.64 | Gm09:41157990..41168017 |
| *Glycine max* | DEAH-box | Glyma10g01410.1 | 526 | 1581 | 59009.38 | 6.62 | Gm10:1035853..1038369 |
| *Glycine max* | DEAH-box | Glyma10g28960.1 | 522 | 1569 | 58207.35 | 5.9 | Gm10:37847639..37854025 |
| *Glycine max* | DEAH-box | Glyma11g31710.1 | 382 | 1149 | 43479.07 | 7.28 | Gm11:33057458..33061642 |
| *Glycine max* | DEAH-box | Glyma11g37910.1 | 1737 | 5214 | 196674.5 | 6.85 | Gm11:38876151..38882591 |
| *Glycine max* | DEAH-box | Glyma12g13180.1 | 871 | 2616 | 99036.23 | 7.8 | Gm12:11525193..11531770 |
| *Glycine max* | DEAH-box | Glyma12g30540.1 | 1002 | 3009 | 111758.1 | 6.93 | Gm12:34173867..34183298 |
| *Glycine max* | DEAH-box | Glyma12g31910.1 | 927 | 2784 | 104261.4 | 8.35 | Gm12:35445179..35452589 |
| *Glycine max* | DEAH-box | Glyma13g28720.1 | 1068 | 3207 | 123990.5 | 5.9 | Gm13:31705297..31713779 |
| *Glycine max* | DEAH-box | Glyma13g30610.1 | 737 | 2214 | 82658.28 | 8.42 | Gm13:33171016..33182218 |
| *Glycine max* | DEAH-box | Glyma13g38580.1 | 852 | 2559 | 97229.16 | 9.14 | Gm13:39354063..39361326 |
| *Glycine max* | DEAH-box | Glyma13g41740.1 | 1272 | 3819 | 143585.7 | 6.23 | Gm13:41944763..41954427 |
| *Glycine max* | DEAH-box | Glyma13g43620.1 | 1008 | 3027 | 114286.7 | 8.82 | Gm13:43236390..43246388 |
| *Glycine max* | DEAH-box | Glyma14g03780.1 | 1768 | 5307 | 114286.7 | 8.82 | Gm14:2446855..2465824 |
| *Glycine max* | DEAH-box | Glyma14g39780.1 | 1107 | 3324 | 124912.2 | 6.42 | Gm14:48860701..48875399 |
| *Glycine max* | DEAH-box | Glyma14g40560.1 | 930 | 2793 | 104647.1 | 6.46 | Gm14:49537028..49540409 |
| *Glycine max* | DEAH-box | Glyma15g03660.1 | 1273 | 3822 | 143521.6 | 6.12 | Gm15:2565029..2575253 |
| *Glycine max* | DEAH-box | Glyma15g03660.2 | 1272 | 3819 | 143393.4 | 6.08 | Gm15:2565029..2575196 |
| *Glycine max* | DEAH-box | Glyma15g10370.1 | 1116 | 3351 | 129390.6 | 5.59 | Gm15:7512018..7519956 |
| *Glycine max* | DEAH-box | Glyma16g28290.1 | 759 | 2280 | 86107.58 | 6.88 | Gm16:32274457..32280026 |
| *Glycine max* | DEAH-box | Glyma17g02640.1 | 1060 | 3183 | 123238.9 | 5.91 | Gm17:1720679..1730074 |
| *Glycine max* | DEAH-box | Glyma17g05390.1 | 1010 | 3033 | 112964.3 | 7.92 | Gm17:3735482..3743770 |
| *Glycine max* | DEAH-box | Glyma17g37550.1 | 623 | 1872 | 70372.29 | 7.24 | Gm17:41287119..41289305 |
| *Glycine max* | DEAH-box | Glyma18g00730.1 | 946 | 2841 | 107092.6 | 5.79 | Gm18:326460..329734 |
| *Glycine max* | DEAH-box | Glyma18g01820.1 | 1563 | 4692 | 176332 | 6.39 | Gm18:1022444..1028896 |
| *Glycine max* | DEAH-box | Glyma18g05570.1 | 375 | 1128 | 42338.56 | 5.89 | Gm18:4197685..4201686 |
| *Glycine max* | DEAH-box | Glyma19g40600.1 | 722 | 2169 | 82141.4 | 7 | Gm19:46970138..46973919 |
| *Glycine max* | DEAH-box | Glyma20g00830.1 | 753 | 2262 | 85289.57 | 5 | Gm20:546455..555350 |
| *Glycine max* | DEAH-box | Glyma20g21940.1 | 1076 | 3231 | 121981.5 | 7.92 | Gm20:31730219..31736579 |
| *Glycine max* | DEAH-box | Glyma20g37100.1 | 1574 | 4725 | 179520.1 | 6.45 | Gm20:45031120..45064126 |
| *Glycine max* | DExD/H-box | Glyma01g38150.1 | 763 | 2292 | 87143.71 | 5.63 | Gm01:50297979..50304281 |
| *Glycine max* | DExD/H-box | Glyma01g44610.1 | 419 | 1260 | 47046.46 | 5.64 | Gm01:55221888..55227298 |
| *Glycine max* | DExD/H-box | Glyma02g07540.1 | 516 | 1551 | 56367.17 | 6.13 | Gm02:5954719..5957239 |
| *Glycine max* | DExD/H-box | Glyma02g35240.1 | 1023 | 3072 | 113766.7 | 8.21 | Gm02:39963459..39973271 |
| *Glycine max* | DExD/H-box | Glyma02g42980.1 | 1267 | 3804 | 144919.3 | 8.91 | Gm02:47864329..47869870 |
| *Glycine max* | DExD/H-box | Glyma02g45220.1 | 932 | 2799 | 103321 | 5.65 | Gm02:49555067..49560917 |
| *Glycine max* | DExD/H-box | Glyma03g33590.1 | 538 | 1617 | 61047.3 | 9.36 | Gm03:41113281..41117491 |
| *Glycine max* | DExD/H-box | Glyma03g42290.1 | 1914 | 5745 | 215228.2 | 6.16 | Gm03:47521895..47533485 |
| *Glycine max* | DExD/H-box | Glyma03g42290.2 | 1914 | 5745 | 215228.2 | 6.16 | Gm03:47521895..47533485 |
| *Glycine max* | DExD/H-box | Glyma04g00390.1 | 529 | 1590 | 58915.56 | 9.13 | Gm04:173072..176881 |
| *Glycine max* | DExD/H-box | Glyma04g06630.1 | 1420 | 4263 | 162630 | 5.4 | Gm04:5079767..5099924 |
| *Glycine max* | DExD/H-box | Glyma04g07180.1 | 428 | 1287 | 48261.44 | 5.46 | Gm04:5577335..5582262 |
| *Glycine max* | DExD/H-box | Glyma04g07180.2 | 428 | 1287 | 48261.44 | 5.46 | Gm04:5577335..5582262 |
| *Glycine max* | DExD/H-box | Glyma04g28970.1 | 1314 | 3945 | 149160.6 | 8.14 | Gm04:33058811..33066423 |
| *Glycine max* | DExD/H-box | Glyma04g28970.2 | 1144 | 3435 | 130390.9 | 8.27 | Gm04:33060858..33073888 |
| *Glycine max* | DExD/H-box | Glyma05g26180.1 | 2341 | 7026 | 262262.5 | 8.74 | Gm05:32179593..32205611 |
| *Glycine max* | DExD/H-box | Glyma05g26180.2 | 1684 | 5055 | 188515.1 | 8.59 | Gm05:32194078..32205611 |
| *Glycine max* | DExD/H-box | Glyma05g32740.1 | 569 | 1710 | 64842.73 | 7.36 | Gm05:37573329..37579665 |
| *Glycine max* | DExD/H-box | Glyma05g34180.1 | 1181 | 3546 | 133651 | 8.92 | Gm05:38575844..38585769 |
| *Glycine max* | DExD/H-box | Glyma05g37610.1 | 769 | 2310 | 86773.09 | 8.21 | Gm05:41168276..41176137 |
| *Glycine max* | DExD/H-box | Glyma06g00480.1 | 531 | 1596 | 59142.89 | 9.37 | Gm06:216671..220078 |
| *Glycine max* | DExD/H-box | Glyma06g06720.1 | 1441 | 4326 | 165109.6 | 5.3 | Gm06:4787155..4805471 |
| *Glycine max* | DExD/H-box | Glyma06g06720.2 | 1343 | 4032 | 154523.6 | 5.99 | Gm06:4787155..4805471 |
| *Glycine max* | DExD/H-box | Glyma06g07280.1 | 428 | 1287 | 48275.46 | 5.46 | Gm06:5290379..5295730 |
| *Glycine max* | DExD/H-box | Glyma06g07280.2 | 428 | 1287 | 48275.46 | 5.46 | Gm06:5290379..5295730 |
| *Glycine max* | DExD/H-box | Glyma07g21660.1 | 322 | 969 | 36505.89 | 8.84 | Gm07:22836205..22845800 |
| *Glycine max* | DExD/H-box | Glyma07g38180.1 | 3014 | 9045 | 331430.4 | 6.01 | Gm07:43014196..43036910 |
| *Glycine max* | DExD/H-box | Glyma07g38810.1 | 386 | 1161 | 43072.84 | 8.3 | Gm07:43472183..43477446 |
| *Glycine max* | DExD/H-box | Glyma07g38810.2 | 386 | 1161 | 43072.84 | 8.3 | Gm07:43472183..43477428 |
| *Glycine max* | DExD/H-box | Glyma08g00400.1 | 853 | 2562 | 96423.16 | 5.71 | Gm08:125677..132214 |
| *Glycine max* | DExD/H-box | Glyma08g01970.1 | 769 | 2310 | 86799.08 | 8.05 | Gm08:1295213..1303039 |
| *Glycine max* | DExD/H-box | Glyma08g03130.1 | 977 | 2934 | 110071.7 | 6.34 | Gm08:2206925..2215917 |
| *Glycine max* | DExD/H-box | Glyma08g05480.1 | 1178 | 3537 | 132837.4 | 9.05 | Gm08:3926876..3936230 |
| *Glycine max* | DExD/H-box | Glyma08g09120.1 | 2213 | 6642 | 246436.9 | 7.21 | Gm08:6498845..6511979 |
| *Glycine max* | DExD/H-box | Glyma08g17220.1 | 550 | 1653 | 59829.93 | 9.32 | Gm08:12673941..12675804 |
| *Glycine max* | DExD/H-box | Glyma08g18490.1 | 2184 | 6555 | 247396 | 5.58 | Gm08:13884714..13893085 |
| *Glycine max* | DExD/H-box | Glyma08g22570.1 | 434 | 1305 | 49175.65 | 5.77 | Gm08:17130876..17136077 |
| *Glycine max* | DExD/H-box | Glyma08g22570.2 | 427 | 1284 | 48208.43 | 5.49 | Gm08:17130876..17136077 |
| *Glycine max* | DExD/H-box | Glyma08g24630.1 | 1221 | 3666 | 138020.3 | 8.79 | Gm08:18774464..18802426 |
| *Glycine max* | DExD/H-box | Glyma08g25980.1 | 680 | 2043 | 76352.5 | 5.72 | Gm08:20367628..20374060 |
| *Glycine max* | DExD/H-box | Glyma08g45340.1 | 740 | 2223 | 83970.21 | 6.97 | Gm08:44764453..44767231 |
| *Glycine max* | DExD/H-box | Glyma08g47700.1 | 1052 | 3159 | 119466 | 6.22 | Gm08:46492457..46505705 |
| *Glycine max* | DExD/H-box | Glyma09g02920.1 | 1306 | 3921 | 148093.4 | 7.16 | Gm09:2023777..2035899 |
| *Glycine max* | DExD/H-box | Glyma09g02930.1 | 1415 | 4248 | 160386.2 | 7.38 | Gm09:2041359..2056030 |
| *Glycine max* | DExD/H-box | Glyma09g05810.1 | 408 | 1227 | 46028.98 | 5.89 | Gm09:4560316..4564196 |
| *Glycine max* | DExD/H-box | Glyma09g18490.1 | 802 | 2409 | 90571.72 | 6.52 | Gm09:22909568..22929389 |
| *Glycine max* | DExD/H-box | Glyma09g36910.1 | 2043 | 6132 | 226222.6 | 5.79 | Gm09:42478237..42495590 |
| *Glycine max* | DExD/H-box | Glyma10g10180.1 | 1059 | 3180 | 117771.2 | 8.59 | Gm10:9787147..9797044 |
| *Glycine max* | DExD/H-box | Glyma10g39630.1 | 984 | 2955 | 113698 | 8.71 | Gm10:47254043..47261842 |
| *Glycine max* | DExD/H-box | Glyma11g00640.1 | 1074 | 3225 | 123787.4 | 6.61 | Gm11:297531..304664 |
| *Glycine max* | DExD/H-box | Glyma11g00640.2 | 972 | 2919 | 112396.6 | 6.77 | Gm11:297465..304447 |
| *Glycine max* | DExD/H-box | Glyma11g00970.1 | 599 | 1800 | 67441.1 | 7.6 | Gm11:508237..513963 |
| *Glycine max* | DExD/H-box | Glyma11g07220.1 | 764 | 2295 | 87159.73 | 5.5 | Gm11:5041325..5047545 |
| *Glycine max* | DExD/H-box | Glyma11g21600.1 | 1330 | 3993 | 149845.1 | 8.19 | Gm11:18737810..18746483 |
| *Glycine max* | DExD/H-box | Glyma11g35680.1 | 787 | 2364 | 91059.14 | 8.68 | Gm11:37311459..37314637 |
| *Glycine max* | DExD/H-box | Glyma12g00450.1 | 2047 | 6144 | 226743 | 5.71 | Gm12:169496..190588 |
| *Glycine max* | DExD/H-box | Glyma12g00950.1 | 722 | 2169 | 82724.16 | 7 | Gm12:513023..515859 |
| *Glycine max* | DExD/H-box | Glyma12g36460.1 | 884 | 2655 | 100255.5 | 5.63 | Gm12:39517010..39524097 |
| *Glycine max* | DExD/H-box | Glyma13g05330.1 | 805 | 2418 | 90813.99 | 7.49 | Gm13:5620067..5624438 |
| *Glycine max* | DExD/H-box | Glyma13g17850.1 | 516 | 1551 | 57418.38 | 8.91 | Gm13:21548080..21554764 |
| *Glycine max* | DExD/H-box | Glyma13g18650.1 | 1226 | 3681 | 137380.5 | 6.13 | Gm13:22315596..22322805 |
| *Glycine max* | DExD/H-box | Glyma13g22450.1 | 1395 | 4188 | 157116.9 | 6.32 | Gm13:25960835..25983648 |
| *Glycine max* | DExD/H-box | Glyma13g27170.1 | 824 | 2475 | 93681.94 | 5.6 | Gm13:30342768..30347652 |
| *Glycine max* | DExD/H-box | Glyma14g03530.1 | 844 | 2535 | 93974.2 | 5.31 | Gm14:2300233..2304841 |
| *Glycine max* | DExD/H-box | Glyma14g06090.1 | 1308 | 3927 | 149707.5 | 8.64 | Gm14:4388314..4395992 |
| *Glycine max* | DExD/H-box | Glyma14g20430.1 | 1824 | 5475 | 202177.5 | 7.22 | Gm14:23490206..23516454 |
| *Glycine max* | DExD/H-box | Glyma15g17060.1 | 480 | 1443 | 54040.65 | 6.99 | Gm15:13352055..13358781 |
| *Glycine max* | DExD/H-box | Glyma15g17060.2 | 407 | 1224 | 45971.93 | 5.9 | Gm15:13354389..13358881 |
| *Glycine max* | DExD/H-box | Glyma15g31800.1 | 1150 | 3453 | 126870.2 | 5.91 | Gm15:35290507..35316474 |
| *Glycine max* | DExD/H-box | Glyma15g40460.1 | 2184 | 6555 | 247542.3 | 5.6 | Gm15:47458459..47466873 |
| *Glycine max* | DExD/H-box | Glyma15g41980.1 | 534 | 1605 | 58493.58 | 9.44 | Gm15:49277225..49279117 |
| *Glycine max* | DExD/H-box | Glyma16g03950.1 | 2156 | 6471 | 242319.5 | 8.99 | Gm16:3295367..3305627 |
| *Glycine max* | DExD/H-box | Glyma16g07850.1 | 893 | 2682 | 101374.3 | 8.43 | Gm16:7119319..7126093 |
| *Glycine max* | DExD/H-box | Glyma16g26580.1 | 404 | 1215 | 44047.79 | 9.25 | Gm16:30668269..30670314 |
| *Glycine max* | DExD/H-box | Glyma17g02540.1 | 3217 | 9654 | 350543.5 | 5.38 | Gm17:1618760..1641857 |
| *Glycine max* | DExD/H-box | Glyma17g02540.2 | 3032 | 9099 | 331924.7 | 5.69 | Gm17:1620227..1641857 |
| *Glycine max* | DExD/H-box | Glyma17g04660.1 | 493 | 1482 | 54872.69 | 8.78 | Gm17:3107820..3114311 |
| *Glycine max* | DExD/H-box | Glyma17g11240.1 | 1680 | 5043 | 190239.1 | 7 | Gm17:8436590..8453830 |
| *Glycine max* | DExD/H-box | Glyma17g23720.1 | 367 | 1104 | 42555.8 | 8.52 | Gm17:23885574..23888831 |
| *Glycine max* | DExD/H-box | Glyma17g33260.1 | 1264 | 3795 | 144579.7 | 8.08 | Gm17:36935584..36957583 |
| *Glycine max* | DExD/H-box | Glyma18g02720.1 | 1167 | 3504 | 133991.8 | 8.23 | Gm18:1744224..1748945 |
| *Glycine max* | DExD/H-box | Glyma18g07510.1 | 1349 | 4050 | 151198 | 5.87 | Gm18:6208576..6223756 |
| *Glycine max* | DExD/H-box | Glyma19g02570.1 | 830 | 2493 | 93842.36 | 7.75 | Gm19:2370114..2373832 |
| *Glycine max* | DExD/H-box | Glyma19g36300.1 | 537 | 1614 | 60807.99 | 9.46 | Gm19:43630167..43635384 |
| *Glycine max* | DExD/H-box | Glyma19g36300.2 | 537 | 1614 | 60807.99 | 9.46 | Gm19:43630167..43635451 |
| *Glycine max* | DExD/H-box | Glyma19g45060.1 | 1903 | 5712 | 212269.5 | 6.24 | Gm19:50302036..50314239 |
| *Glycine max* | DExD/H-box | Glyma19g45060.2 | 1903 | 5712 | 212269.5 | 6.24 | Gm19:50302036..50314239 |
| *Glycine max* | DExD/H-box | Glyma20g25800.1 | 1102 | 3309 | 124535.5 | 7.84 | Gm20:35433038..35451434 |
| *Glycine max* | DExD/H-box | Glyma20g28120.1 | 1118 | 3357 | 128962.6 | 7.64 | Gm20:37082388..37094202 |
| *Glycine max* | DExD/H-box | Glyma20g37930.1 | 269 | 810 | 30259.2 | 6.42 | Gm20:45673243..45675887 |
| *Glycine max* | DExD/H-box | Glyma20g37970.1 | 855 | 2568 | 96984.96 | 8.65 | Gm20:45704744..45711404 |
| *Glycine max* | DExD/H-box | Glyma20g37970.2 | 785 | 2358 | 88840.32 | 6.62 | Gm20:45704744..45711404 |
