# Supplementary material for: Genome-Wide Comparative In Silico Analysis of the RNA Helicase Gene Family in Zea mays and Glycine max: A Comparison with Arabidopsis and Oryza sativa
Source: PLoS One. 2013 Nov 12;8(11):e78982. doi: 10.1371/journal.pone.0078982 (PMC3827086; doi:10.1371/journal.pone.0078982)
Supplement: Table S2 — The primers used in the Real-time quantification RT-PCR reactions. (DOC) [file pone.0078982.s006.doc]

Table S2 The primers used in the Real-time quantification RT-PCR reactions.

| Gene name | Forward primer 5’-3’ | Reverse primer 5’-3’ |
| --- | --- | --- |
| *AT1G60930.1* | CTACCTGAGACCTGGCATTAC | ATAGTTGACCCGTGGAACCT |
| *AT1G31360.1* | TGCTGATTATTACCACGCAGAC | GATACATTCGGATGGGAGGC |
| *AT4G35740.1* | AGAGTCTGGTAGAGCAGGTCG | GATGTAGGCTTCTTAGAGGATGAG |
| *AT3G05740.1* | TTCTAATGCCAACGGGAGGG | GGATGAGGGACAACAGAGGAG |
| *AT5G27680.1* | GTGTTGATGATTGGGATGTGG | TATTATCGTGAAAGTCCTGGTG |
| *AT5G05130.1* | AGAGGCAAGAGCAGTGAAAGT | TGAAAGTGACGAGAACGGAGA |
| *AT5G43530.1* | CCTTGCTGGGAGGCTTATCG | CTTGCCATCTGCGTTGCTGT |
| *AT5G22750.1* | AGTGGGCTCGGTGTCTTCTA | CATTGATTCCTCTGCCGTATT |
| *AT1G05120.1* | ACTTTGAGGCGGGATACACT | TCAACCAAGTTAGCATTAGCG |
| *AT1G02670.1* | GAACTATCAGCCGTAAGAGGAG | GAGCAACAGGAGGAACAAGC |
| *Actin* | CTTCGTCTTCCACTTCAG | ATCATACCAGTCTCAACAC |
| *ZM2G113267* | TCAGATGTGTTGTGTTGGATG | AAGAGGAAGAGTGGCAGTC |
| *ZM2G030768* | AATCTAACTGCTGCCTCCAAC | ACCTCTCTTTTCTGCCCAATG |
| *AC235535* | ACAAAGAGAGCAGCACTAAGG | GCACAAGAAACTAAGCACAGC |
| *ZM2G026371* | GACTCCTACTATTGGCACCTTG | TCTGACCGCATTTCTGACTTC |
| *ZM2G071025* | ACATCAGCGACGAAGACC | AGCGACGAGAGCATACCG |
| *ZM2G010085* | GCTGCCTTCCGTATGTCTC | CCTTCCTTGCTTGCCTCTC |
| *ZM2G415491* | CATAGAAGAAGTGCTGGAGTC | GAAGAAGGTGGCTGAGAAAATC |
| *ZM2G133764* | CATCGCATTGGTCGTTCTG | ACATTCATTGGCATCTCGTC |
| *ZM2G106732* | CCACCACCACCAACAAATC | CACTGCTCTGCGGAAATAAG |
| *ZM2G415538* | TGTGATGCTGCTGGAAGAAG | CTGCTCGCTATGTCGGTTG |
| *ZM2G138125* | AATGCTGAGGATGTTGGATGG | GCTTGTATGGCTTGGATGTTG |
| *ZM2G076484* | ATTACTTGCCTTGCTTGAC | GCCTTCCATCCCCTTCTC |
| *ZM2G368658* | TGGCATACAACTCACACTC | AACACTTTCCTCATCTTTCG |
| *EF1-α* | TGATGAGATTGTGAAGGAAGTC | CAGAGATTGGAACGAAGTGG |
